# Amino acid signatures of HLA Class-I and II molecules are strongly associated with SLE susceptibility and autoantibody production in Eastern Asians

**DOI:** 10.1371/journal.pgen.1008092

**Published:** 2019-04-25

**Authors:** Julio E. Molineros, Loren L. Looger, Kwangwoo Kim, Yukinori Okada, Chikashi Terao, Celi Sun, Xu-jie Zhou, Prithvi Raj, Yuta Kochi, Akari Suzuki, Shuji Akizuki, Shuichiro Nakabo, So-Young Bang, Hye-Soon Lee, Young Mo Kang, Chang-Hee Suh, Won Tae Chung, Yong-Beom Park, Jung-Yoon Choe, Seung-Cheol Shim, Shin-Seok Lee, Xiaoxia Zuo, Kazuhiko Yamamoto, Quan-Zhen Li, Nan Shen, Lauren L. Porter, John B. Harley, Kek Heng Chua, Hong Zhang, Edward K. Wakeland, Betty P. Tsao, Sang-Cheol Bae, Swapan K. Nath

**Affiliations:** 1 Arthritis and Clinical Immunology Research Program, Oklahoma Medical Research Foundation, Oklahoma City, Oklahoma, United States of America; 2 Howard Hughes Medical Institute, Janelia Research Campus, Ashburn, Virginia, United States of America; 3 Department of Biology, Kyung Hee University, Seoul, Republic of Korea; 4 Department of Statistical Genetics, Osaka University Graduate School of Medicine, Osaka, Japan; 5 Laboratory for Statistical Analysis, RIKEN Center for Integrative Medical Sciences, Yokohama, Japan; 6 Laboratory of Statistical Immunology, Immunology Frontier Research Center (WPI-IFReC), Osaka University, Suita, Japan; 7 Center for Genomic Medicine, Kyoto University Graduate School of Medicine, Kyoto, Japan; 8 Center for the Promotion of Interdisciplinary Education and Research, Kyoto University, Kyoto, Japan; 9 Division of Genetics, Brigham and Women’s Hospital, Harvard Medical School, Boston, Massachusetts, United States of America; 10 Program in Medical and Population Genetics, Broad Institute, Cambridge, Massachusetts, United States of America; 11 Renal Division, Peking University First Hospital, Peking University Institute of Nephrology, Key Laboratory of Renal Disease, Ministry of Health of China, Beijing, China; 12 Key Laboratory of Chronic Kidney Disease Prevention and Treatment (Peking University), Ministry of Education, Beijing, China; 13 Department of Immunology, University of Texas Southwestern Medical Center, Dallas, Texas, United States of America; 14 Laboratory for Autoimmune Diseases, Center for Integrative Medical Sciences, RIKEN, Yokohama, Japan; 15 Department of Rheumatology and Clinical Immunology, Graduate School of Medicine, Kyoto University, Kyoto, Japan; 16 Department of Rheumatology, Hanyang University Hospital for Rheumatic Diseases, Seoul, Korea; 17 School of Medicine, Kyungpook National University Hospital, Daegu, Korea; 18 Department of Rheumatology, Ajou University Hospital, Suwon, Korea; 19 Dong-A University Hospital, Department of Internal Medicine, Busan, Korea; 20 Department of Internal Medicine, Yonsei University College of Medicine, Seoul, Korea; 21 Department of Rheumatology, Catholic University of Daegu School of Medicine, Daegu, Korea; 22 Daejeon Rheumatoid & Degenerative Arthritis Center, Chungnam National University Hospital, Daejeon, Korea; 23 Department of Rheumatology, Chonnam National University Medical School and Hospital, Gwangju, Korea; 24 Department of Rheumatology and Immunology, Xiangya Hospital, Central South University, Changsha, China; 25 Department of Immunology and Internal Medicine, University of Texas Southwestern Medical Center, Dallas, Texas, United States of America; 26 Department of Rheumatology and Shanghai Institute of Rheumatology, Renji Hospital, Shanghai Jiao Tong University School of Medicine, Shanghai, China; 27 Institute of Health Sciences, Shanghai Institutes for Biological Sciences, Chinese Academy of Sciences and Shanghai Jiao Tong University School of Medicine, Shanghai, China; 28 Center for Autoimmune Genomics and Etiology, Cincinnati Children’s Hospital Medical Center, Cincinnati, Ohio, United States of America; 29 Department of Pediatrics, University of Cincinnati College of Medicine, Cincinnati, Ohio, United States of America; 30 US Department of Veterans Affairs Medical Center, Cincinnati, Ohio, United States of America; 31 Department of Biomedical Science, Faculty of Medicine, University of Malaya, Kuala Lumpur, Malaysia; 32 Division of Rheumatology and Immunology, Department of Medicine, Medical University of South Carolina, Charleston, South Carolina, United States of America; HudsonAlpha Institute for Biotechnology, UNITED STATES

## Abstract

Human leukocyte antigen (HLA) is a key genetic factor conferring risk of systemic lupus erythematosus (SLE), but precise independent localization of HLA effects is extremely challenging. As a result, the contribution of specific HLA alleles and amino-acid residues to the overall risk of SLE and to risk of specific autoantibodies are far from completely understood. Here, we dissected (a) overall SLE association signals across HLA, (b) HLA-peptide interaction, and (c) residue-autoantibody association. Classical alleles, SNPs, and amino-acid residues of eight HLA genes were imputed across 4,915 SLE cases and 13,513 controls from Eastern Asia. We performed association followed by conditional analysis across HLA, assessing both overall SLE risk and risk of autoantibody production. DR15 alleles HLA-DRB1*15:01 (P = 1.4x10^-27^, odds ratio (OR) = 1.57) and HLA-DQB1*06:02 (P = 7.4x10^-23^, OR = 1.55) formed the most significant haplotype (OR = 2.33). Conditioned protein-residue signals were stronger than allele signals and mapped predominantly to *HLA-DRB1* residue 13 (P = 2.2x10^-75^) and its proxy position 11 (P = 1.1x10^-67^), followed by *HLA-DRB1*-37 (P = 4.5x10^-24^). After conditioning on *HLA-DRB1*, novel associations at *HLA-A-*70 (P = 1.4x10^-8^), *HLA-DPB1*-35 (P = 9.0x10^-16^), *HLA-DQB1*-37 (P = 2.7x10^-14^), and *HLA-B*-9 (P = 6.5x10^-15^) emerged. Together, these seven residues increased the proportion of explained heritability due to HLA to 2.6%. Risk residues for both overall disease and hallmark autoantibodies (i.e., nRNP: *DRB1*-11, P = 2.0x10^-14^; *DRB1*-13, P = 2.9x10^-13^; *DRB1*-30, P = 3.9x10^-14^) localized to the peptide-binding groove of *HLA-DRB1*. Enrichment for specific amino-acid characteristics in the peptide-binding groove correlated with overall SLE risk and with autoantibody presence. Risk residues were in primarily negatively charged side-chains, in contrast with rheumatoid arthritis. We identified novel SLE signals in HLA Class I loci (*HLA-A*, *HLA-B*), and localized primary Class II signals to five residues in *HLA-DRB1*, *HLA-DPB1*, and *HLA-DQB1*. These findings provide insights about the mechanisms by which the risk residues interact with each other to produce autoantibodies and are involved in SLE pathophysiology.

## Author summary

The Human leukocyte antigen (HLA) region is a key genetic factor conferring risk of systemic lupus erythematosus (SLE). In spite of multiple SLE association signals identified in the HLA region, only amino-acid residues within *HLA-DRB1* have been specifically described previously. In this study, we performed an imputation-based analysis on individuals with East Asian ancestry, and characterized SLE risk within the HLA region for all involved independent genes (*HLA-DRB1*, *HLA-DPB1*, *HLA-DQB1*, *HLA-A*, and *HLA-B*). Furthermore, we identified a characteristic SLE risk residue signature as well as a pattern of specific nRNP and Ro/La autoantibody residues located in the peptide-binding grooves, suggesting their key involvement in autoantibody production.

## Introduction

Systemic lupus erythematosus (SLE, “lupus”, OMIM 608437) is a complex autoimmune disease disproportionately affecting individuals of East Asian and African ancestry, more frequently and with greater severity[1]. A common characteristic of SLE patients is the production of diverse autoantibodies (*e*.*g*. anti-nuclear antibodies, anti-DNA, anti-Ro/SSA, anti-La/SSB, anti-Sm). SLE has a strong genetic component supported by twin and family studies[2, 3]. Multiple genome-wide and candidate-gene association studies have identified over 80 SLE susceptibility loci, explaining about 30% of narrow-sense SLE heritability[4–6].

Some of the strongest SLE signals are found in the major histocompatibility complex-human leukocyte antigen (MHC-HLA) locus[7, 8]. The MHC region, at 6p21.3, contains >200 genes, including nine classical HLA genes (three Class I: *HLA-A*, *HLA-B*, *HLA-C*; three Class II pairs: *HLA-DPA1/DPB1*, *HLA-DQA1*/*DQB1*, *HLA-DRA1/DRB1*). MHC is implicated in the pathogenesis of every described autoimmune disease, including celiac disease[9], type I diabetes (T1D)[10], rheumatoid arthritis (RA)[11], Sjögren’s syndrome[12], psoriatic arthritis[13], multiple sclerosis (MS)[14], and SLE[8, 15–17]. However, the complexity of the MHC region, stemming from large allelic diversity (*e*.*g*. total known *HLA* alleles are >15,000) and high levels of linkage disequilibrium (LD), has made it challenging to identify the causal basis of disease risk. Much interest has focused on the DR15 haplotype, containing classical alleles HLA-DRB1*15:01, HLA-DQB1*06:02, HLA-DQA1*01:02[18], and HLA-DRB5*01:01[19]. A recent study characterizing SLE risk in the HLA region across European, African, and Hispanic ancestries found notable risk-allele heterogeneity[6] within *DQA1/DQB1* and *DRB1*. In our previous study of SLE in three Asian cohorts[7, 8], we localized strong signals at Class II loci, and confirmed HLA-DRB1*15:01 (primary residues 11-13-26) and HLA-DQB1*06:02[7]. However, other than *HLA-DRB1*, no HLA gene has yet undergone residue mapping. Furthermore, the effects of HLA variants on autoantibody profiles have not been comprehensively investigated.

## Results

### HLA variants associated with SLE susceptibility

**Fig 1:**
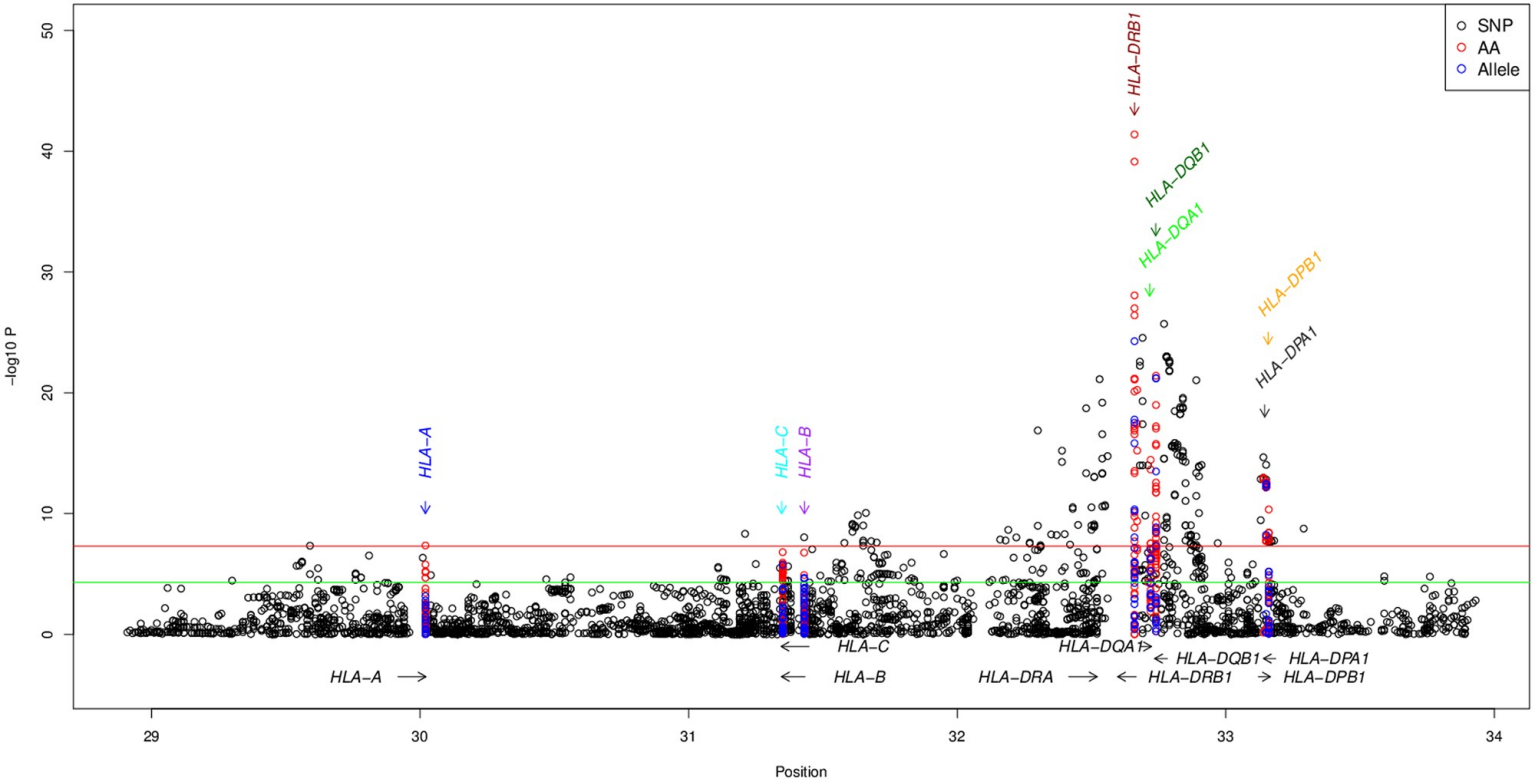
Association of SNPs (black), amino-acids (AA; red), and classical alleles (blue). b. Accumulation of risk residues. Odds ratios estimated as a comparison between the number of risk residues in cases and controls in discovery and replication cohorts.

The strongest SLE association signal was identified within *HLA-DRB1* (Fig 1), consistent with previous observations[7, 8, 20]. HLA-DRB1*15:01 was the strongest associated classical allele (P = 1.4x10^-27^, OR = 1.57; S2 Table) along with tightly correlated (r^2^ = 0.92) HLA-DQB1*06:02 (P = 7.4x10^-23^, OR = 1.55) (both in the extended DR15 haplotype). In concordance with the classical allele results, the strongest SNP association signal occurred close to *HLA-DRB1* (rs9271348, P = 3.5x10^-25^; S3 Table, Fig 1).

Seven HLA genes had ≥ 1 genome-wide significant (GWS) amino-acid residue. Among them, *HLA-DRB1* residue 140-Ala showed the strongest signal (P = 4.1x10^-35^; S4 Table). Because of the great diversity of amino acids at many positions, we analyzed the combined significance (P_omnibus_) of all amino acids at each position (S5 Table). This comprehensive analysis revealed a strong association of residue positions (most significant position *HLA-DRB1-13*, P_omnibus_ = 2.2x10^-75^; and correlated position 11, P_omnibus_ = 1.1x10^-67^; r^2^ = 0.99 11-Pro vs 13-Arg; Table 1); these residues statistically explain SNPs and HLA classical allele signals (S1 and S2 Figs). Conditional logistic regression adjusting for positions 13/11 found additional independent SLE-associated position 37 (S5 Table). Iterative conditional analysis identified six residue positions independently associated with SLE: *HLA-DRB1*-13/11 and 37 (P_conditional_ = 6.8x10^-27^); *HLA-A*-70 (P = 8.9x10^-8^), *HLA-DPB1*-35 (P = 1.4x10^-6^), *HLA-DQB1*-37 (P = 7.2x10^-7^), and *HLA-B*-9 (P = 4.5x10^-5^) (Table 1).

**Table 1:** Independent HLA association. P-values from Log-likelihood ratio tests of the combined model (conditioned on three principal components, sex and ethnic background). KR: Korean; HC1/2: Han Chinese 1/2 (1 has added controls); MC: Malaysian Chinese; JP1/2: Japanese cohorts 1/2. Fisher’s combined P-value is presented to combine individual cohorts’ omnibus-association values. Residues are presented in the order they entered the conditional regression model. Values for *DRB1-11* and *DRB1-13* are unconditioned.

Sequence in Model	Gene Residue	Discovery Sets				Replication Sets				Fisher’s Combined	
		KR	HC1	MC	Discovery	HC2	JP1	JP2	Replication	P unconditioned	P conditioned
*1*	***DRB1-13***	5.86x10^-30^	3.72x10^-8^	1.84x10^-7^	4.12x10^-42^	4.31x10^-18^	7.35x10^-11^	7.02x10^-12^	1.07x10^-37^	2.21x10^-75^	2.21x10^-75^
*1*	***DRB1-11***	1.46x10^-27^	5.25x10^-8^	1.38x10^-6^	7.34x10^-40^	5.03x10^-19^	7.10x10^-9^	1.10x10^-5^	2.56x10^-33^	1.08x10^-67^	1.08x10^-67^
*2*	*DRB1-26*	9.44x10^-14^	4.30x10^-2^	9.24x10^-3^	1.49x10^-9^	2.85x10^-4^	3.46x10^-1^	8.52x10^-1^	2.53x10^-11^	7.28x10^-18^	1.33x10^-16^
*2*	*DRB1-37*	3.89x10^-13^	3.77x10^-7^	3.96x10^-2^	7.42x10^-8^	7.33x10^-8^	2.37x10^-2^	1.69x10^-7^	7.99x10^-15^	4.50x10^-24^	6.80x10^-27^
*3*	*A-70*	2.90x10^-7^	2.41x10^-2^	1.40x10^-1^	4.38x10^-8^	2.81x10^-1^	9.08x10^-2^	2.35x10^-2^	7.54x10^-4^	1.43x10^-8^	8.89x10^-8^
*4*	*DPB1-35*	2.56x10^-7^	8.63x10^-1^	1.59x10^-1^	4.57x10^-11^	5.83x10^-1^	1.34x10^-2^	4.29x10^-1^	7.63x10^-8^	9.00x10^-16^	1.37x10^-6^
*5*	*DQB1-37*	2.33x10^-8^	2.21x10^-1^	2.02x10^-2^	1.74x10^-10^	1.48x10^-2^	1.17x10^-15^	1.68x10^-3^	5.46x10^-6^	2.73x10^-14^	7.21x10^-7^
*6*	*B-9*	3.18x10^-6^	5.22x10^-1^	3.52x10^-1^	1.70x10^-7^	5.27x10^-2^	1.00	4.73x10^-4^	1.29x10^-10^	6.54x10^-15^	4.50x10^-5^

Stepwise conditional analysis adjusting for the entire effect of each amino-acid residue and classical allele at each of *HLA-DRB1*, *HLA-A*, *HLA-DPB1*, *HLA-DQB1*, and *HLA-B* (S1 Fig) both confirmed statistical independence of each of the identified residues and explained the entire association on the HLA region; no other SNP, allele nor residue passed the significant association threshold (P<5x10^-5^; S2a–S2l Fig). Of note is intergenic SNP rs2860580 (close to the 5’ of *HLA-A*), highly correlated with *HLA-A-70* (r^2^ = 0.90), and not significantly stronger than the residue. The effect of this SNP did not replicate across cohorts (S5 Table). Also of note are three HLA-B residues (67, 80 and 81) that passed our conditional association threshold after conditional analysis of all alleles but did not pass the unconditioned association threshold (S2l Fig), and did not replicate.

### Heritability

By integrating the risk residues at the six independent principal positions (*HLA-A-70*, *-B-9*, *DPB1-35*, *DQB1-37*, *DRB1-11/13*, *DRB1-37*), we estimated the proportion of explained heritability due to HLA at 2.6% (S6 Table), higher than our previous estimate[8].

### Accumulation of risk residues

**Fig 2:**
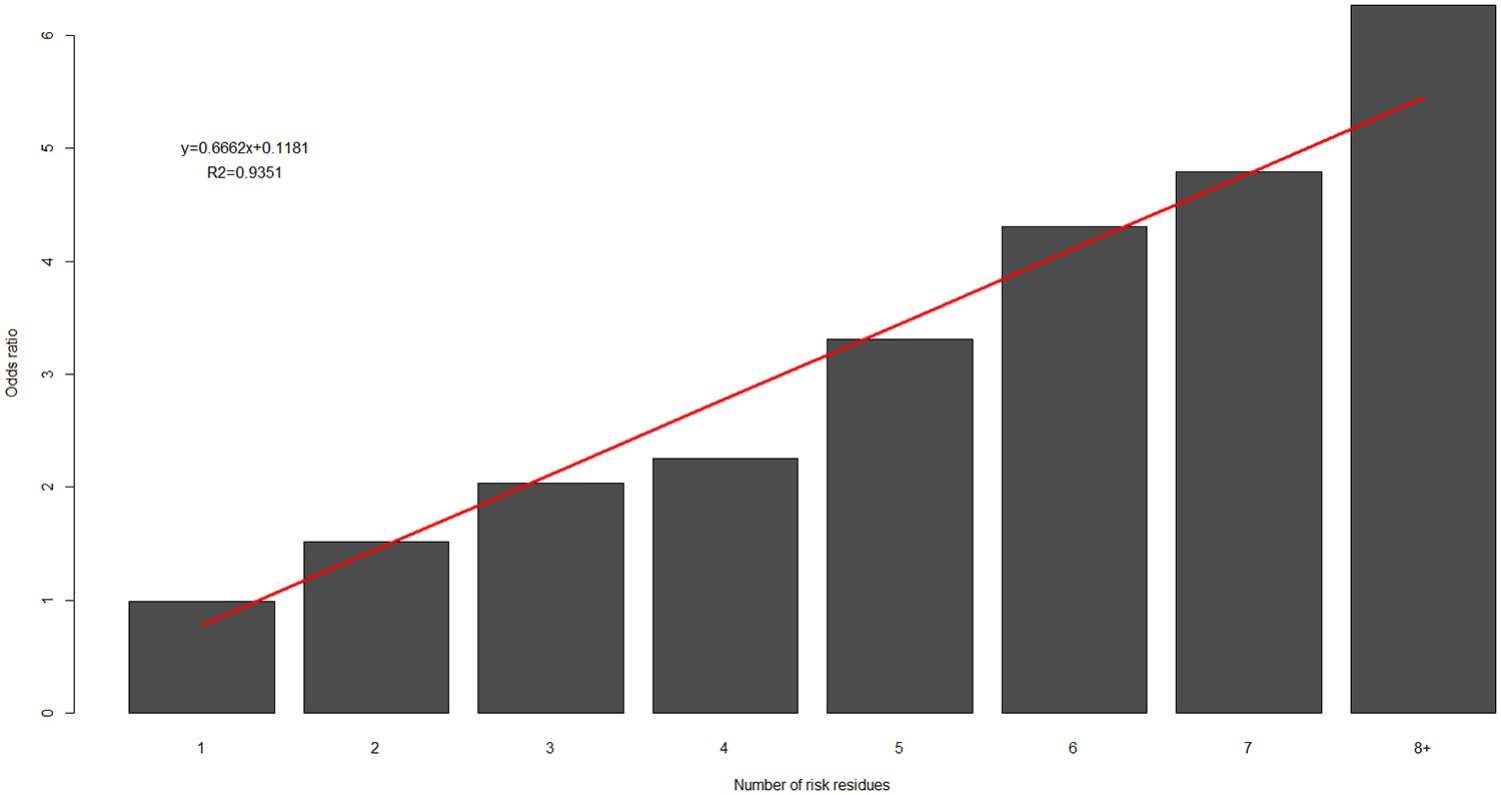
Accumulation of risk residues. Odds ratios estimated as a comparison between the number of risk residues in cases and controls in discovery and replication cohorts.

We observed a significant effect of accumulation of risk residues (P = 8.8x10^-5^) between cases and controls (Fig 2), in both discovery (P_discovery_ = 4.1x10^-5^) and replication (P_replication_ = 1.4x10^-3^) cohorts, and complementary to the accumulation of protective residues in controls/cases (S3 Fig, S7 Table). Risk odds ratios increased linearly, suggesting a model of additive risk. We found no significant difference in overall risk prediction between models using odds-ratio weighted *versus* unweighted risk residue counts (P = 0.45).

### Expression analysis

To assess the impact of each of the seven proposed independent residues on expression of their respective genes, we performed eQTL analysis, followed by conditional analysis of publicly available data (European samples from 1000Genomes). We observed that several SNPs, residues and classical alleles were eQTLs for their respective gene. Following our study framework, conditioning the expression of each gene on its respective classical alleles reduced the signal below the genome-wide significance threshold (P = 5x10^-8^). Interestingly, conditioning on their respective residues did not explain gene expression fully except for *DRB1*. Conditioning for the independent residues (*DRB1*-11+13+37) explained all of *DRB1* expression (S4 Fig).

### Risk residue signatures

SLE risk residue positions (Table 2) mapped overwhelmingly to the peptide-binding groove for each of the eight proteins. Furthermore, risk positions predominantly interacted with the peptide C-terminus, specifically “anchor” positions[21] p4/6 (7/20 most-risk positions over the six major subunits) and p7/9 (9/20). Most risk residues occurred at identical positions among multiple subunits, indicating that residues promoting peptide binding and T-cell receptor activation tend to cluster at several key locations in the interface. Both Class-I (S5 Fig) and Class-II (S6 Fig) subunits showed risk concentrating at a handful of positions binding the peptide C-terminus (S6n Fig). At these positions, the most-risk amino acids tended to be large, hydrophobic or negatively charged (Table 2); only most-risk *DRB1*-13R (in complete LD with *DRB1*-11P) was positively charged.

**Table 2:** Significant mapped SLE risk residues. The most-risk (highest OR; in 19/20 cases, also the most significant) side-chain at each protein position is evaluated according to a simple model (**Supplementary Note**, S10a Table). Side-chains predicted risk include: W, I, F, L, E, Y, D. R, S, N, K, T are predicted to be protective. Residues written as: position, amino acid, position in binding pocket (p1-p9 or peptide-binding 2^nd^ shell), P-value for the discovery cohort, odds ratio. *: *DRB1*-13R is in complete LD with *DRB1*-11P. Residues are formatted according to their consistency with the statistical SLE risk-prediction model: risk (**bold**); protective (*italics*); and neither (unformatted). Amino acid properties: Negatively charged (Asp, D; Glu, E); Positively charged (Arg, R; Lys, K); Potentially positively charged (His, H); Large, hydrophobic (Trp, W; Ile, I; Phe, F; Leu, L; Tyr, Y; Met, M); Medium, hydrophobic (Pro, P; Val, V); Small, hydrophobic (Ala, A; Gly, G; Cys, C); Neutral hydrophilic (Gln, Q; Asn, N; Ser, S; Thr, T).

Subunit	Risk
*A*	70Q (p6; 2x10**-**5, 1.16)
	**97I (p6; 1x10-4, 1.14)**
	**152W (p7; 0.05, 1.19)**
	**114E (p6; 0.05, 1.20)**
*B*	**9D (p2/3; 4x10-5, 2.43)**
	*63N (p1; 6x10-5*, *1*.*12)*
*C*	**9D (p2/3; 7x10-5, 1.14)**
	**147L (p7/9; 1x10-4, 1.15)**
	152A (p7; 1x10-4, 1.15)
*DPB1*	**35Y (p9; 8x10-6, 1.29)**
	**55D (p9; 1x10-7, 1.17)**
*DQB1*	70G (p4, 3x10-15, 1.27)
	**37I (p9; 2x10-5, 1.25)**
	**57D (p9; 0.04, 1.06)**
*DRB1*	*13R (p4/6; 6x10-26*, *1*.43)*
	11P (p6; 6x10-26, 1.43)*
	*37S (p9; 3x10-9*, *1*.*20)*
	**9W (2nd shell; 1x10-25, 1.36)**
	**26Y (p4/6; 1x10-5, 1.17)**
	**67I (p7; 3x10-15, 1.24)**

### Haplotype analysis

The combinations of *DRB1* residues 11Pro-13Arg-26Phe (P = 3.7x10^-22^, OR = 1.56) and 11Pro-13Arg-37Ser (P = 8.5x10^-22^, OR = 1.55) had the strongest risk haplotypes overall (both combinations are present in HLA-DRB1*15 and 16). Notably, including *HLA-B*-9His with either of these haplotypes increased OR to 2.26 (P = 8.7x10^-15^; S8 Table); addition of any other GWS residues did not significantly increase risk. No evidence of interaction was found between significant *DPA1/DPB1* residues, contrary to the observed T1D haplotypic associations[22] at the *DPA/DPB* heterodimer[23]. Haplotype analysis of the four-digit alleles confirmed the strongest risk allele pair as HLA-DRB1*15:01-DQB1*06:02 (P = 2.2x10^-28^, OR [95%CI] = 2.33[1.98–2.76]), and triplet as DRB1*15:01-DQB1*06:02-DPB1*05:01 (P = 2.4x10^-15^, OR [95%CI] = 2.50[1.90–3.28]). DRB1*15:01 and DQB1*06:02 are present in the DR15 haplotype; DPB1*05:01 is separately associated with SLE[24] and is linked to both DRB1*15:01 and DQB1*06:02 in Han Chinese[25].

### Contrasting residues between SLE and rheumatoid arthritis

Specific autoimmune diseases have different features of their autoantigens and thus peptides recognized by the MHC that eventually lead to autoantibody production. For comparison, we also looked at rheumatoid arthritis (RA), where HLA is the predominant risk locus (particularly *HLA-DRB1*), but with different risk alleles and residues^29^. Principal SLE and RA autoantigens were compiled and characterized (S9 Table). SLE autoantigens are overwhelmingly positively charged (S9a Table), whereas RA autoantigens are negatively charged (S9b Table).

A hallmark of RA is the presence of anti-citrullinated protein antibodies (ACPA)[26]. Citrullination deiminates arginine residues (removing their positive charge), leaving the side-chain neutral; poly-citrullinated proteins become very negatively-charged (S9b Table).

Based on these very large differences in autoantigen charge, we developed a simple model to predict the interaction of MHC binding-groove side-chains with largely positively-charged SLE autoantigens and with largely negatively-charged citrullinated RA autoantigens (S1 Text). This model computationally divided the 20 naturally occurring amino acids into: those likely to interact strongly with SLE autoantigens, those likely to interact weakly, and those that are neutral (S10a Table). A similar analysis was performed for RA autoantigens (S10b Table). Importantly the model explicitly convolves peptide-MHC-T-cell receptor tripartite interactions, as it is based on experimental data of immunogenic/non-immunogenic peptides[27]. The model largely contained the set of observed SLE risk amino acids (Table 2), and prominently represented negatively-charged MHC side-chains. Meanwhile, for RA, the model predicted positively-charged MHC side-chains (among others) as risk. In support of these RA-specific predictions, the strongest source of RA risk comes from the *HLA-DRB1* “shared epitope” motif[28] aa-70[(Gln/Arg)-(Lys/Arg)-Arg-Ala-Ala]aa-74, composed almost entirely of predicted RA risk residues. Indeed, upon residue-mapping of MHC risk for RA, the signal primarily localized to *DRB1*-11-Val/Leu, *DRB1*-71-Lys/Arg, *DRB1*-74Ala, *B*-9Asp and *DPB1*-9Phe[29], with 6/7 experimentally determined RA-risk residues coming from the predicted list (S10b Table). The lone protective-predicted residue, *B*-9Asp, is the only one specifically associated with ACPA^-^ RA[30], in which antigens are likely to be more neutrally charged.

### HLA variants associated with autoantibodies

Individual profiles of autoantibodies were available for most SLE (Korean and Han Chinese; S11 Table) patients (n = 2,164). nRNP and Ro/La antibodies had genome-wide significant (P_omnibus_<10^−8^) residue associations (S12 Table). We queried whether the contribution of SLE-associated amino-acid positions and/or other variants was heterogeneous according to autoantibody types.

The strongest risk and protective amino acids for autoantibody association overwhelmingly mapped to Class II loci, particularly *HLA-DRB1* (nRNP, Ro/La and ACL) and *HLA-DPB1* (Sm), in a case-only analysis (Table 3). This is consistent with these MHC subunits playing critical peptide sequence-dependent roles in selection of antigens displayed to the T-cell receptor. Furthermore, all significant residues occupy the middle of the peptide-binding pocket (6/7 most significant residues bind the p4/6 pocket; one binds p7).

**Table 3:** DRB1 risk and protective residues for specific autoantibodies. Significant positions map overwhelmingly to Class II proteins, primarily *DRB1* for nRNP, Ro/La and cardiolipin, and *DPB1* for Sm. Positions with significant association have the most-risk and most-protective amino acids shown. Residues written as: position, amino acid, position in binding pocket, P-value for the Korean and Han Chinese samples, odds ratio. Side-chains predicted risk include: F, L, D. R and S are predicted to be protective. Residues are formatted according to their consistency with the statistical SLE risk-prediction model: risk (**bold**); protective (*italics*); and neither (unformatted).

Antibody	Fraction Class II	Interacting	Significant risk/protection
nRNP	P<1 x10^-3^: 27/27	*DRB1*: P<1 x10^-4^: 7/8	30G (p7; 6 x10-6, 1.71)
			30H (p7; 7x10-4, 0.55)
			**11D (p6; 6x10-6, 1.7)**
			**11S (p6; 2x10-6, 0.68)**
			**13F (p4/6; 6x10-7, 1.65)**
			**13S (p4/6; 4x10-4, 0.69)**
Ro/La	P<5 x10^-3^: 11/11	*DRB1*: P<5 x10^-3^: 5/5	13G (p4/6; 8x10-3, 1.48)
			*13F (p4/6; 7x10-4*, *0*.*61)*
			**70D (p4; 5x10-2, 1.24)**
			**70R (p4; 7x10-4, 0.62)**
Cardiolipin (ACL)	P<5 x10^-3^: 14/14	*DRB1*: P<1 x10^-3^: 3/3	**11D (p6; 8x10-3, 1.69)**
			11V (p6; 1x10-4, 0.44)
Sm	P<1 x10^-2^: 14/18	*DPB1*: P<1 x10^-3^: 3/3	**11L (p4; 1x10-4, 1.52)**
			11G (p4; 1x10-4, 0.66)

We observed significant heterogeneity of nRNP and Ro/La autoantibodies among cases. The most significant association of nRNP autoantibody (683 nRNP^+^ SLE cases; 1,151 nRNP^-^ SLE cases) mapped to *DRB1-11* (P_omnibus_ = 2.0x10^-14^; Table 4), where Leu and Asp contributed to nRNP^+^ risk, while Ser was protective. Positions *DRB1* 13 (P_omnibus_ = 2.9x10^-13^; Phe risk; Ser protective) and 30 (P_omnibus_ = 3.9x10^-14^; Cys, Gly risk; Arg, His protective) also contributed to nRNP^+^ status.

**Table 4:** Anti-nRNP association. OR: Odds-ratio; LCI: Lower confidence limit; UCI: Upper confidence limit.

		Frequency		Asian				
*DRB1*	AA	nRNP+	nRNP-	OR	LCI	UCI	P	P_omnibus_
11	Asp	0.159	0.094	1.82	1.49	2.23	5.26x10^-9^	1.98x10^-14^
	Gly	0.092	0.099	0.97	0.76	1.22	7.75x10^-1^	
	Leu	0.066	0.037	1.89	1.39	2.58	4.85x10^-5^	
	Pro	0.200	0.204	0.98	0.83	1.17	8.51x10^-1^	
	Ser	0.328	0.434	0.62	0.54	0.72	8.90x10^-11^	
	Val	0.156	0.135	1.16	0.96	1.41	1.31x10^-1^	
13	Arg	0.200	0.204	0.98	0.83	1.17	8.52x10^-1^	2.90x10^-13^
	Gly	0.176	0.220	0.74	0.62	0.89	9.53x10^-4^	
	His	0.147	0.118	1.27	1.04	1.56	1.88x10^-2^	
	Phe	0.234	0.147	1.76	1.49	2.09	7.63x10^-11^	
	Ser	0.153	0.214	0.63	0.52	0.76	1.18x10^-6^	
	Tyr	0.092	0.099	0.97	0.76	1.22	7.72x10^-1^	
30	Arg	0.009	0.017	0.49	0.25	0.94	3.33x10^-2^	3.91x10^-14^
	Cis	0.066	0.037	1.89	1.39	2.58	4.96x10^-5^	
	Gly	0.159	0.094	1.82	1.49	2.23	4.93x10^-9^	
	His	0.045	0.082	0.51	0.37	0.69	1.57x10^-5^	
	Leu	0.092	0.099	0.97	0.76	1.22	7.79x10^-1^	
	Tyr	0.630	0.674	0.81	0.7	0.93	3.07x10^-3^	
31	Ile	0.225	0.130	1.92	1.61	2.29	3.11x10^-13^	3.72x10^-13^
	Phe	0.766	0.854	0.57	0.48	0.67	7.39x10^-11^	
	Val	0.009	0.017	0.49	0.25	0.95	3.34x10^-2^	

Analysis of nRNP^+/-^ SLE versus controls found *DRB1* residues 9 and 11 associated (P_omnibus_ = 9.7x10^-25^, 1.4x10^-22^, respectively) with nRNP^+^, and *DRB1-*13 and 11 with nRNP^-^ (P_omnibus_ = 5.1x10^-24^, 6.1x10^-22^). Comparison of amino-acid residue effects between nRNP statuses highlighted the relation between *HLA-DRB1* residues 9Lys (in complete LD with 11Asp), 11Leu/Asp, and 13Phe/His with nRNP status. Both analyses highlighted 11–13, with case^+^/control showing larger effect sizes. There was also significant association of *HLA-DPA1* and *HLA-DPB1* residues with anti-Ro/anti-La autoantibodies (872 Ro/La^+^ and 1,001 Ro/La^-^) particularly at *DPA1*-11 (P_omnibus_ = 2.5x10^-9^; Met risk) and *DPB1*-35 (P_omnibus_ = 3.2x10^-8^; Leu risk) (S12 Table).

Intriguingly, overall SLE, nRNP^+^, and ACL^+^ risk identify different amino acids at the key 11–13 positions, hinting at other autoantibody-specific risk and protective amino acids. Despite the precise residues being different than those for overall SLE risk, amino-acid properties were similar, with 5/7 risk residues coming from the list of six risk-predicted (versus 2 expected); 3/7 protective residues were protective-predicted, while only 1/7 was risk-predicted (Table 3).

Taken together, these results show that MHC subunits, particularly Class II, carry strong, residue-specific risk signals for overall SLE susceptibility and specific antibodies to common SLE antigens. All signals concentrate in the peptide-binding grooves, at sites and with amino-acid properties that broadly overlap between all subunits tested. Many signals are consistent between autoantibody development and SLE risk (*e*.*g*. HLA-DRB1*09:*01* and *DRB1*-11Asp, shared between SLE, nRNP^+^ and ACL^+^ risk; HLA-DRB1*13:*02* and *DRB1*-13Ser, strongly protective for both SLE and nRNP^+^).

## Discussion

In this study, we confirmed SLE associations in six East Asian cohorts for the *HLA-DRB1* locus and identified additional independent Class I and II association signals. The main association signal fell within the same region identified by a meta-analysis on largely European populations[15], with the most-risk alleles (*e*.*g*. DR15) conserved. Amino-acid positions carried significantly more signal than classical alleles (which feature many mutations, only some of which are involved in binding and signaling) and SNPs. Independent residue signals localized to *HLA-DRB1*-13/11-37/26, *DQB1-*37, *DPB1-*35, *B-*9, and *A*-70. The most significant signals in each subunit localized to the peptide-binding groove. *DPB1* was suggested earlier as an SLE risk locus in Japanese[24]; the statistical power of this study confirmed that report in subjects ascertained independently and pinpointed responsible residues. Our diverse cohort set pointed to *DRB1-*37 being equally responsible for the signal previously assigned to *DRB1-*26[7]. In our analysis of the HLA aggregate effect, we identified significant interaction between residues of *DRB1* (11, 13, 26), and Class I *HLA-B* (9) (P = 8.7x10^-15^); the nature of these interactions requires further study and confirmation. In addition, we identified a significant cumulative effect of risk residues, where the increase in risk is positively and incrementally correlated to an increase in the number of risk residues.

Interestingly, we observed that several classical alleles and residues were eQTLs for expression of their own genes. In most cases, compared to the amino acid residues, classical alleles together better explained own-gene expression. Only *DRB1* expression was explainable by amino-acid residues (11, 13 and 37), whereas classical alleles explained the entire expression signals for all genes. However, it is important to note that these observations are based on the imputation and expression data from European samples (1000Genomes) and not from Asians. Additionally, we did not consider a full conditional analysis of expression using SNPs and additional residues. Therefore, the degree to which we can extrapolate these findings on gene expression remains to be determined in future studies.

Most immunogenic peptides are enriched in large, hydrophobic and charged residues[27]. Accordingly, in each HLA subunit, risk residues were typically themselves large and hydrophobic or charged, which would facilitate the best interaction with the peptide to facilitate both MHC binding and T-cell receptor binding and activation. A simple model of MHC-peptide-T-cell receptor interactions was consistent with the observed risk residues. We adapted the model to deal specifically with SLE (autoantigens largely positively-charged) as well as rheumatoid arthritis (autoantigens largely negatively-charged). The model performed well for both diseases, and could prove useful in other studies as well.

Risk positions were frequently conserved between subunits (S6n Fig). In addition to SLE risk coming from identical positions across MHC subunits, other diseases with residue-mapped risk similarly co-localize, *e.g. DRB1*-67 in Type 1 diabetes (T1D)[14] and Sjögren’s syndrome[31] with *A*-152/*C*-152/*DRB1*-67 from this study; *B*-70 and 97 in psoriasis[14] with *A*-70 and *A*-97/*DRB1*-9 from this study; and *DRB1*-11, *B*-9, and *DPB1*-9 in RA[29] with *DRB1*-11, *B*-9/*C*-9, and *DRB1*-9/*A*-97 from this study, respectively. Thus, not only does SLE risk stem from largely predictable amino acids at conserved positions, but this observation extends to other autoimmune diseases as well.

Specific MHC risk alleles and residues have similarities and differences with other autoimmune diseases, *e*.*g*. DRB1*15:01 and DQB1*06:02 are shared between SLE and multiple sclerosis (MS)[14]; whereas DQB1*03:02 confers risk for T1D[14] but protection for SLE. Likewise, *DRB1*-67Ile is strongly risk for SLE but strongly protective for T1D and its subclinical manifestations. Similar amino-acid properties predispose to systemic sclerosis[32]; again, though the specific *DRB1* risk residues are different (*DRB1*-26Phe, 28Asp, 70Asp, 78Tyr). The case is also similar for multiple sclerosis[33] (*DRB1*-57Asp, 71Ala, 74Ala, 86Val; *DPB1*-65Leu), Sjögren’s syndrome (*DRB1*-47Tyr, 67Ile/Leu, 74Leu)[31], and psoriasis[34] (*B*-67Cys/Met, 9Asp, *A*-95Val; *B*-45Glu is associated with psoriatic arthritis). In pemphigus vulgaris (PV), site-directed mutagenesis of either of the DRB1*0402 residues 70Asp and 71Glu to the corresponding DRB1*04:04 residues 70Gln and 71Arg ablated presentation of and immune response to the desmoglein antigen DG(190–204); DRB1*04:02 is risk for PV and DRB1*04:04 is protective[35]. The risk and protective alleles differ only at these two positions, along with Leu67Ile, whose mutation did not greatly affect presentation. Thus, desmoglein antigen presentation and PV risk correspond precisely with the negative charges DRB1-70Asp and 71Glu. Ulcerative colitis (UC) and Crohn’s disease (CD) share these same primary risk residues[36]; furthermore, computed electrostatic surfaces around the DRB1-p4/6 pocket correlated almost exactly with risk across all alleles.

Protein binding affinity is driven largely by hydrophobic interactions, with electrostatics dictating most of the binding specificity[37]. Individual autoantigens present diverse charges that need to be complemented by appropriate MHC and TCR charges. Positive charge at specific residues can be risk for autoimmune (Hashimoto’s) thyroiditis[38] (*DRB1*-26Tyr, 30Tyr, 70Gln, 71Lys, 74Arg), T1D (*DRB1*-13His, 67Leu, 71Arg)[39], vitiligo (*DRB1*-30Leu, 37Phe, 70Asp, 71Arg), and Graves’ disease[40] (*DPB1*-35Leu, 9Phe; *A*-9Phe/Tyr; *B*-45Lys, 67Phe/Tyr; *DRB1*-74Leu). In rheumatoid arthritis (RA), autoantigens are post-translationally modified, decreasing their positive charge; this resulted in reversal of risk and protection for positive and negative side-chains. Meanwhile, aromatic and large, hydrophobic side-chains appear to be risk across eleven autoimmune diseases (Table 5). In all of these studies, small, hydrophilic side-chains (*e*.*g*. Ser, Thr, Asn) in the binding groove were generally protective or neutral. Thus, similar risk and protective amino acid properties are observed, predominantly at conserved peptide-contacting positions, across autoimmune diseases and MHC subunits.

**Table 5:** Significantly associated residues across eleven autoimmune diseases within the peptide-binding groove of *HLA-DRB1*. Bold red amino-acids denote independently associated risk signals in the strongest associated *DRB1* allele, identified by the authors of each study. Bold green amino-acids denote significant protective signals. SLE: lupus, MS: multiple sclerosis, RA: rheumatoid arthritis, T1D: type 1 diabetes, HT: Hashimoto’s thyroiditis, pSS: primary Sjögren’s syndrome, SSc: systemic sclerosis, UC: ulcerative colitis, CD: Crohn’s disease, PV: pemphigus vulgaris, Vi: vitiligo. *: Complete linkage disequilibrium (D’ = 1).

Disease		Pocket														Publication (PMID)
	Residue	1	4				6			7			9			
	Allele	86	26	70	71	74	11	13	30	28	47	67	9	37	57	
Risk alleles																
SLE	DRB1*15:*01*	V	F	Q	A	A	P*	R*	Y	D	F	**I**	W	S	D	This study
MS	DRB1*15:*01*	**V**	F	Q	**A**	**A**	P*	R*	Y	D	F	I	W	S	**D**	24278027
RA	DRB1*04:*01*	G	F	**Q**	**K**	**A**	**V**	**H**	Y	D	Y	L	E	Y	**D**	25070946
T1D	DRB1*04:*05*	G	F	Q	**R**	A	V	**H**	Y	D	Y	**L**	E	Y	S	26865843
HT	DRB1*03:*01*	V	**Y**	Q	**K**	**R**	S	S	**Y**	D	F	L	E	N	D	18779568
pSS	DRB1*08:*03*	G	F	D	R	**L**	S	G	Y	D	**Y**	I	E	Y	S	25582848
SSc	DRB1*11:*04*	G	**F**	D	R	A	S	S	**Y**	**D**	F	**F**	E	Y	D	19933168
UC, CD	DRB1*01:*03*	G	L	**D**	**E**	A	L	F	C	E	Y	**I**	W	S	D	25559196
PV	DRB1*04:*02*	V	F	**D**	**E**	A	V	H	Y	D	Y	I	E	Y	D	8524878
Vi	DRB1*07:*01*	G	F	**D**	**R**	Q	G	Y	**L**	E	Y	I	W	**F**	V	21833019
Protective alleles																
SLE	DRB1*11:*01*	G	F	D	R	A	**S**	**S**	Y	D	F	F	E	Y	D	This study
MS	DRB1*14:*01*	V	F	**R**	**R**	**E**	**S**	**G**	Y	D	Y	L	E	F	**A**	24278027
RA	DRB1*14:*04*	V	F	**R**	**E**	E	**S**	**G**	Y	D	F	I	E	**N**	D	25070946
T1D	DRB1*15:*01*	V	F	Q	**A**	A	P*	R*	Y	D	F	I	W	S	D	26865843
HT	DRB1*12:*01*	V	**L**	D	**R**	A	S	G	**H**	E	F	I	E	L	V	18779568
pSS	DRB1*12:*01*	V	L	D	R	**A**	S	G	H	E	**F**	I	E	L	V	25582848
SSc	DRB1*07:*01*	**G**	**F**	D	R	Q	G	Y	L	**E**	Y	I	W	F	V	19933168
UC, CD	DRB1*03:*01*	V	Y	**Q**	**K**	**R**	S	S	Y	D	F	L	E	N	D	25559196
PV	DRB1*04:*04*	V	F	**Q**	**R**	A	V	H	Y	D	Y	L	E	Y	D	8524878
Vi	DRB1*01:*01*	G	L	**Q**	**R**	A	L	F	**C**	E	Y	L	W	**S**	D	21833019

Our study is the first to systematically associate risk of specific autoantibodies across large SLE case/control cohorts. The most-risk and most-protective residues for autoantibody association overwhelmingly map to Class II loci (particularly *DRB1* for nRNP, Ro/La, and cardiolipin and *DPB1* for Sm; Table 3), consistent with the role of CD4^+^ T-helper cells in autoantibody generation by B-cells (rather than CD8^+^ cytotoxic T-lymphocytes, which signal through T-cell receptors specific for Class I proteins).

Intriguingly, though amino acid properties were largely conserved between overall SLE and specific autoantibody risk, the positions were fairly different. *DRB1*-30 was a primary determinant of anti-nRNP risk, *DRB1*-70 of anti-Ro/La risk, and *DPB1*-11 of anti-Sm risk; none of these residues were GWS for overall SLE risk (conversely, *DRB1*-11/13 was shared between overall SLE risk and nRNP^+^, Ro/La^+^, and ACL^+^ status). These last, along with other observations (*e*.*g*. *HLA-DRB1-09*:*01* and *DRB1*-13Phe, risk for SLE and nRNP^+^, but protective for Ro/La^+^; HLA-DRB1*16:*02*, strongly risk for SLE but no detectible risk for tested autoantibodies), suggest that SLE and specific autoantibody development share some genetic risk elements but diverge at others.

Autoantibody risk mapped entirely to HLA residues contacting the middle of the peptide (p4/6 pocket, 6 of the 7 most significant residues; p7, 1/7). These recognize the C-terminal portion of the peptide, which binds to the MHC before the N-terminus[41]; p4/6-7/9 therefore serve a key role in peptide selection. Sjögren’s syndrome risk also concentrates at MHC positions recognizing p7/9[31]. Individual autoantigens present a small set of immunogenic peptides to the HLA, with autoantibody risk arising from recognition of those specific peptides. Given the enormous diversity of SLE antigens, overall SLE risk might stem from the binding of many peptides (alternatively, these antibodies might arise as a downstream consequence of pathogenesis set off by fewer peptide-autoantibody pairs).

Our data strongly suggest that these hallmark SLE autoantigens are selected in a predictable fashion by the HLA subunit (particularly *HLA-DRB1* and *HLA-DPB1*) peptide-binding grooves. Similar patterns are seen across other MHC subunits and autoimmune diseases. Negative *HLA-DPB1* p1/7/9 peptide-binding groove charge (aa-55[Asp-Glu-Glu]aa-57; aa-67[Glu-Glu-Glu]aa-68; aa-82[Glu-Asp-Glu]aa-85) is associated with risk of anti-topoisomerase antibodies in systemic sclerosis[42]; negative *HLA-DRB1* p4 pocket charge (aa-70[Asp-Glu]aa-71) is associated with anti-desmoglein antibodies in pemphigus vulgaris[35].

The development of antibodies to RNA, DNA and cardiolipin is thought to come from nucleic acid-binding protein/nucleic acid complexes breaking tolerance through recruitment of T-cell help[43]; these molecules are otherwise very poorly immunogenic. The fact that overall SLE-risk alleles and amino acids differ somewhat from those for specific autoantibodies tested here, suggests that the queried autoantibodies might result from a progression of SLE through a more general breakdown of immune processes, rather than being causal.

In summary, we identified novel SLE signals in HLA Class I loci (*HLA-A*-70, *HLA-B*-9), and localized primary Class II signals to five residues in *HLA-DRB1*, *HLA*-*DPB1*, and *HLA-DQB1*. These seven residues not only increase the proportion of HLA heritability explained to 2.6%, but also significantly increase overall risk, particularly with risk-allele accumulation. Detailed analysis expanded this to 20 risk positions across the six major HLA subunits. We demonstrate how these positions and amino-acid properties (large, aromatic, charged) correlate with peptide-MHC binding and T-cell receptor activation, for both general SLE risk residues and for risk of specific autoantibodies (nRNP, Ro/La, Sm, cardiolipin). It is of note that our study does not consider other interacting loci or individual variation in the autoantigens themselves[44], and there may be small signals remaining at other HLA loci. Our association results on Asians complement previous reports from European, African, and Hispanic populations. Importantly, our observations generalize across MHC subunits and various other autoimmune diseases. Our data and analysis present a framework for modeling peptide antigen presentation to both the MHC and the T-cell receptor, tolerance breakage, and autoantibody development.

## Materials and methods

### Ethics statement

This study was approved by the Oklahoma Medical Research Foundation’s Institutional Review Board, IRB 10–23. IRB approval was granted for use of either de-identified or coded materials collected from previous studies in which original consent included a provision for sharing; because of this, no additional informed consent was required.

### Study participants

Our study was conducted in two phases: discovery and replication. The discovery phase included primarily the participants from our previously published study (Korean, KR; Han Chinese, HC; Malaysian Chinese, MC)[8]. Details about recruitment and phenotyping for our discovery phase individuals (*n* = 10,142; 2,490 cases and 7,652 controls) can be found elsewhere[8]. To increase statistical power of our Han Chinese (HC) samples, we incorporated 392 out-of-study controls from dbGaP (phs00431.v1.p1)[45]. Our discovery set thus included 2,490 cases and 8,044 controls (S1 Table).

In order to replicate our initial findings, we included 2,425 cases and 5,469 controls from two Japanese cohorts (JP1, JP2) and one HC cohort (HC2) (S1 Table). Our first Japanese replication cohort (JP1) was collected under the support of the Autoimmune Disease Study Group of Research in Intractable Diseases, Japanese Ministry of Health, Labor and Welfare, and the BioBank Japan Project. Details about the subjects and study design are described elsewhere[46]. Samples for our second Japanese cohort (JP2) were obtained at Kyoto University, Japan. Our HC replication set (HC2) included primarily the participants recruited by UCLA, as described elsewhere[47].

All patients satisfied American College of Rheumatology criteria for SLE classification[48, 49]. Controls were geographically matched to SLE cases. Participants provided written consent at study enrollment, and the Institutional Review Boards or ethical committees of participating institutions approved this study. Potentially identifying information was removed for all participants.

### Genotyping and quality control

Genotyping and quality control of the discovery samples, as well as JP1 details, can be found in the source publications[8, 46, 50]. HC2 replication samples were genotyped on the ImmunoChip platform following the same quality-control protocol as our discovery samples. Details about the genotyping and normal HC quality controls included in the analysis are described elsewhere[50]. JP2 replication samples were genotyped on the Illumina HumanCoreExome BeadChip platform at Tokyo Medical and Dental University and Kyoto University.

### HLA imputation

To identify association at the MHC region, we extracted the SNPs within the extended MHC region (chr6: 25–35 MB) and imputed classical alleles, untyped SNPs, and amino-acid residues using SNP2HLA[51]. In order to capture the appropriate genetic background for imputation, we used two different Asian HLA imputation reference panels: a merged panel[52] made of Korean[53] and Asian[54] imputation panels was used on our Korean, Han Chinese and Malaysian samples, whereas our Japanese replication cohorts were imputed using a Japanese imputation panel[40]. All reference panels included SNPs, classical alleles, and amino acids for eight HLA loci (*HLA-A*, *-B*, *-C*, *-DPA1*, *-DPB1*, *-DQA1*, *-DQB1* and *-DRB1*; *DRA1* is practically invariant and was not genotyped in our study). Imputation accuracy using our two panels (~90%) has been described in the parent publications[40, 52]. Imputation of cases and controls was performed together for each cohort. In order to reduce the uncertainty of imputation, we restricted our analysis to variants with high imputation quality (r^2^>0.7) in each cohort. Imputed dosage results were used in all subsequent analyses.

### Association analysis

For the assessment of SLE associated signals in each cohort, we performed two types of regression analysis: logistic regression analysis for each bi-allelic marker and omnibus test (log-likelihood ratio test) for each multi-allelic marker. Each SNP, classical allele, and amino-acid residue was regressed and corrected by sex and the first three principal components (PCs). Corrected P-values after conditioning are presented throughout this paper. To estimate the association strength at amino-acid positions, we carried out omnibus tests[29, 54]. Omnibus tests for each position were set up as logistic regressions of all residues within the position (except the most frequent one), and corrected by sex and PCs. Omnibus P-values (P_omnibus_) were estimated as likelihood ratio tests of the base model A_0_ versus the expanded model A_1_:

$$A_{0}\;:\text{log}\;\left(odds\right)\;=\;\theta +\;\beta_{0}S_{i}+\;\sum_{j\;=\;1}^{3}\beta_{j}P_{i,j}+\epsilon$$

$$A_{1}\;:\text{log}\;\left(odds\right)\;=\;\theta +\;\beta_{0}S_{i}+\;\sum_{j\;=\;1}^{3}\beta_{j}P_{i,j}+\;\sum_{k\;=\;1}^{n-1}\gamma_{k}V_{i,k}+\epsilon$$

D=−2ln(LikelihoodA0LikelihoodA1)

$$D\sim {\chi^{2}}_{\left(df(A_{1})-df(A_{0})\right)},$$

where the *θ* term is the intercept. *β* and *γ* are the logistic regression coefficients, *P*_*i*,*j*_ is the *j*-th principal component of individual *i*, *S*_*i*_ is the sex of individual *i* recoded as a binary categorical variable (male = 0, female = 1), *V*_*i*,*k*_ is the dosage of the *k*^th^ amino-acid residue in individual *i* among the *n* possible side-chains (ordered from *k* = 1, least frequent, to *k* = *n*, most frequent), *ϵ* is an error term, *df* is the degrees of freedom, and *D* is the omnibus statistic.

All conditional analyses were performed as omnibus association tests and were nested into two stages, A: conditioning for the strongest residue, and B: conditioning for the full effect of the corresponding alleles (S1 Fig). Conditional analyses were cumulative, where each step includes the condition for the full effect of the genes in the previous steps. We used the significance threshold 5x10^-5^ for all omnibus tests. The complete analysis consisted of six steps as described below.

In the first step of the analysis, we conducted an unconditional omnibus test of all SNPs, residues and alleles, and used this as the basis to identify the strongest signal (S1 and S2a Figs). In the second step, we fine-mapped the residues within *HLA-DRB1* (A, S2b and S2c Fig), and conditioned for the effect of all *DRB1* alleles (B; S2d Fig).

In step 3, we conditioned for residue *A-7*0 (S2e Fig), and for the full effect of *HLA-DRB1* and *HLA-A* (S2f Fig). In step 4, we conditioned for residue *DPB1-35* (S2g Fig), and for the full effect of *HLA-DRB1*, *HLA-A* and *HLA-DPB1* (S2h Fig). In step 5, we conditioned for residue *DQB1-37* (S2i Fig), and for the effect of *HLA-DRB1*, *HLA-A*, *HLA-DPB1* and *HLA-DQB1* (S2j Fig). In the final step, we conditioned for residue *B-9* (S2k Fig), and for the full effect of *HLA-DRB1*, *HLA-A*, *HLA-DPB1*, *HLA-DQB1* and *HLA-B* alleles (S2l Fig).

### Combined P-values

In order to combine cohort-specific association P-values while preserving effect size and direction, data for individual SNPs, amino acids, and classical alleles were combined through sample size-corrected meta-analysis, as implemented in Metal[55]. Odds ratios for the combined P-values were estimated using the standard error approach. This method was ultimately used because only summary statistics for the Japanese cohorts were available to us. To combine P-values from omnibus tests, we used Fisher’s method, which is recommended when effect sizes are not available[56, 57].

### Explained proportion of heritability

In order to estimate the proportion of explained heritability contributed by each of our independent residues (amino-acid positions), we used the liability model described by So and Sham[58], which estimates the effect of risk alleles on genetic liability. In this case, we used the odds ratios of all risk amino acids within a residue. *HLA-DRB1* residue 11 (henceforth referred to as *DRB1-11*) was removed from the estimation of the total effect on liability because *DRB1-13* is tightly linked to it; we included only *DRB1*-13 in this total calculation to avoid inflation.

### Accumulation of risk residues

In order to assess the effect of accumulating risk/protective alleles (for this case, allele is meant for residue), we used the best-guess (phased) genotypes for our discovery and replication sets. For each individual, we counted the number of risk and protective amino acids present for all seven independent residues (*A*-70; *B*-9; *DPB1*-35; *DQB1*-37; *DRB1*-11/13, and either 37 or correlated residue 26). We estimated the odds ratios of having 1 to >8 risk residues versus none. Individuals whose best-guess genotype was uncertain were removed from this analysis. We regressed the number of risk alleles versus odds ratio and identified the best fitting model. In order to assess additivity of the effects, we performed logistic regression for all combination of single and multiplicative effects (modeled as interactions). Additive models were compared to interaction models through the Akaike information criterion. In order to investigate if the effect of the accumulation of risk amino-acids weighted by their odds ratios was a better predictor of SLE risk *versus* the unweighted count of risk amino-acids, we estimated a genetic risk score for each imputed haplotype, and estimated the area under the curve. Comparison of the weighted versus unweighted ROC curves was performed using the Bonferroni test[59] in easyROC.

### Long-range HLA haplotype analysis

To estimate the combined effect on SLE susceptibility of inheriting independent residues, we used the best-guess amino-acid haplotypes for all independent residues (*HLA-A*-70; *HLA-B*-9; *HLA-DPB1*-35; *HLA-DQB1*-37; *HLA-DRB1*-11/13, 37) and constructed all haplotype combinations of *A*, *B*, *DPB1* and *DQB1* with *DRB1*-11/13-26 or *DRB1*-11/13-37. To investigate interactions between *DPA1/DPB1* residues, we selected all significant *DPB1* and *DPA1* residues and constructed haplotype combinations. Each constructed haplotype was regressed against case/control status using R.

To estimate the most common protective and risk haplotype between HLA alleles, we used the best-guess genotypes. Haplotype construction was performed in the haplo/stats library in R through the expectation-minimization algorithm. Analysis of the haplotypes derived from the phased imputed data yielded similar results. Odds ratios for each haplotype were estimated through a generalized linear model. Linkage disequilibrium between pairs of alleles was estimated as specified by Lewontin[60].

### Expression analysis

To investigate the effect of the imputed alleles, residue amino-acids and SNPs on HLA gene expression, we imputed 373 European individuals from the 1000Genomes Project using the T1DGC European reference panel[51] using SNP2HLA. We extracted RNA-seq expression data for those same individuals from the GEUVADIS project[61], and estimated the linear model for each SNP, residue and allele (with r2>0.8). We assessed gene expression of each gene conditioned on the effect of each of our identified independent residues.

### Protein structural representations

Structural representations of *HLA-A*, *HLA-B*, *HLA-C*, *HLA-DPA1/DPB1*, *HLA-DQA1/DQB1*, and *HLA-DRA1/DRB1* were produced (PyMOL), using PBD files 4HWZ, 3VCL, 1IM9, 3LQZ, 1JK8, and 2SEB, respectively. For display of overall SLE risk across each protein, appropriately conditioned -log(P_omnibus_) was linearly normalized to the interval [0, 1], with the least-associated position mapping to 0 and the most-associated position mapping to 1. Then each normalized value was converted to the RGB color (x, 0, 1 –x). Thus, the most highly associated position is shown as deep red (1, 0, 0), and the most weakly-associated position as deep blue (0, 0, 1). Intermediate values map linearly (according to –logP) between blue and red. This creates a simple visualization of the 3-dimensional distribution of risk across each HLA subunit, with concentrations of red positions highlighting the regions of strongest association. Significantly associated positions are indicated by text labels.

### Autoantigens in SLE and rheumatoid arthritis (RA)

Based on literature review, protein sequences were collected from NCBI for principal autoantigens of both SLE and RA. For SLE, the four protein autoantigens that were experimentally characterized in this study (nRNP, Ro, La, and Sm), as well as histones H1 and H2B[62], were catalogued. Multiple protein subunits were studied for two proteins: nRNP (U70, U1A, C) and Sm (B, B’, N, D1, D2, D3, E, F, G). For RA, the key autoantigens fibrinogen (fibrin precursor; A and B subunits), vinculin, collagen type II, filaggrin, vimentin, and keratin were catalogued.

For each of these proteins, total charge and isoelectric point (pI) were computed (DNASTAR 14.0.0 EditSeq). The RA autoantigens were also modeled in their poly-citrullinated forms, and the charge and pI were recalculated. For sake of these calculations, citrulline was approximated by glutamine, the naturally occurring amino acid to which it is most similar. Total charge was then normalized to a per-residue charge by dividing by the length of the protein.

### Statistical model of HLA-epitope interaction

In order to generalize the binding of MHC subunits to arbitrary peptides, we used a statistical potential[63], which represents the favorability of specific amino acids contacting one another, derived from an analysis of solved structures in the Protein Data Bank (**Web Resources**). Given the extraordinary diversity of antigens in SLE, this was deemed a practical way to address the numerous potential peptide/MHC combinations, rather than considering specific peptide/allele pairs with an atomistic (*e*.*g*. molecular mechanical) description of the interaction. This also has the simplifying assumption that MHC risk is additive across binding-groove residues (when in reality protein side-chains interact both structurally, through atomic interactions with each other and with the peptide, and genetically, in that side-chain combinations are linked together in alleles). To create a simple MHC allele “immunogenic peptide preference” score, amino acids statistically over- and under-represented in peptides initiating T-cell activation by the interaction between peptide/MHC and T-cell receptor (TCR) were taken from Calis *et al*.[27]. The statistical potential gives a score to each possible MHC side-chain interacting with each possible peptide amino acid. For each possible MHC side-chain, the metric computes the difference between the statistical potential values for antigenic-over-represented amino acids and antigenic-under-represented ones. In this way, a ranked list of MHC side-chains at positions in the peptide-binding groove (where the side-chains could contact the peptide) that would be predicted to favor peptide recognition and receptor activation was created (S1 Text).

Because RA autoantigens were quite negatively charged, and SLE autoantigens quite positively charged, we made a simple change to the statistical model for the two different autoimmune diseases: for RA, the Calis *et al*.[27] dataset was used as is; whereas for SLE, Glu (negatively charged) was replaced by Arg (positively charged) in the list of immunogenic peptide residues, and Lys (positively charged) was removed from the list of non-immunogenic peptide residues (Table 6 differences ***bold italic***):

**Table 6:** Rheumatoid arthritis and systemic lupus erythematosus immunogenic aminoacids.

Disease	Immunogenic aa’s	Non-immunogenic aa’s
RA	Trp, Phe, Ile, ***Glu***	Ser, Met, Gln, ***Lys***
SLE	Trp, Phe, Ile, ***Arg***	Ser, Met, Gln

As an example of how calculations were performed, Trp is enriched in immunogenic peptides[27] (in both our SLE and RA models), and Pro has a strongly favorable interaction with Trp in the statistical potential[63] (S10 Table); thus a Pro at an MHC binding-groove position might be expected to interact with Trp-bearing peptides in such a way as to contribute to an immune response (given that the enriched residues are taken from T-cell activation studies, this convolves both MHC-peptide and MHC-peptide-TCR interactions). The “preference” of a potential MHC binding-groove side-chain for immunogenic peptides was computed as the difference in the interactions with the immunogenic and non-immunogenic amino acids. For instance, an MHC binding-groove Phe is estimated to strongly prefer immunogenic peptides, based on the strength of its favorable interactions with immunogenic Trp/Phe/Ile and on unfavorable interactions with non-immunogenic Ser/Met/Gln (S10 Table). Similarly, Thr is estimated to strongly prefer non-immunogenic peptides, based on the strength of its exact opposite preference (*i*.*e*. unfavorable interactions with immunogenic Trp/Phe/Ile and on favorable interactions with non-immunogenic Ser/Met/Gln).

### Autoantibody associations

We performed sub-phenotype association analysis with six autoantibody profiles (antibodies against nuclear ribonuclear protein, nRNP; Ro/SSA; La/SSB; Smith, Sm; double-stranded DNA, dsDNA; and cardiolipin phospholipid, ACL). nRNP, Ro, La and Sm are binding proteins for negatively-charged nucleic acids[64]. Cardiolipin is a negatively-charged phospholipid. Autoantibodies develop first against the positively-charged nucleic acid-binding proteins, and through tolerance breakage, antibodies subsequently develop against their ligands (ssRNA and ssDNA) and mimetics such as dsDNA and cardiolipin. All analyses were carried out using logistic regression and omnibus tests as described above for the case/control analysis. Data for case-only analysis was available only for the Korean cohort and one Han Chinese cohort (S11 Table).

For each of the autoantibody profiles, the residue positions with significant omnibus association P-values (P_omnibus_<5x10^-8^; S12 Table) were selected for further study. Within this list, the most statistically significant side-chain associations were tabulated and evaluated according to the statistical model above.

## Supporting information

S1 Fig Framework for conditional analysis steps taken in this manuscript. Residues conditioned on are presented in green.
(PPTX)

S2 Fig Unconditioned and conditional analysis of HLA alleles and residues. Genomic position coordinates are presented as megabases for the hg19 genomic build. SNPs are presented in black, residues (AA) are presented in red, and alleles in blue. Lines for genome-wide association (P<5x10^-8^) in red, and for suggestive association (P<5x10^-5^) in green. **A. Unconditioned omnibus analysis. Conditional analysis. B. Conditioning for the effect of *DBR1 11+DRB1-*13 residues. C. Conditioning for the effect of *DBR1 11+DRB1-13+ DRB1-37* residues. D. Conditioning for the effect of *DBR1* alleles. E. Conditioning for the effect of *DBR1* alleles + *A*-70 residue. F. Conditioning for the effect of *DBR1 +A* alleles. G. Conditioning for the effect of *DBR1 +A* alleles + *DPB1*-35 residue. H. Conditioning for the effect of *DBR1 +A +DPB1* alleles. I. Conditioning for the effect of *DBR1 +A +DPB1* alleles + *DQB1*-37. J. Conditioning for the effect of *DBR1 +A +DPB1 +DQB1* alleles. K. Conditioning for the effect of *DBR1 +A +DPB1 +DQB1* alleles + *B*-9. L. Conditioning for the effect of *DBR1 +A +DPB1 +DQB1 +B* alleles.**
(PPTX)

S3 Fig Effect of the accumulation of risk amino acid residues. Trend for the increasing risk with increasing number of risk alleles was observed in both discovery and replication cohorts. There was a linear relationship between increased ORs and number of risk residues (R^2^ = 0.95; R^2^ = 0.90 for discovery and replication respectively), suggesting an additive effect.
(PPTX)

S4 Fig Effect of the alleles and residues on *HLA-DRB1* expression. A. Unconditioned HLA-DRB1 expression. B. *HLA-DRB1* expression conditioned on residues *DRB1-11+13+37*. C. *HLA-DRB1* expression conditioned on 4-digit *HLA-DRB1* classical alleles. Genomic position coordinates are presented as megabases for the hg19 genomic build. SNPs are presented in black, residues (AA) are presented in red, and alleles in blue. Lines for genome-wide association (P<5x10^-8^) in red, and for suggestive association (P<5x10^-5^) in green.
(PPTX)

S5 Fig Risk residues for Class I HLA genes. Accumulation of Class I risk residues in the peptide-binding groove is color-coded according to the strength of association (from blue to red). **A.** Accumulation of Class I risk residues in the peptide-binding groove; B. Accumulation of Class I risk residues in the peptide-binding groove (zoom); C. Risk residues in the peptide-binding groove of *HLA-A*.; D. Risk residues in the peptide-binding groove of *HLA-A* (zoom); E. Risk residues in the peptide-binding groove of *HLA-A*. Post-conditioning on *DRB1*-11/13, *DRB1-37, DQB1-37, A-70, DPB1-35*, and *B-9*; F. Risk residues in the peptide-binding groove of *HLA-B*; G. Risk residues in the peptide-binding groove of *HLA-B* (zoom); H. Risk residues in the peptide-binding groove of *HLA-B*. Post-conditioning on *DRB1*-11/13, *DRB1*-37, DQB1-37, A-70, DPB1-35, and B-9; I. Risk residues in the peptide-binding groove of HLA-B. Post-conditioning on *DRB1-11/13, DRB1-37, DQB1-37, A-70, DPB1-35, and B-9* (zoom). *B*-63 emerges as a significantly associated residue in addition to *B*-9; J. Risk residues in the peptide-binding groove of *HLA-C*; K. Risk residues in the peptide-binding groove of *HLA-C* (zoom); L. Risk residues in the peptide-binding groove of *HLA-C*. Post-conditioning on *DRB1-11/13, DRB1-37, DQB1-37, A-70, DPB1-35, and B-9*.
(PPTX)

S6 Fig Risk residues for Class II HLA genes. A. Accumulation of Class II risk residues in the peptide-binding groove; B. Accumulation of Class II risk residues in the peptide-binding groove (zoom); C Risk residues in the peptide-binding groove of *HLA-DPA1/DPB1*. Post-conditioning on *DRB1-11/13, DRB1-37* and *DQB1-37*; D. Risk residues in the peptide-binding groove of *HLA-DPA1/DPB1*. Post-conditioning on *DRB1-11/13, DRB1-37 and DQB1-37* (zoom). *DPB1-55* emerges as a significantly associated residue in addition to *DPB1-35*; E. Risk residues in the peptide-binding groove of *HLA-DPA1/DPB1*. Post-conditioning on *DRB1-11/13, DRB1-37, DQB1-37, A-70, DPB1-35, and B-9*; F. Risk residues in the peptide-binding groove of *HLA-DPA1/DPB1*. Post-conditioning on *DRB1*-11/13, *DRB1*-37, *DQB1*-37, *A*-70, *DPB1*-35, and *B*-9 (zoom). *DPA1*-11 emerges as a significantly associated residue in addition to *DPB1*-35; G. Risk residues in the peptide-binding groove of *HLA-DQA1/DQB1*. Post-conditioning on *DRB1*-11/13 and *DRB1*-37; H. Risk residues in the peptide-binding groove of *HLA-DQA1/DQB1*. Post-conditioning on *DRB1*-11/13 and *DRB1*-37 (zoom). *DQB1*-70 emerges as a significantly associated residue in addition to *DQB1*-37; I. Risk residues in the peptide-binding groove of *HLA-DQA1/DQB1*. Post-conditioning on *DRB1*-11/13, *DRB1*-37, *DQB1*-37, *A*-70, *DPB1*-35, and *B*-9; J. Risk residues in the peptide-binding groove of *HLA-DQA1/DQB1*. Post-conditioning on *DRB1*-11/13, *DRB1*-37, *DQB1*-37, *A*-70, *DPB1*-35, and *B*-9 (zoom). *DQB1*-57 emerges as a significantly associated residue in addition to *DQB1*-37; K. Risk residues in the peptide-binding groove of *HLA-DRA1/DRB1*; L. Risk residues in the peptide-binding groove of *HLA-DRA1/DRB1* (zoom); M. Risk residues in the peptide-binding groove of *HLA-DRA1/DRB1*. Post-conditioning on *DRB1*-11/13; N. Risk residues in the peptide-binding groove of *HLA-DRA1/DRB1*. Post-conditioning on *DRB1*-11/13, *DRB1*-37, *DQB1*-37, *A*-70, *DPB1*-35, and *B*-9. *DRB1*-67 emerges as a significantly associated residue; O. Correspondence of risk positions across multiple Class I (left) and Class II (right) subunits. Risk position 35 in *DQB1* and *DRB1* is equivalent to *DPB1*-35 due to a 2-amino acid deletion; similarly *DQB1*-57 is equivalent to *DPB1*-55. Position 152 in *A* and *C* is equivalent to *DRB1*-67. *C*-99 and *A*-97 are equivalent to *DRB1*-13 and *DRB1*-11, respectively. *A*-114 is equivalent to *DRB1*-26. Position 9 appears as a top risk position for both *B* and *C*.
(PPTX)

S1 Table Samples used in this study. (XLSX)

S2 Table HLA allele association in all cohorts. Logistic regression results for all classical alleles across all cohorts, and meta-analysis. FA/FU: Case/control frequencies; OR, LCI, UCI: Odds ratio, 95% confidence interval.
(XLSX)

S3 Table SNP association among all cohorts. Logistic regression results for all SNPs across all cohorts, and meta-analysis. FA/FU: Case/control frequencies; OR, LCI, UCI: Odds ratio, 95% confidence interval.
(XLSX)

S4 Table Amino acid association in all cohorts. Logistic regression results for all amino acids across all cohorts, and meta-analysis. FA/FU: Case/control frequencies; OR, LCI, UCI: Odds ratio, 95% confidence interval.
(XLSX)

S5 Table Omnibus residue association tests and conditional analysis. Omnibus association for the discovery phase (P_Discovery), followed by each step of conditional analysis marked by gene (R, Q, P, A, B, for *DRB1*, *DQB1*, *DPB1*, *A*, and *B* respectively) and the residues conditioned on. Selected independent residues are highlighted in green.
(XLSX)

S6 Table Heritability of independent residue amino acids. Risk amino-acids contribute to heritability due to HLA. Coding of each amino-acid is based on presence of a particular amino-acid versus presence of any other amino-acid at that residue, and thus only risk amino-acids are used for their contribution to heritability. ROR: risk Odds Ratio; FRQ: frequency; RAF: risk allele frequency; Vg: contribution to genetic liability. Proportion of heritability was estimated as a percentage for the discovery cohorts as a whole (%KHM), and as separate (%KR, HC, MC).
(XLSX)

S7 Table Accumulation of risk alleles. Analysis of the accumulation of risk residues for both discovery and replication are presented for the model with previously reported *DRB1*-26, and with our *DRB1*-37 as replacement.
(XLSX)

S8 Table Haplotype analysis of HLA alleles. A. HLA-Allele haplotype analysis. Pairs of alleles of *DRB1*+*DQB1* were tested for the effect of inheriting a particular combination. B. Risk residue haplotype analysis. FR: frequency; D'/r2, linkage disequilibrium for each pair of alleles.
(XLSX)

S9 Table Primary autoantigens for SLE (a) and RA (b). Shown are protein sequence, isoelectric point (pI), length, # and fraction of residues that are Arg (R), Lys (K), Asp (D) and Glu (E), charge, charge per residue, and pI and charge per residue following poly-citrullination. For sake of easily modeling charge and pI, citrulline was approximated by glutamine. Red colors represent basic (positively-charged) proteins. Blue colors represent acidic (negatively-charged) proteins.
(XLSX)

S10 Table Simple model of MHC side-chains interacting with (S10 A) SLE and (S10 B) RA peptide antigen side-chains. **A.** As SLE autoantigens are frequently highly positively-charged, Arg was added to and Glu omitted from the list of "over-represented in antigenic peptides list". Similarly, Lys was omitted from the "under-represented in antigenic peptides" list. Preference for antigenic versus non-antigenic side-chains was calculated both by the difference in SUM and the MIN of RIFW versus QMS. The MHC side-chains found to most prefer SLE-immunogenic peptide side-chains include Trp, Ile, Phe, Leu, Glu, Tyr, Pro, Met and Asp. Those found to least prefer SLE antigenic side-chains include Thr, Gly, Ser, Cys, Asn, Lys, Gln, His and Arg. Ala and Val were intermediate. **B.** The list of immunogenic side-chains was left unchanged from Calis *et al*. Preference for antigenic versus non-antigenic side-chains was calculated both by the difference in SUM and the MIN of EIFW versus KQMS. The MHC side-chains found to most prefer RA citrullinated immunogenic peptide side-chains include Lys, Arg, Ile, Leu, Phe, Ala, Met, Val and Pro. Those found to least prefer RA citrullinated peptide antigenic side-chains include Asp, Glu, Gly, Gln, Asn, Tyr and Thr. Cys, Ser, His and Trp were intermediate. Amino acid pairwise interaction scores taken from Simons, K.T. *et al*. Proteins 34, 82–95 (1999). Over- and under-represented side-chains are taken from Calis, J.J. *et al*. PLoS Comput Biol 9, (2013). Abbreviations: Ala (A), Arg (R), Asn (N), Asp (D), Cys (C), Gln (Q), Glu (E), Gly (G), His (H), Ile (I), Leu (L), Lys (K), Met (M), Phe (F), Pro (P), Ser (S), Thr (T), Trp (W), Tyr (Y), Val (V).
(XLSX)

S11 Table Autoantibody samples count. Counts of nRNP and Ro/La presence and absence in SLE cases for the Korean and Han Chinese discovery cohorts.
(XLSX)

S12 Table Autoantibody association for nRNP+/Ro/La+ versus nRNP-/Ro/La-. Genome-wide significant residues and amino-acids for the contrast between presence and absence of autoantibodies (nRNP and Ro/La) are presented as omnibus test (P_omnibus), and logistic regression (P_log). Ab = Autoantibody; pos_n(freq)/neg_n(freq): number of cases (proportion of cases) positive/negative for presence of autoantibody; df: degrees of freedom; OR, hi_ci, low_ci: odds ratios and 95% high and low confidence limits respectively.
(XLSX)

S1 Text Modeling MHC-peptide-T-cell receptor interactions. (DOCX)
