# Supplementary material for: Amino acid signatures of HLA Class-I and II molecules are strongly associated with SLE susceptibility and autoantibody production in Eastern Asians
Source: PLoS Genet. 2019 Apr 25;15(4):e1008092. doi: 10.1371/journal.pgen.1008092 (PMC6504188; doi:10.1371/journal.pgen.1008092)
Supplement: S1 Fig — Residues conditioned on are presented in green. (PPTX) [file pgen.1008092.s001.pptx]

## Slide 1
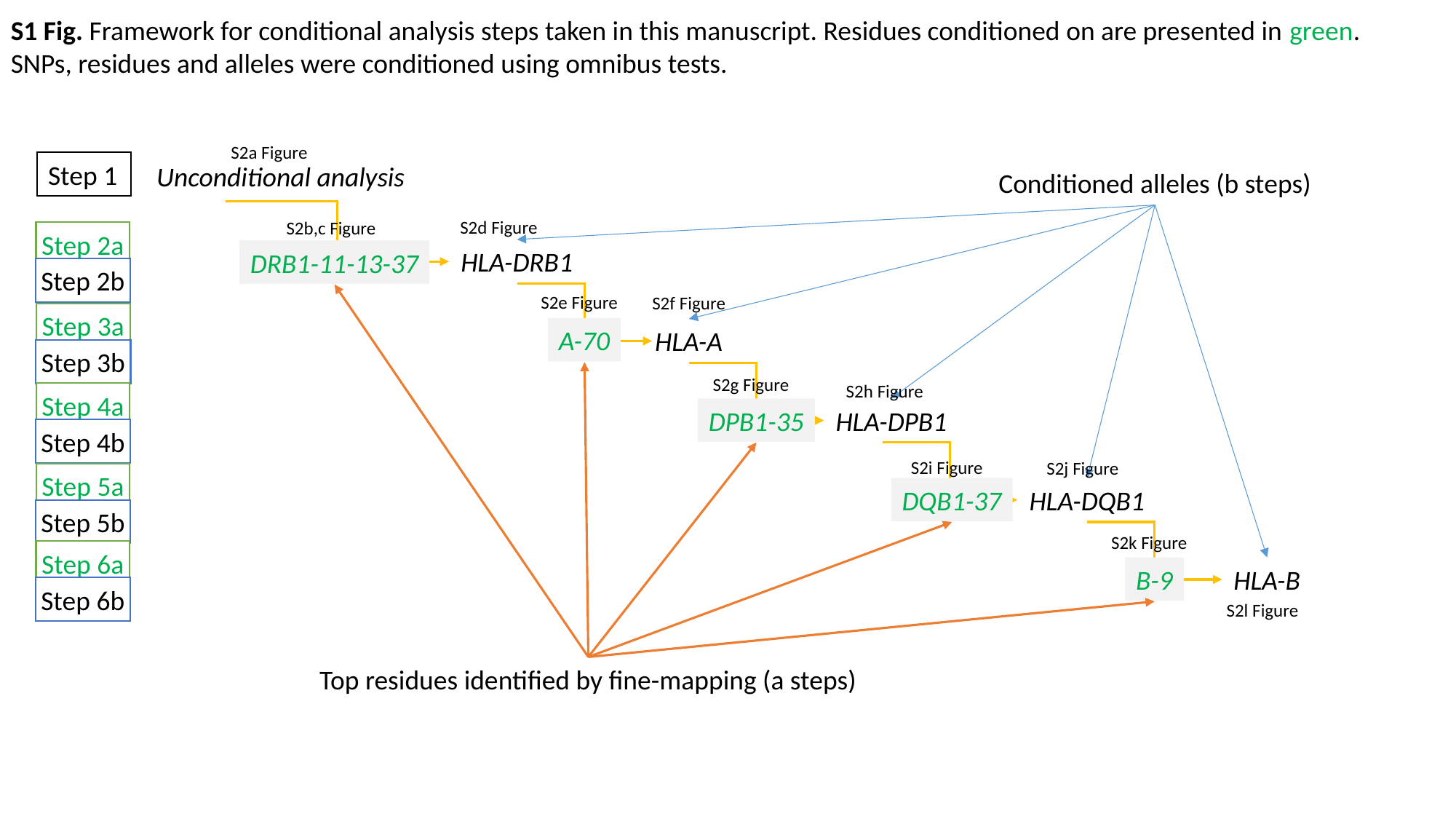

S1 Fig. Framework for conditional analysis steps taken in this manuscript. Residues conditioned on are presented in green. SNPs, residues and alleles were conditioned using omnibus tests.
S2a Figure
Step 1
Unconditional analysis
HLA-DRB1
A-70
HLA-A
DPB1-35
HLA-DPB1
DQB1-37
HLA-DQB1
B-9
HLA-B
DRB1-11-13-37
Conditioned alleles (b steps)
Top residues identified by fine-mapping (a steps)
Step 2a
Step 2b
Step 3a
Step 3b
Step 4a
Step 4b
Step 5a
Step 5b
Step 6a
Step 6b
S2d Figure
S2b,c Figure
S2e Figure
S2f Figure
S2g Figure
S2h Figure
S2i Figure
S2j Figure
S2k Figure
S2l Figure
