# Supplementary material for: Amino acid signatures of HLA Class-I and II molecules are strongly associated with SLE susceptibility and autoantibody production in Eastern Asians
Source: PLoS Genet. 2019 Apr 25;15(4):e1008092. doi: 10.1371/journal.pgen.1008092 (PMC6504188; doi:10.1371/journal.pgen.1008092)
Supplement: S2 Fig — Genomic position coordinates are presented as megabases for the hg19 genomic build. SNPs are presented in black, residues (AA) are presented in red, and alleles in blue. Lines for genome-wide association (P<5x10^-8^) in red, and for suggestive association (P<5x10^-5^) in green. A. Unconditioned omnibus analysis. Conditional analysis. B. Conditioning for the effect of DBR1 11+DRB1-13 residues. C. Conditioning for the effect of DBR1 11+DRB1-13+ DRB1-37 residues. D. Conditioning for the effect of DBR1 alleles. E. Conditioning for the effect of DBR1 alleles + A-70 residue. F. Conditioning for the effect of DBR1 +A alleles. G. Conditioning for the effect of DBR1 +A alleles + DPB1-35 residue. H. Conditioning for the effect of DBR1 +A +DPB1 alleles. I. Conditioning for the effect of DBR1 +A +DPB1 alleles + DQB1-37. J. Conditioning for the effect of DBR1 +A +DPB1 +DQB1 alleles. K. Conditioning for the effect of DBR1 +A +DPB1 +DQB1 alleles + B-9. L. Conditioning for the effect of DBR1 +A +DPB1 +DQB1 +B alleles. (PPTX) [file pgen.1008092.s002.pptx]

## Slide 1
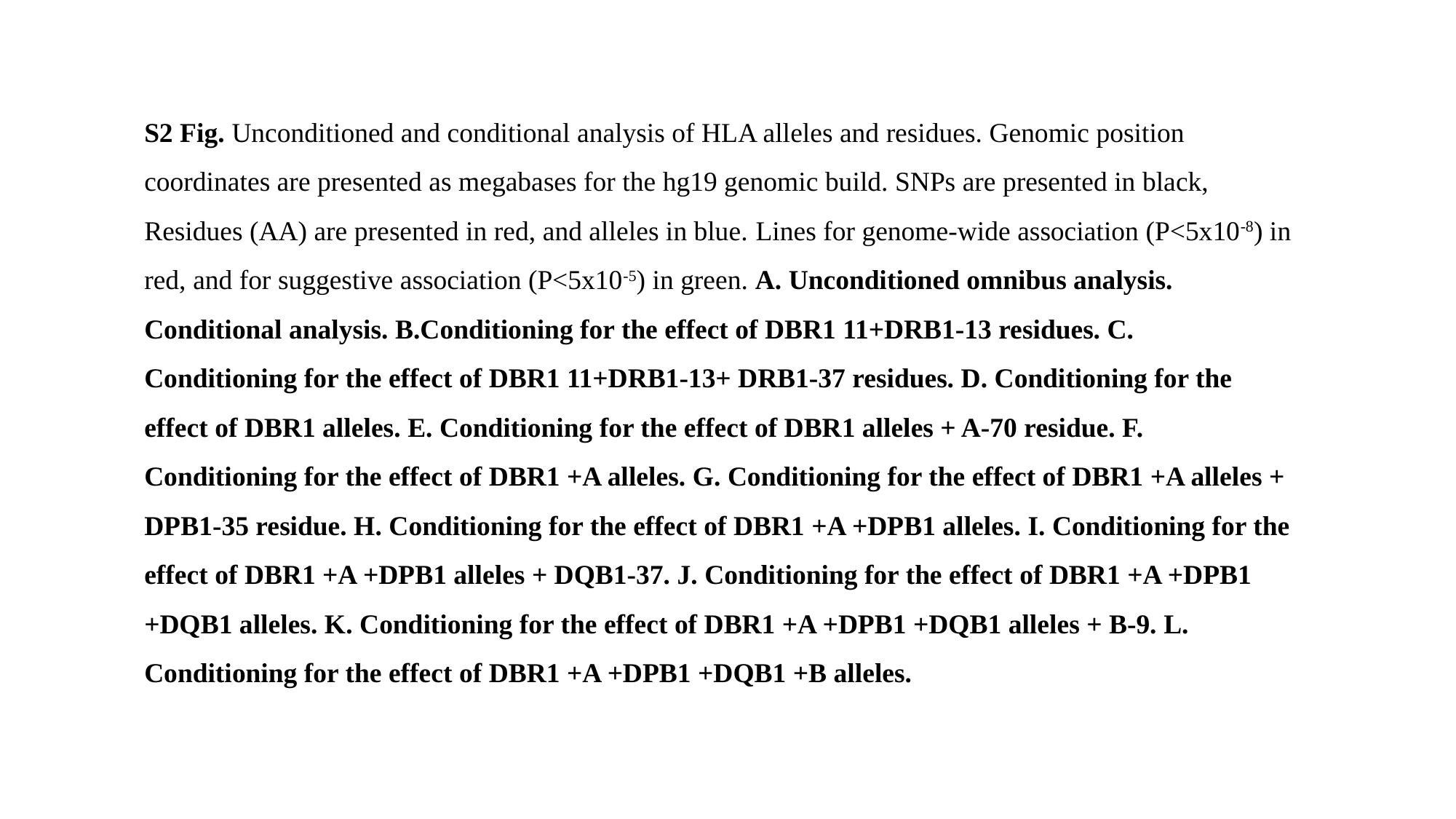

S2 Fig. Unconditioned and conditional analysis of HLA alleles and residues. Genomic position coordinates are presented as megabases for the hg19 genomic build. SNPs are presented in black, Residues (AA) are presented in red, and alleles in blue. Lines for genome-wide association (P<5x10^-8^) in red, and for suggestive association (P<5x10^-5^) in green. A. Unconditioned omnibus analysis. Conditional analysis. B.Conditioning for the effect of DBR1 11+DRB1-13 residues. C. Conditioning for the effect of DBR1 11+DRB1-13+ DRB1-37 residues. D. Conditioning for the effect of DBR1 alleles. E. Conditioning for the effect of DBR1 alleles + A-70 residue. F. Conditioning for the effect of DBR1 +A alleles. G. Conditioning for the effect of DBR1 +A alleles + DPB1-35 residue. H. Conditioning for the effect of DBR1 +A +DPB1 alleles. I. Conditioning for the effect of DBR1 +A +DPB1 alleles + DQB1-37. J. Conditioning for the effect of DBR1 +A +DPB1 +DQB1 alleles. K. Conditioning for the effect of DBR1 +A +DPB1 +DQB1 alleles + B-9. L. Conditioning for the effect of DBR1 +A +DPB1 +DQB1 +B alleles.

## Slide 2
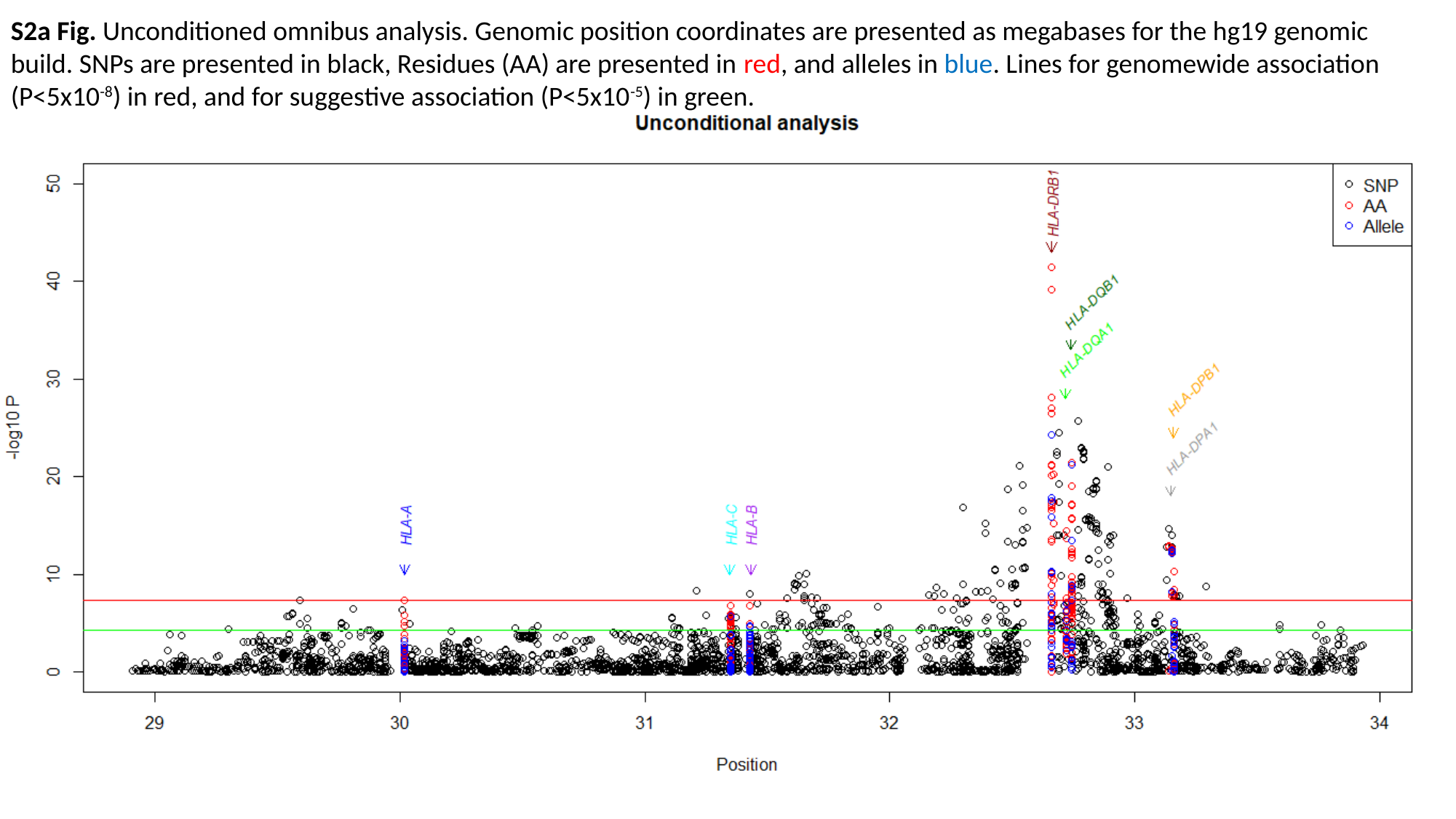

S2a Fig. Unconditioned omnibus analysis. Genomic position coordinates are presented as megabases for the hg19 genomic build. SNPs are presented in black, Residues (AA) are presented in red, and alleles in blue. Lines for genomewide association (P<5x10^-8^) in red, and for suggestive association (P<5x10^-5^) in green.

## Slide 3
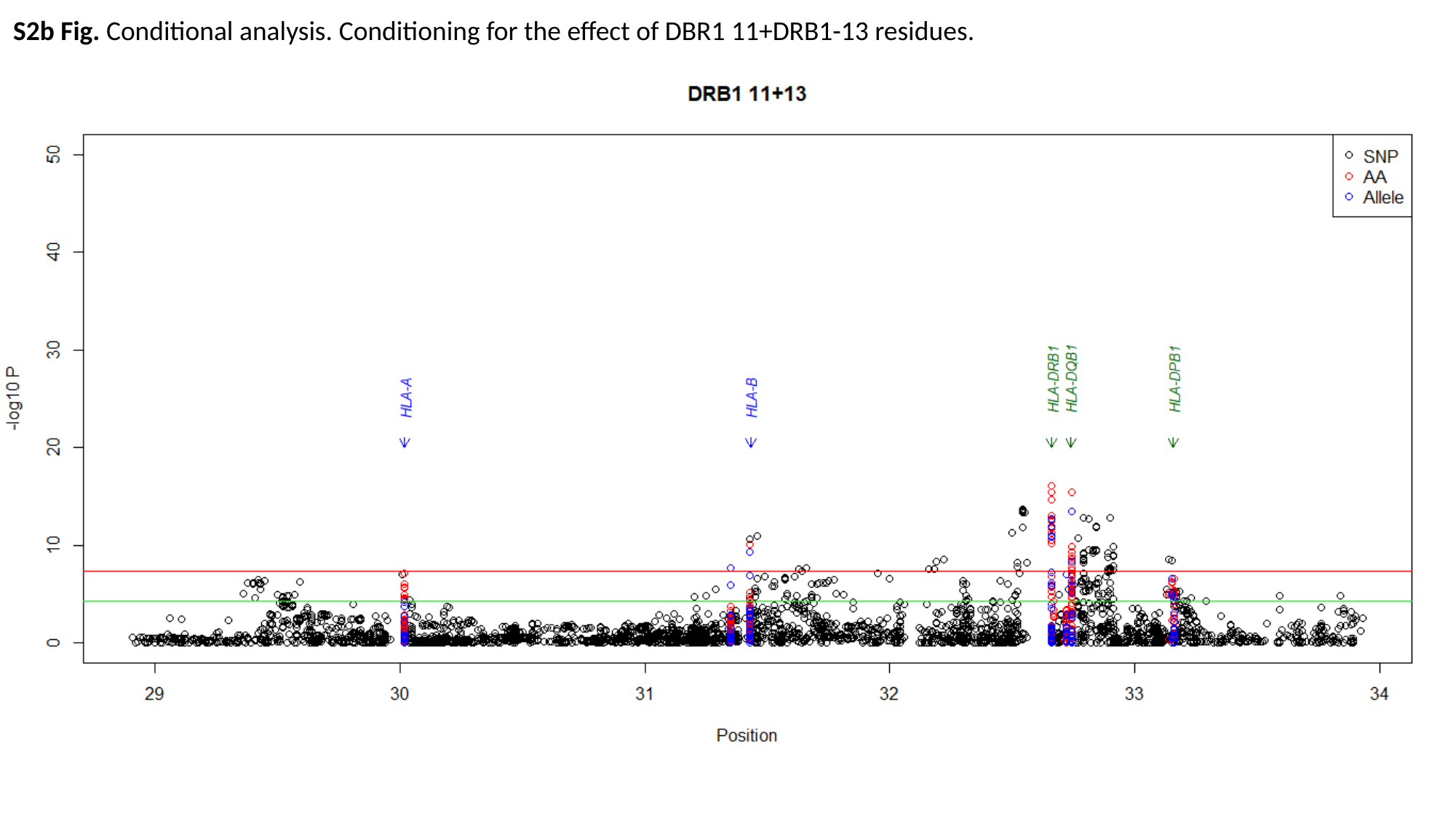

S2b Fig. Conditional analysis. Conditioning for the effect of DBR1 11+DRB1-13 residues.

## Slide 4
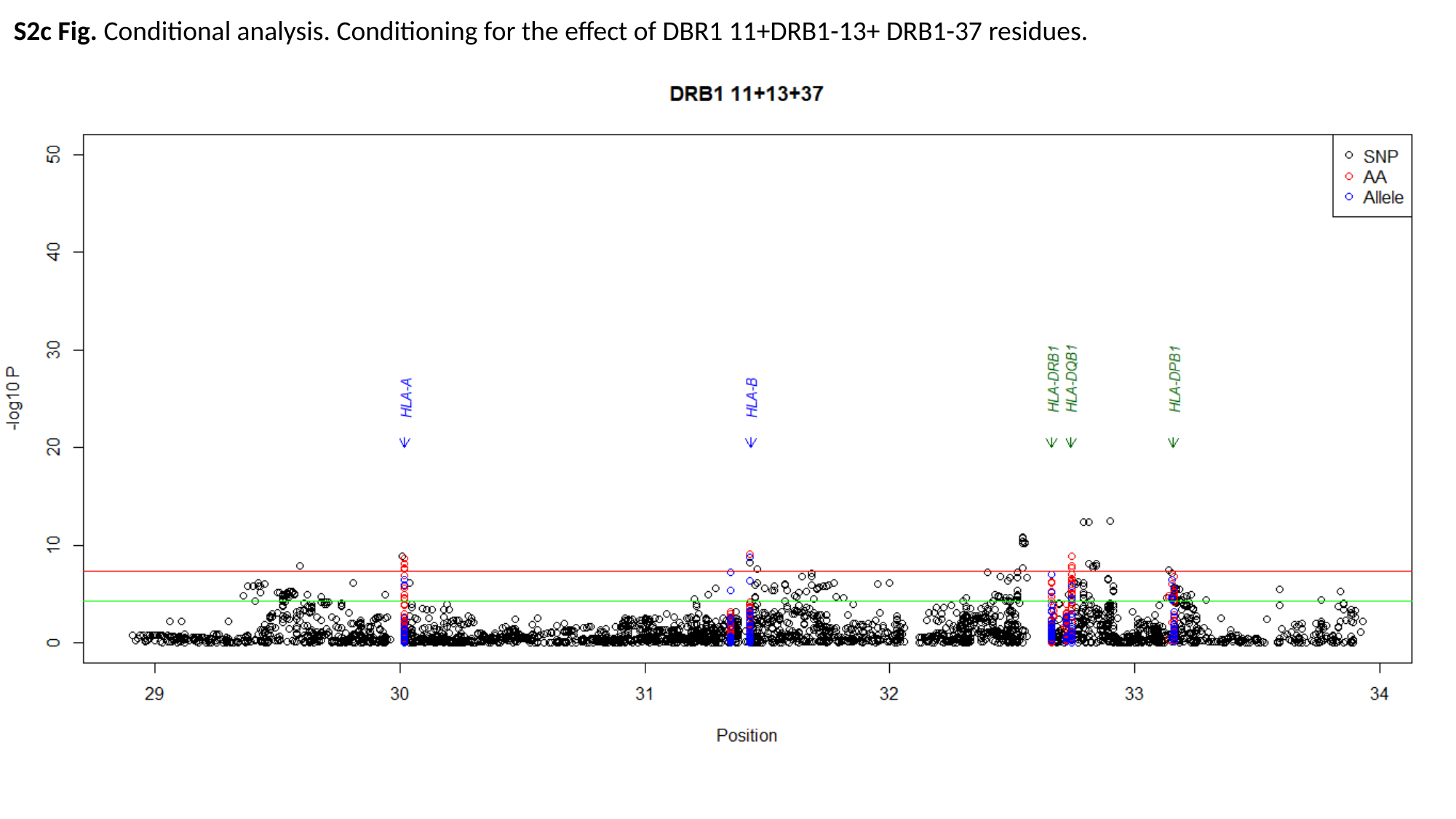

S2c Fig. Conditional analysis. Conditioning for the effect of DBR1 11+DRB1-13+ DRB1-37 residues.

## Slide 5
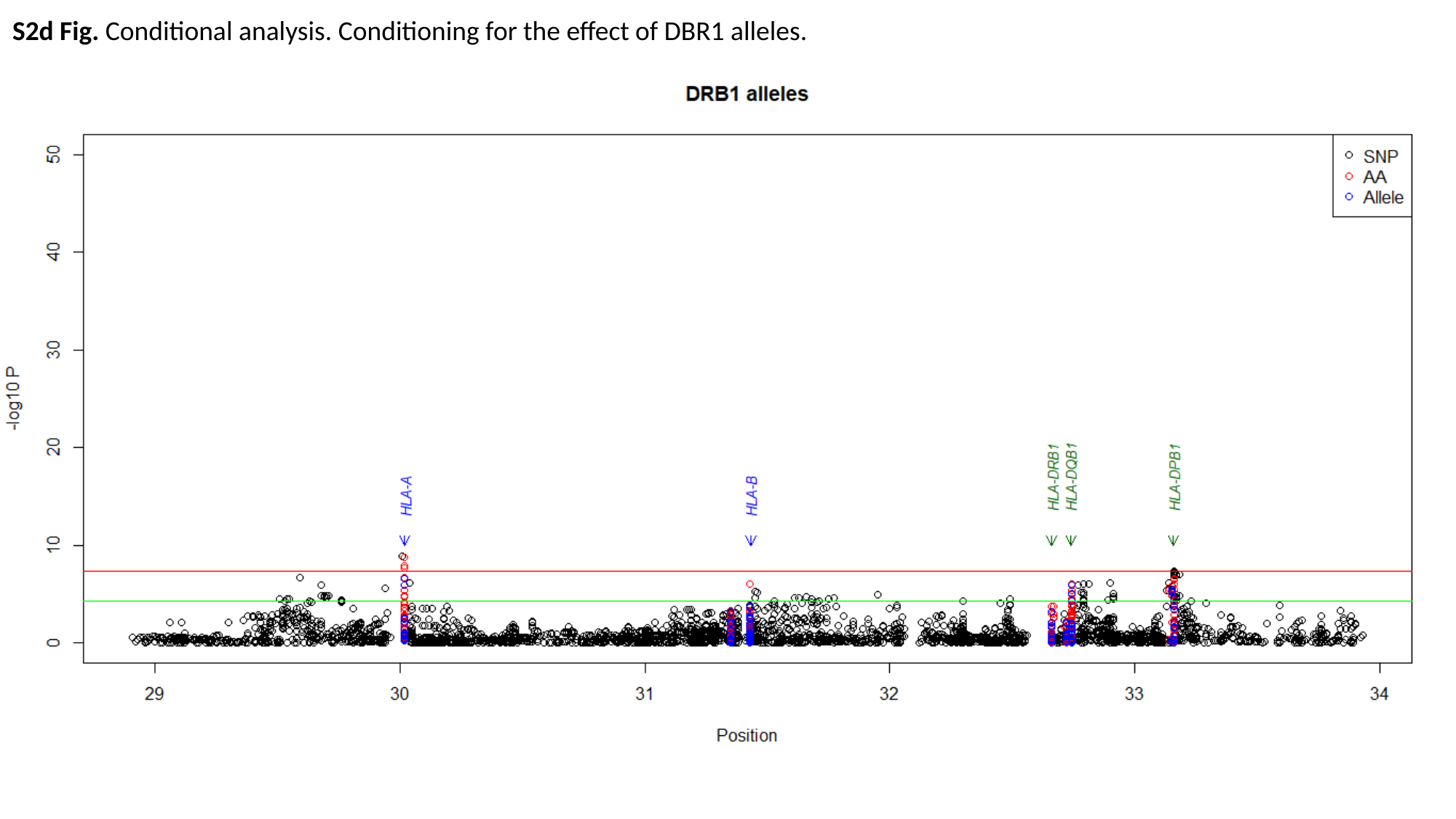

S2d Fig. Conditional analysis. Conditioning for the effect of DBR1 alleles.

## Slide 6
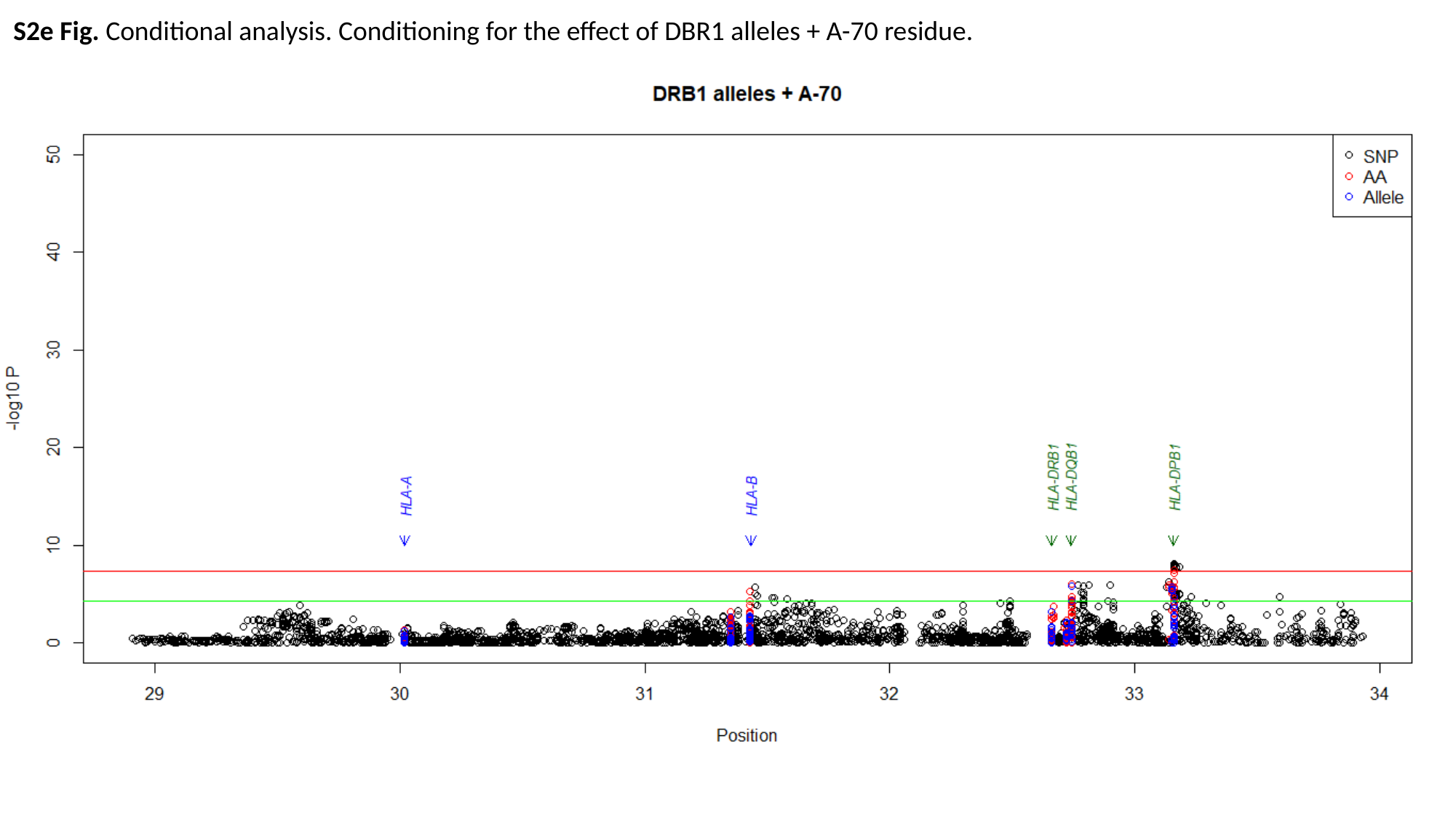

S2e Fig. Conditional analysis. Conditioning for the effect of DBR1 alleles + A-70 residue.

## Slide 7
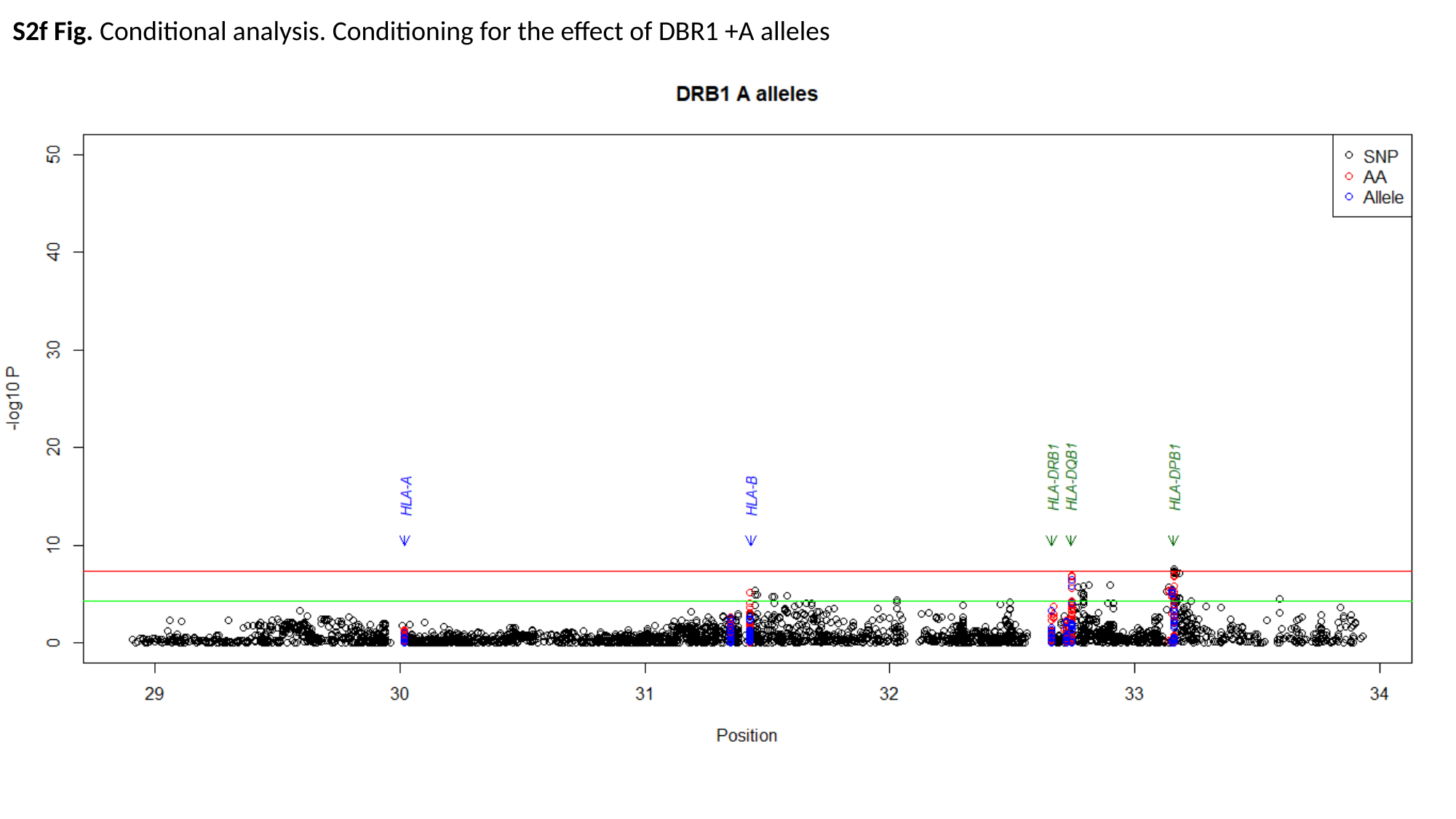

S2f Fig. Conditional analysis. Conditioning for the effect of DBR1 +A alleles

## Slide 8
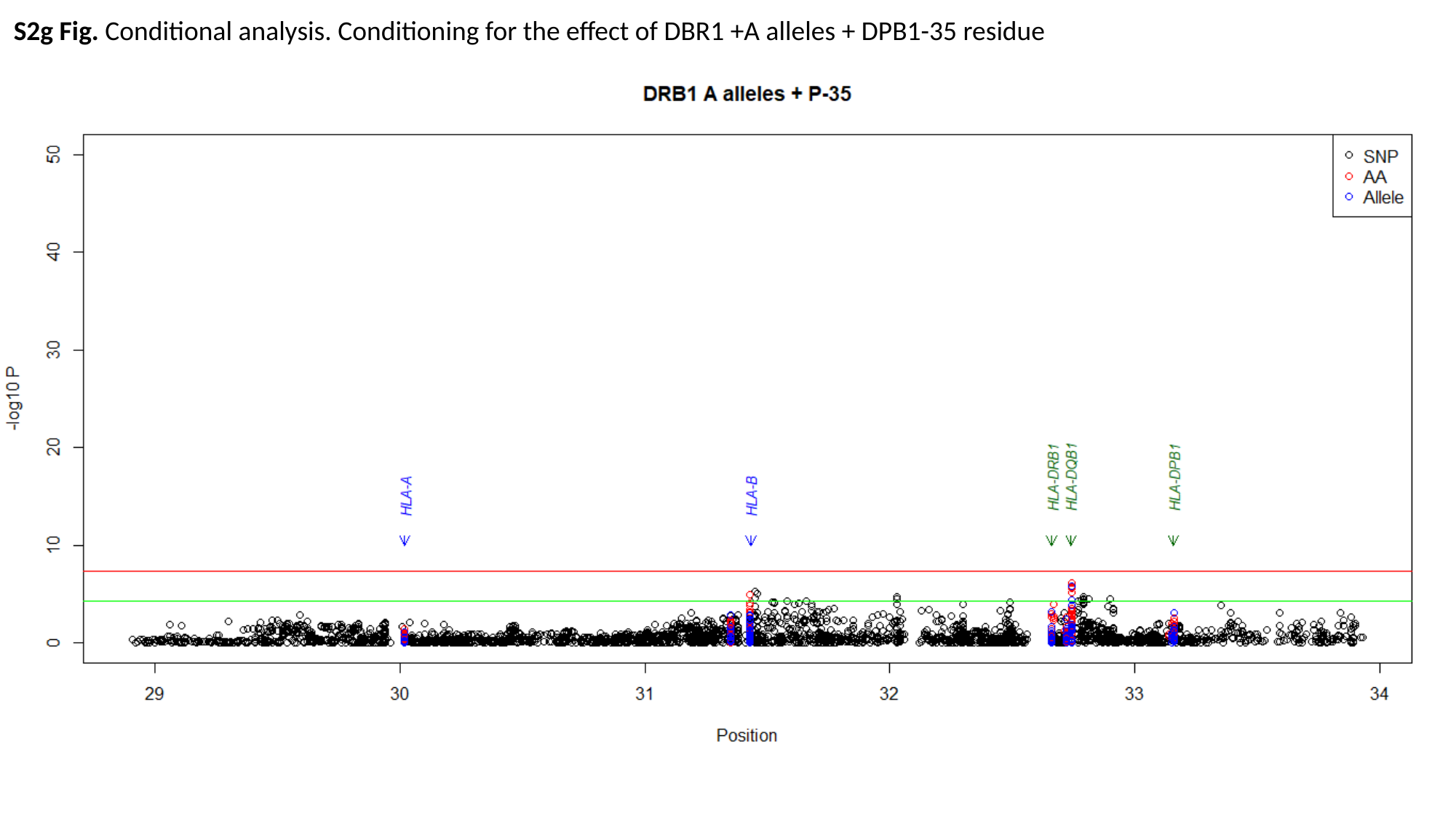

S2g Fig. Conditional analysis. Conditioning for the effect of DBR1 +A alleles + DPB1-35 residue

## Slide 9
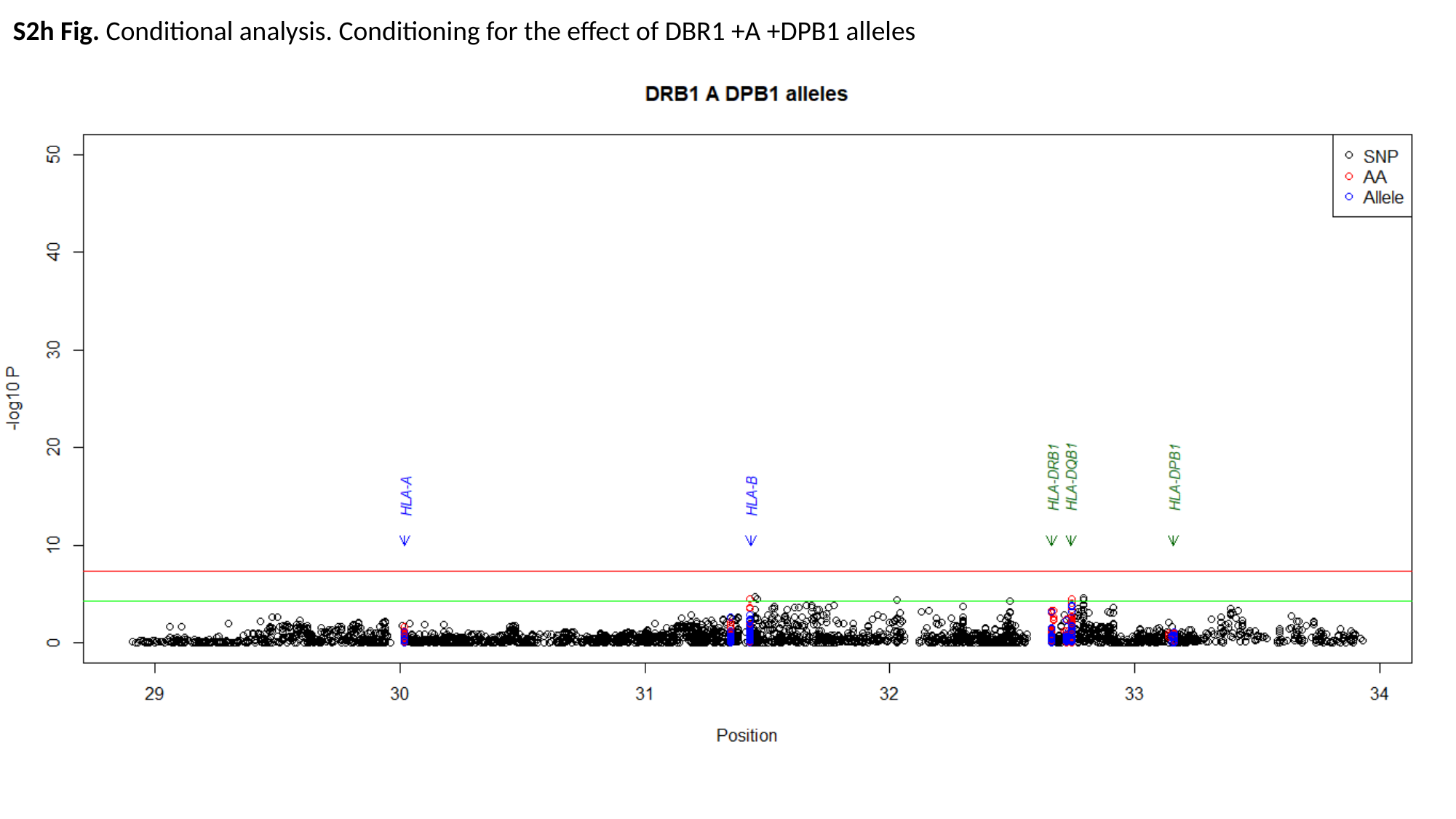

S2h Fig. Conditional analysis. Conditioning for the effect of DBR1 +A +DPB1 alleles

## Slide 10
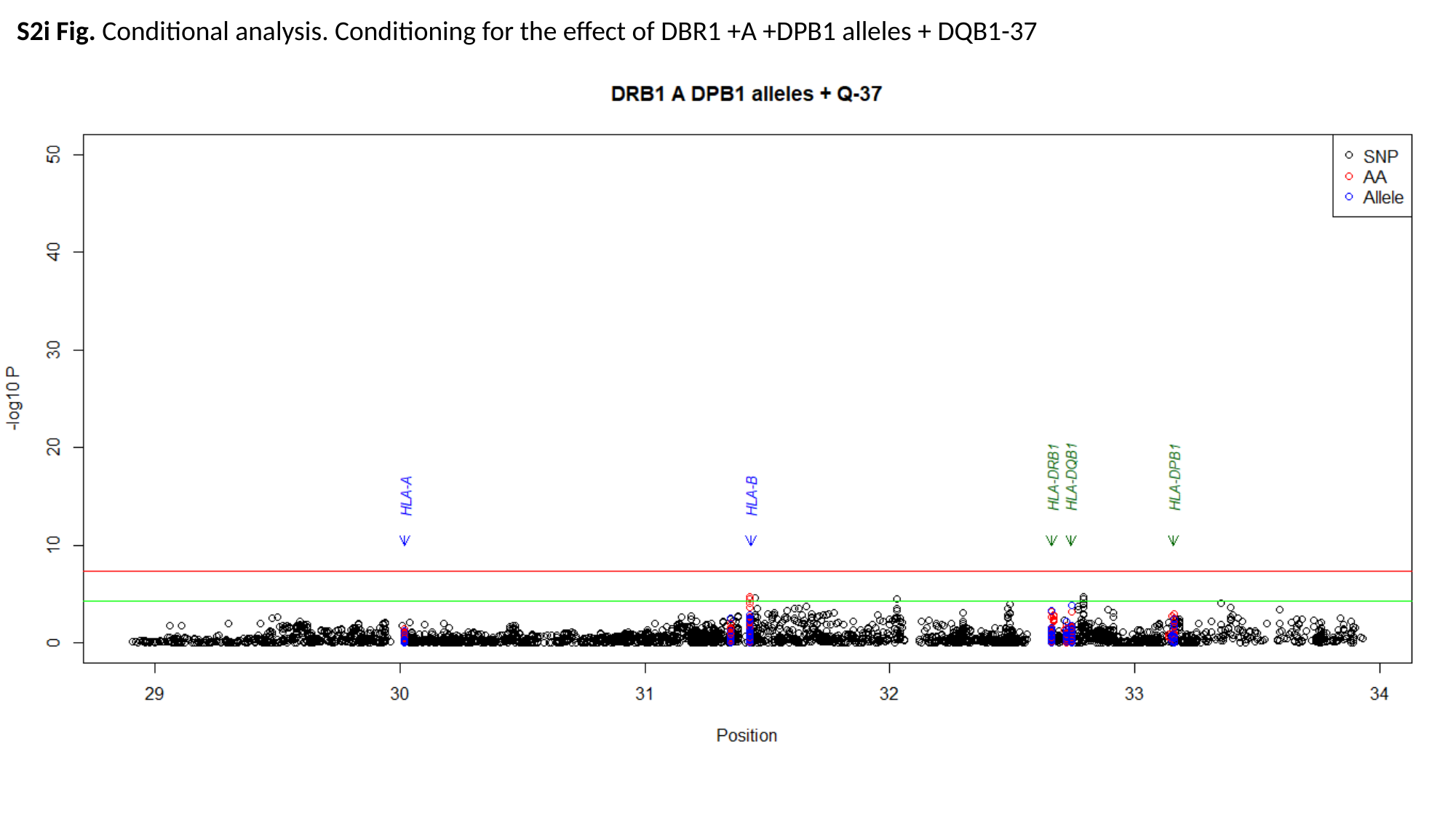

S2i Fig. Conditional analysis. Conditioning for the effect of DBR1 +A +DPB1 alleles + DQB1-37

## Slide 11
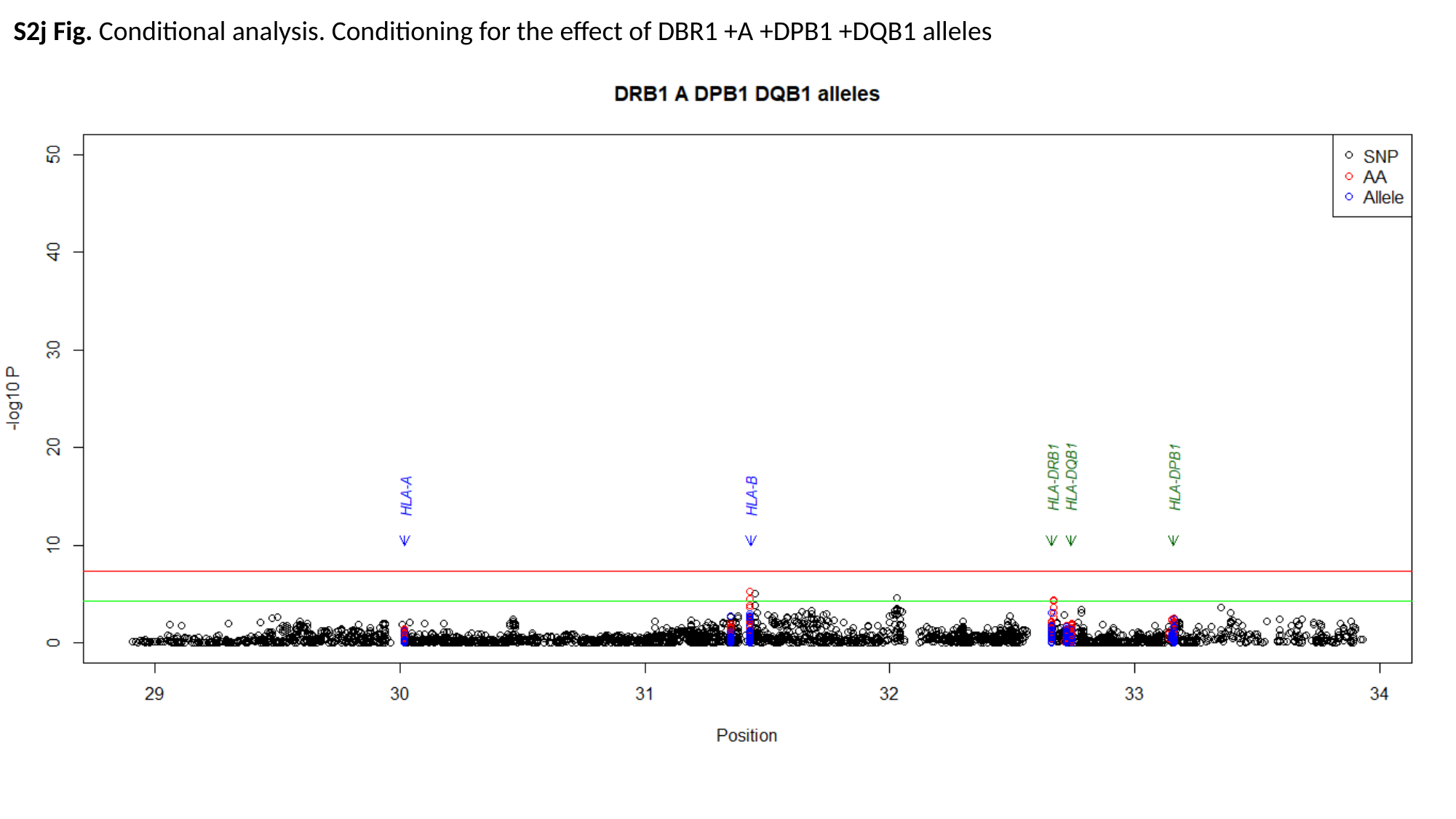

S2j Fig. Conditional analysis. Conditioning for the effect of DBR1 +A +DPB1 +DQB1 alleles

## Slide 12
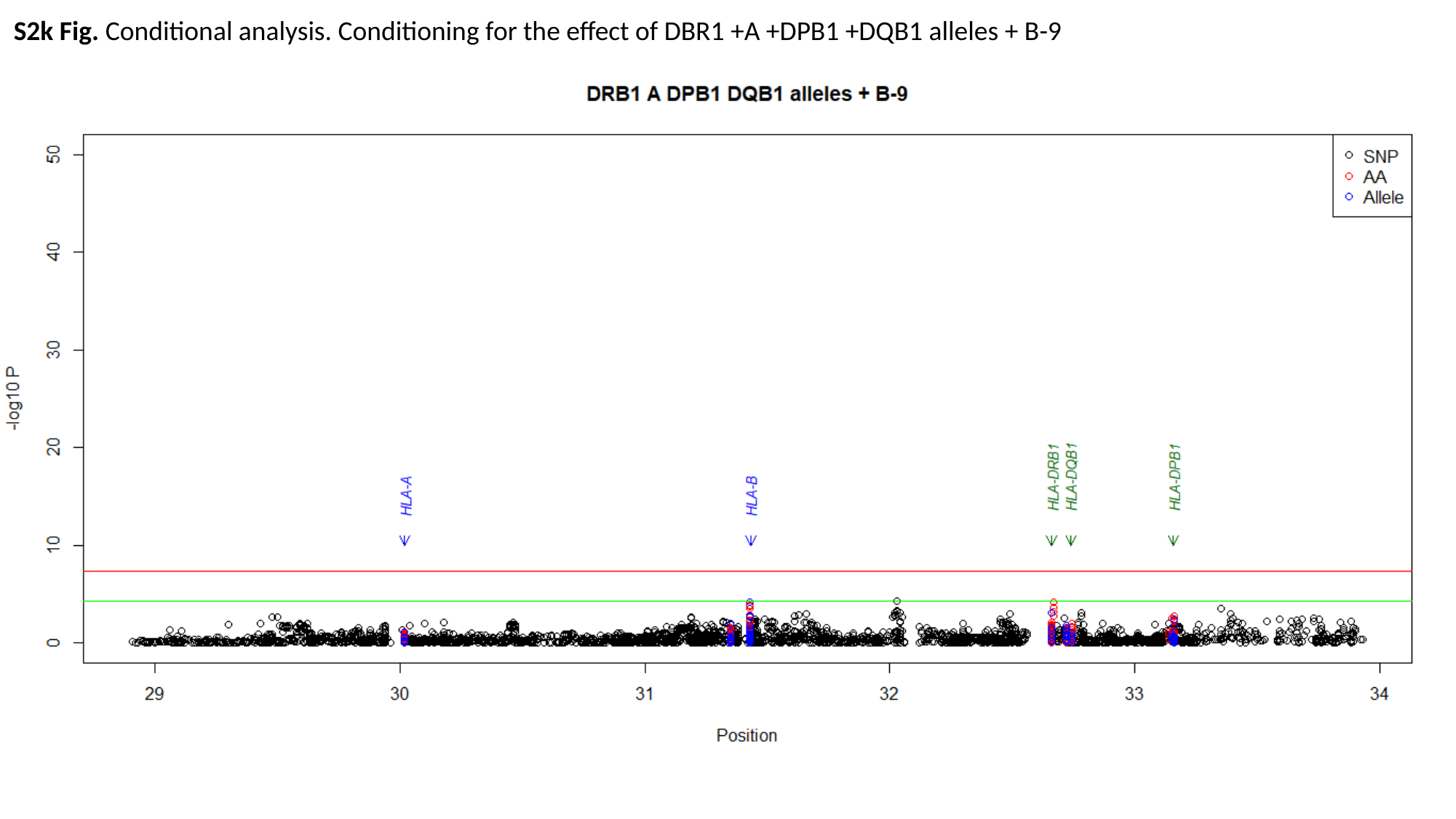

S2k Fig. Conditional analysis. Conditioning for the effect of DBR1 +A +DPB1 +DQB1 alleles + B-9

## Slide 13
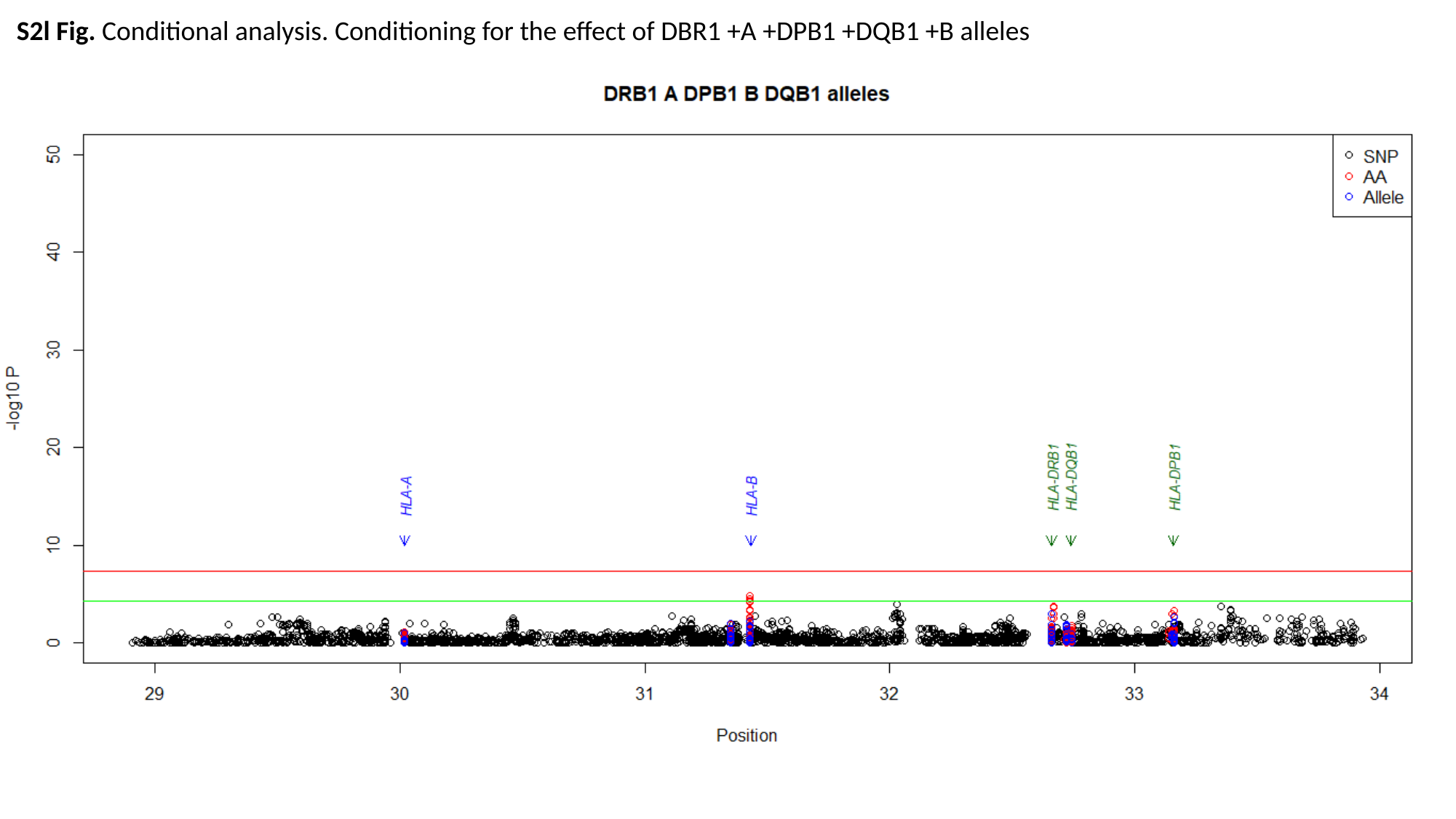

S2l Fig. Conditional analysis. Conditioning for the effect of DBR1 +A +DPB1 +DQB1 +B alleles
