# Supplementary material for: Amino acid signatures of HLA Class-I and II molecules are strongly associated with SLE susceptibility and autoantibody production in Eastern Asians
Source: PLoS Genet. 2019 Apr 25;15(4):e1008092. doi: 10.1371/journal.pgen.1008092 (PMC6504188; doi:10.1371/journal.pgen.1008092)
Supplement: S3 Fig — Trend for the increasing risk with increasing number of risk alleles was observed in both discovery and replication cohorts. There was a linear relationship between increased ORs and number of risk residues (R2 = 0.95; R2 = 0.90 for discovery and replication respectively), suggesting an additive effect. (PPTX) [file pgen.1008092.s003.pptx]

## Slide 1
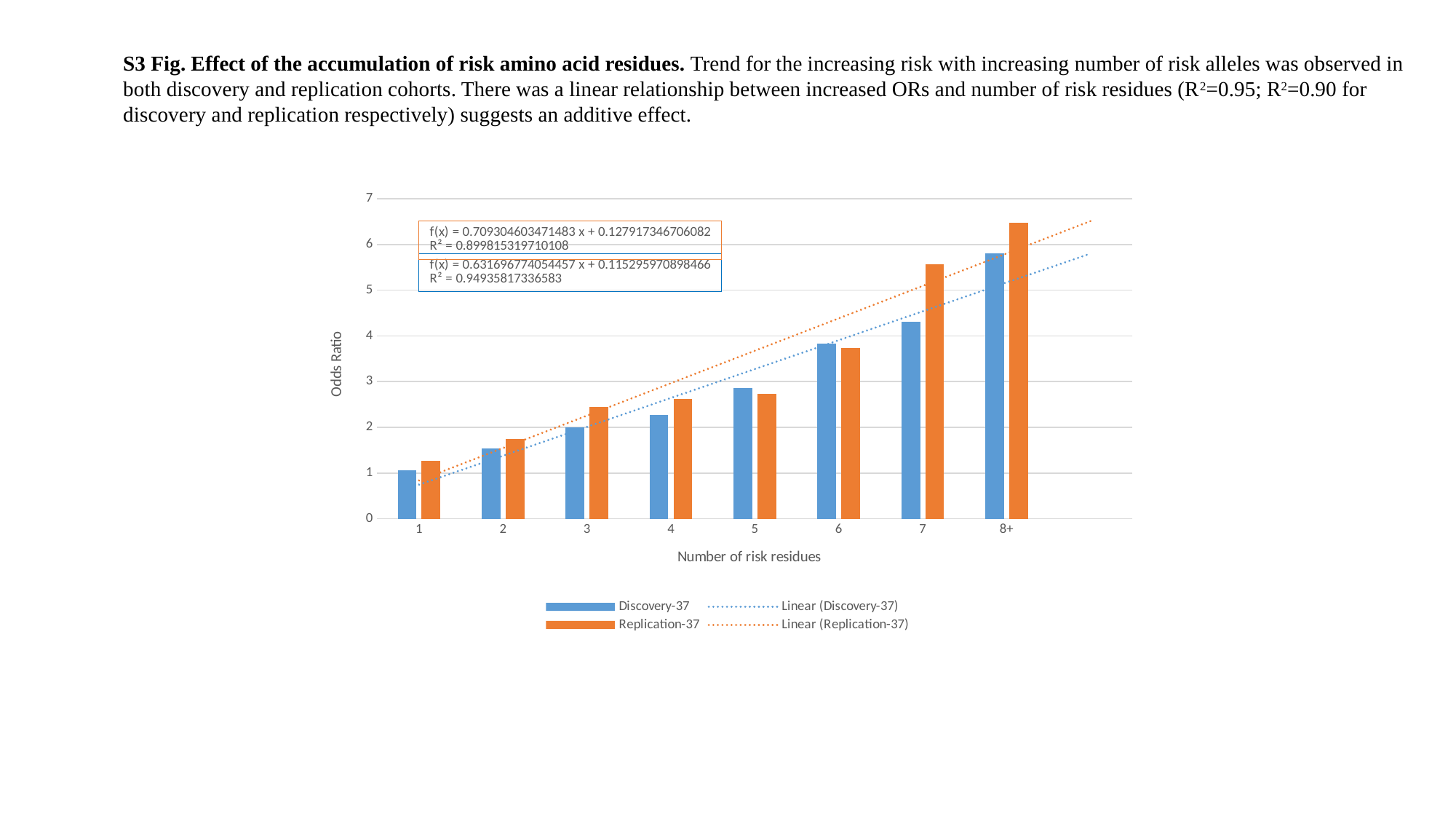

S3 Fig. Effect of the accumulation of risk amino acid residues. Trend for the increasing risk with increasing number of risk alleles was observed in both discovery and replication cohorts. There was a linear relationship between increased ORs and number of risk residues (R2=0.95; R2=0.90 for discovery and replication respectively) suggests an additive effect.
### Chart
| Category | | |
|---|---|---|
| 1 | 1.0640651144323758 | 1.2613315093479254 |
| 2 | 1.5371621621621618 | 1.7380415292960847 |
| 3 | 1.9989801121876594 | 2.440774839347504 |
| 4 | 2.270813527094935 | 2.618765627056191 |
| 5 | 2.854550663737943 | 2.7328618470288877 |
| 6 | 3.837227057566041 | 3.73069151324652 |
| 7 | 4.303126656067832 | 5.566797385620917 |
| 8+ | 5.79752633989922 | 6.46904024767802 |
