# Supplementary material for: Amino acid signatures of HLA Class-I and II molecules are strongly associated with SLE susceptibility and autoantibody production in Eastern Asians
Source: PLoS Genet. 2019 Apr 25;15(4):e1008092. doi: 10.1371/journal.pgen.1008092 (PMC6504188; doi:10.1371/journal.pgen.1008092)
Supplement: S4 Fig — Genomic position coordinates are presented as megabases for the hg19 genomic build. SNPs are presented in black, residues (AA) are presented in red, and alleles in blue. Lines for genome-wide association (P<5x10^-8^) in red, and for suggestive association (P<5x10^-5^) in green. (PPTX) [file pgen.1008092.s004.pptx]

## Slide 1
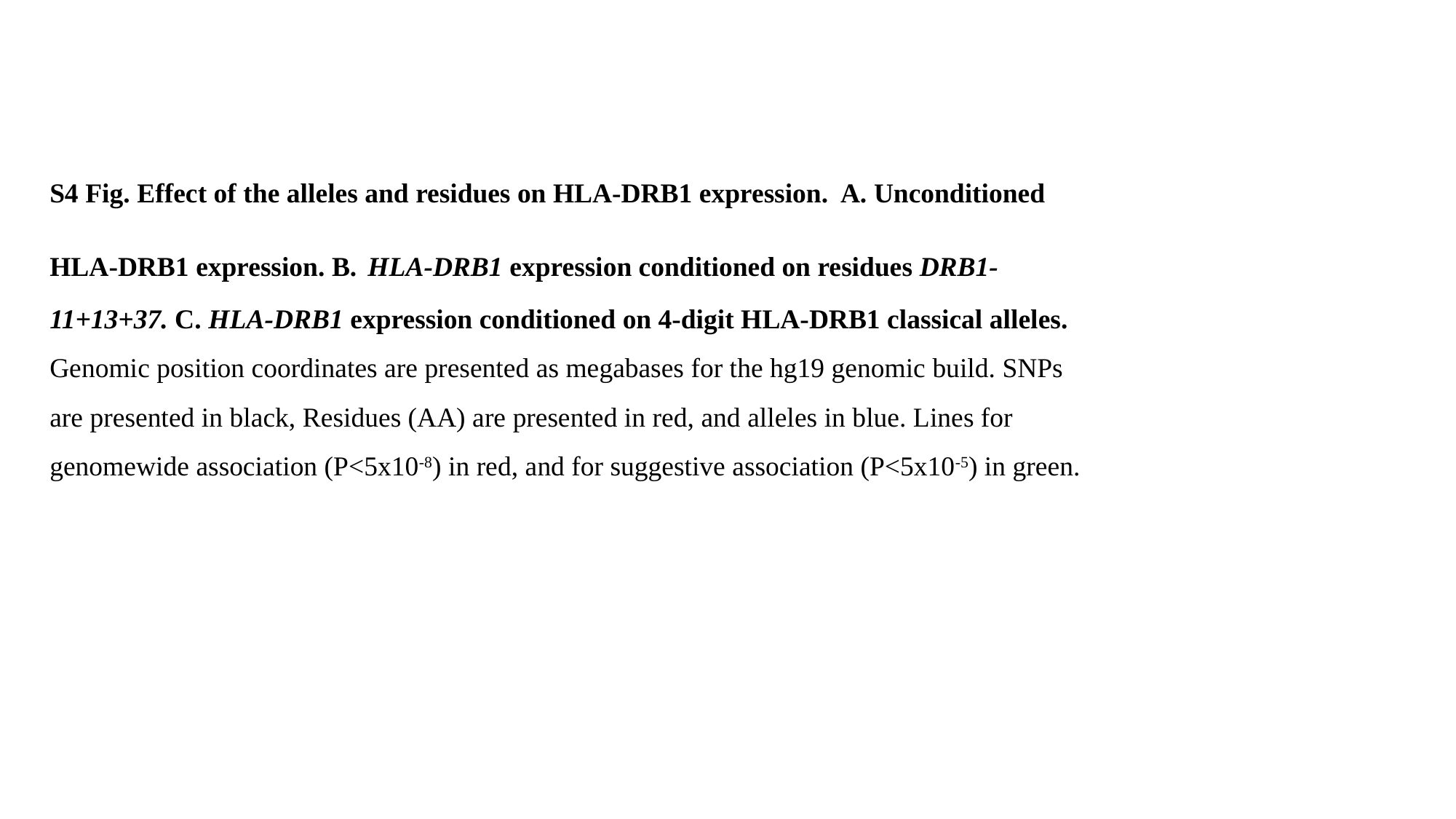

S4 Fig. Effect of the alleles and residues on HLA-DRB1 expression. A. Unconditioned HLA-DRB1 expression. B. HLA-DRB1 expression conditioned on residues DRB1-11+13+37. C. HLA-DRB1 expression conditioned on 4-digit HLA-DRB1 classical alleles. Genomic position coordinates are presented as megabases for the hg19 genomic build. SNPs are presented in black, Residues (AA) are presented in red, and alleles in blue. Lines for genomewide association (P<5x10^-8^) in red, and for suggestive association (P<5x10^-5^) in green.

## Slide 2
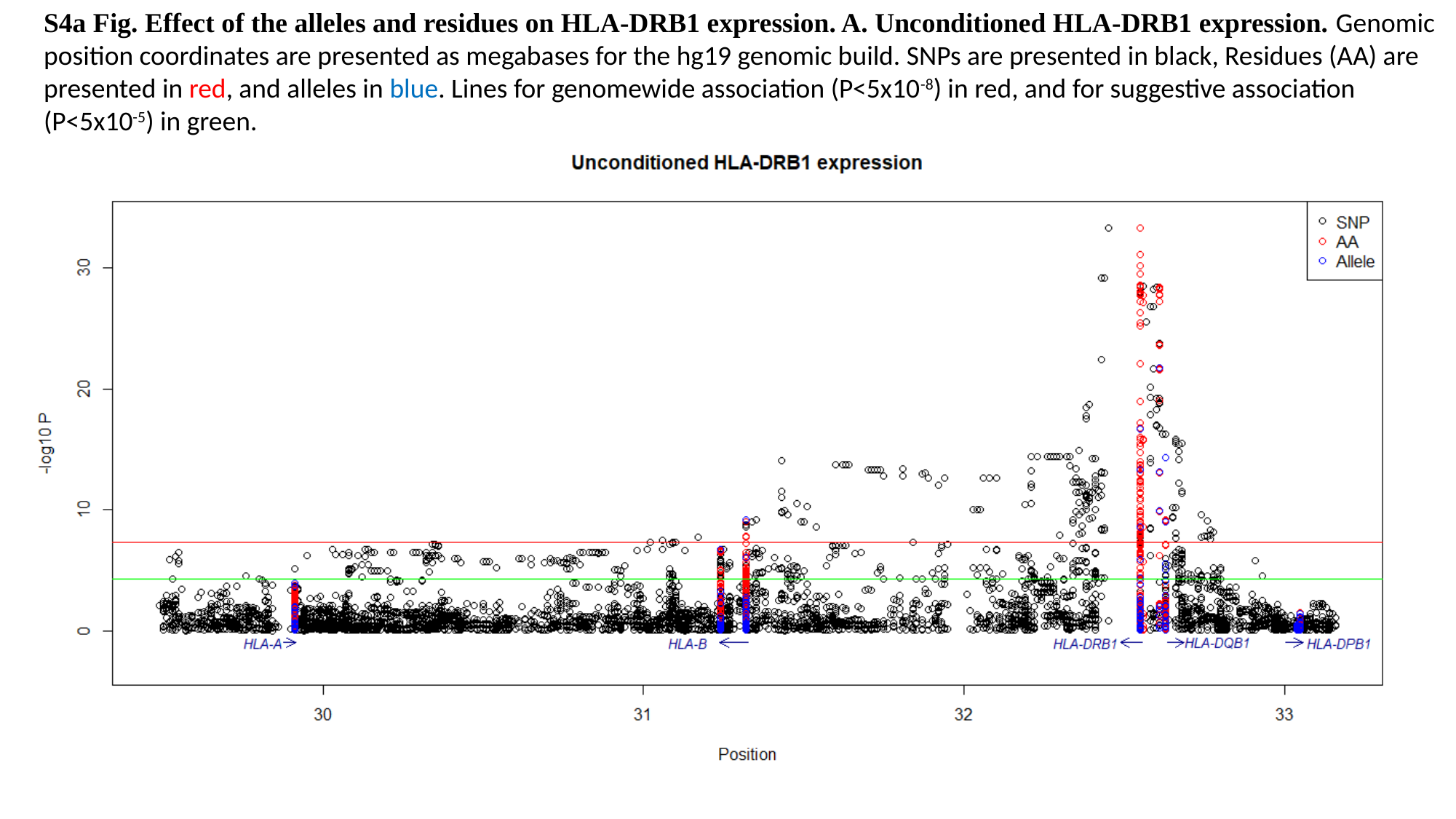

S4a Fig. Effect of the alleles and residues on HLA-DRB1 expression. A. Unconditioned HLA-DRB1 expression. Genomic position coordinates are presented as megabases for the hg19 genomic build. SNPs are presented in black, Residues (AA) are presented in red, and alleles in blue. Lines for genomewide association (P<5x10^-8^) in red, and for suggestive association (P<5x10^-5^) in green.

## Slide 3
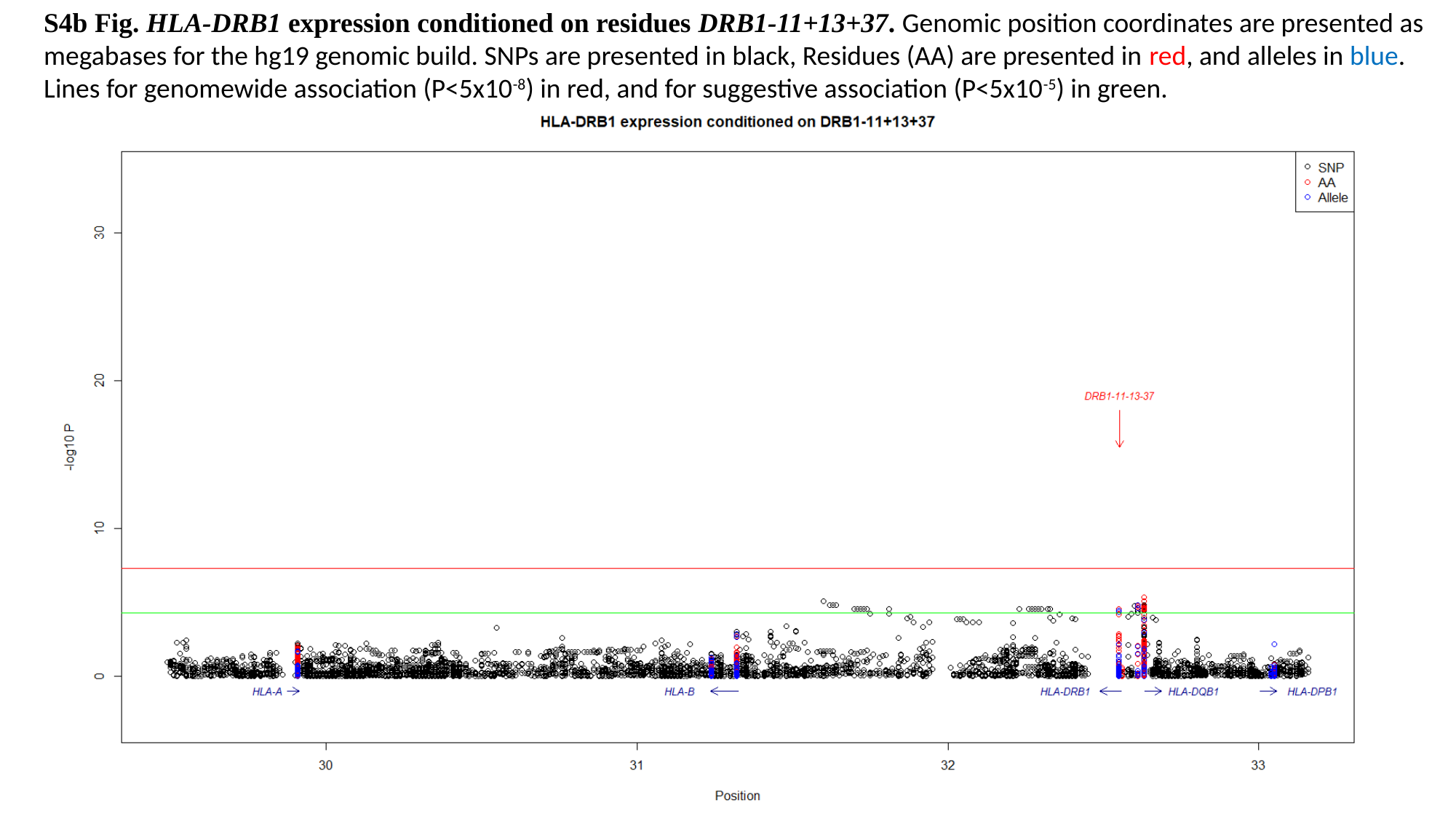

S4b Fig. HLA-DRB1 expression conditioned on residues DRB1-11+13+37. Genomic position coordinates are presented as megabases for the hg19 genomic build. SNPs are presented in black, Residues (AA) are presented in red, and alleles in blue. Lines for genomewide association (P<5x10^-8^) in red, and for suggestive association (P<5x10^-5^) in green.

## Slide 4
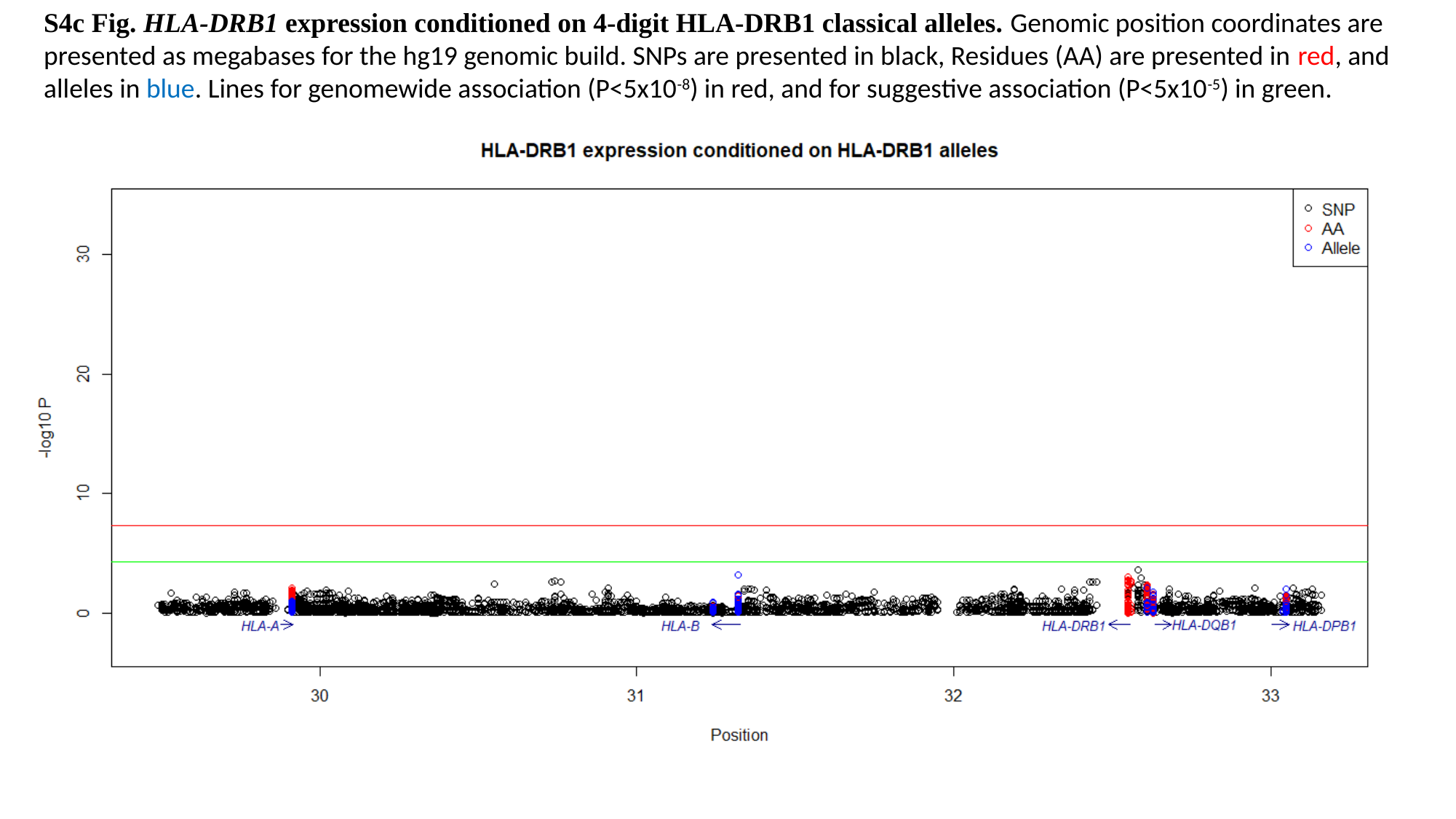

S4c Fig. HLA-DRB1 expression conditioned on 4-digit HLA-DRB1 classical alleles. Genomic position coordinates are presented as megabases for the hg19 genomic build. SNPs are presented in black, Residues (AA) are presented in red, and alleles in blue. Lines for genomewide association (P<5x10^-8^) in red, and for suggestive association (P<5x10^-5^) in green.
