# Supplementary material for: Amino acid signatures of HLA Class-I and II molecules are strongly associated with SLE susceptibility and autoantibody production in Eastern Asians
Source: PLoS Genet. 2019 Apr 25;15(4):e1008092. doi: 10.1371/journal.pgen.1008092 (PMC6504188; doi:10.1371/journal.pgen.1008092)
Supplement: S5 Fig — Accumulation of Class I risk residues in the peptide-binding groove is color-coded according to the strength of association (from blue to red). A. Accumulation of Class I risk residues in the peptide-binding groove; B. Accumulation of Class I risk residues in the peptide-binding groove (zoom); C. Risk residues in the peptide-binding groove of HLA-A.; D. Risk residues in the peptide-binding groove of HLA-A (zoom); E. Risk residues in the peptide-binding groove of HLA-A. Post-conditioning on DRB1-11/13, DRB1-37, DQB1-37, A-70, DPB1-35, and B-9; F. Risk residues in the peptide-binding groove of HLA-B; G. Risk residues in the peptide-binding groove of HLA-B (zoom); H. Risk residues in the peptide-binding groove of HLA-B. Post-conditioning on DRB1-11/13, DRB1-37, DQB1-37, A-70, DPB1-35, and B-9; I. Risk residues in the peptide-binding groove of HLA-B. Post-conditioning on DRB1-11/13, DRB1-37, DQB1-37, A-70, DPB1-35, and B-9 (zoom). B-63 emerges as a significantly associated residue in addition to B-9; J. Risk residues in the peptide-binding groove of HLA-C; K. Risk residues in the peptide-binding groove of HLA-C (zoom); L. Risk residues in the peptide-binding groove of HLA-C. Post-conditioning on DRB1-11/13, DRB1-37, DQB1-37, A-70, DPB1-35, and B-9. (PPTX) [file pgen.1008092.s005.pptx]

## Slide 1
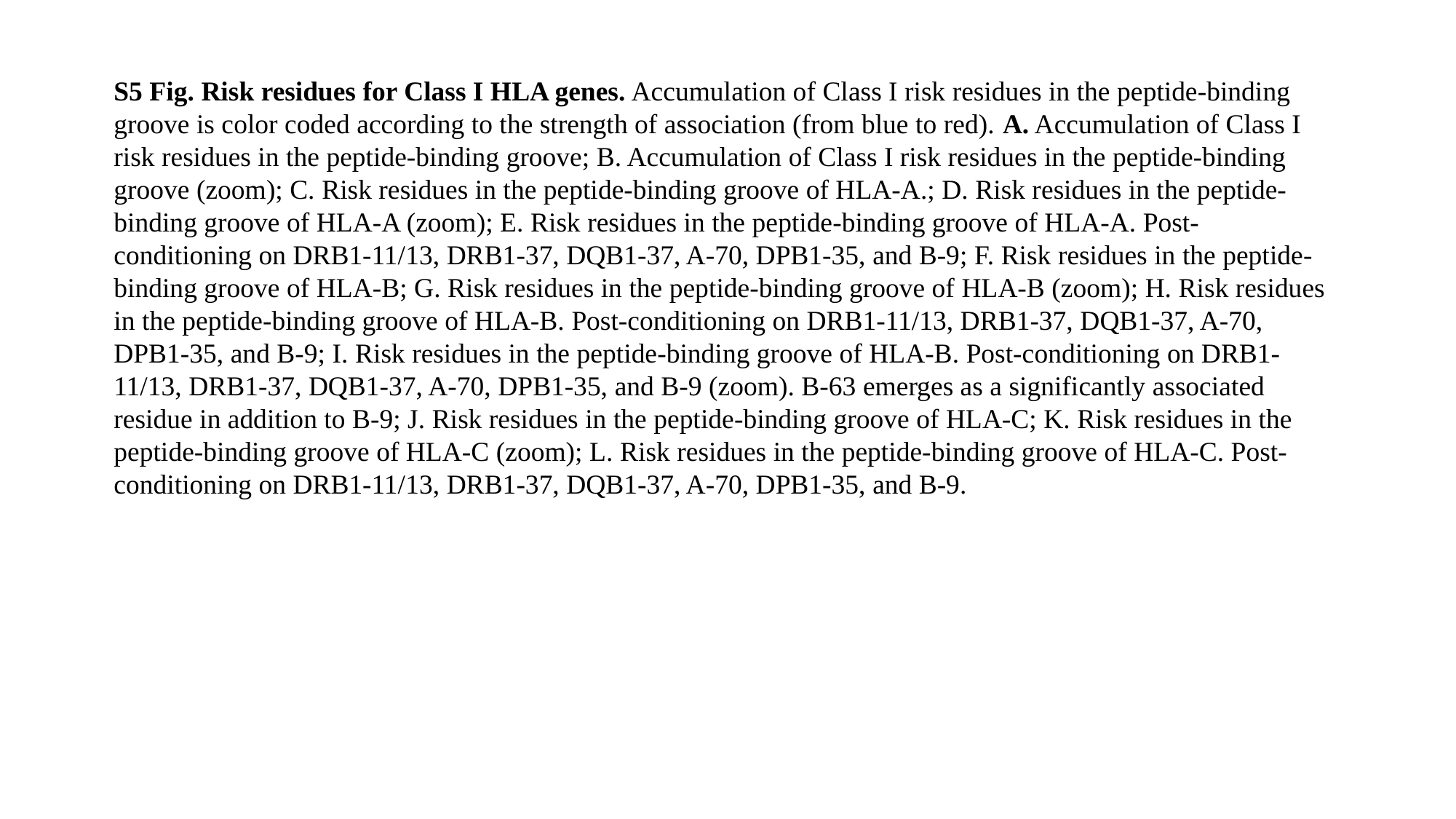

S5 Fig. Risk residues for Class I HLA genes. Accumulation of Class I risk residues in the peptide-binding groove is color coded according to the strength of association (from blue to red). A. Accumulation of Class I risk residues in the peptide-binding groove; B. Accumulation of Class I risk residues in the peptide-binding groove (zoom); C. Risk residues in the peptide-binding groove of HLA-A.; D. Risk residues in the peptide-binding groove of HLA-A (zoom); E. Risk residues in the peptide-binding groove of HLA-A. Post-conditioning on DRB1-11/13, DRB1-37, DQB1-37, A-70, DPB1-35, and B-9; F. Risk residues in the peptide-binding groove of HLA-B; G. Risk residues in the peptide-binding groove of HLA-B (zoom); H. Risk residues in the peptide-binding groove of HLA-B. Post-conditioning on DRB1-11/13, DRB1-37, DQB1-37, A-70, DPB1-35, and B-9; I. Risk residues in the peptide-binding groove of HLA-B. Post-conditioning on DRB1-11/13, DRB1-37, DQB1-37, A-70, DPB1-35, and B-9 (zoom). B-63 emerges as a significantly associated residue in addition to B-9; J. Risk residues in the peptide-binding groove of HLA-C; K. Risk residues in the peptide-binding groove of HLA-C (zoom); L. Risk residues in the peptide-binding groove of HLA-C. Post-conditioning on DRB1-11/13, DRB1-37, DQB1-37, A-70, DPB1-35, and B-9.

## Slide 2
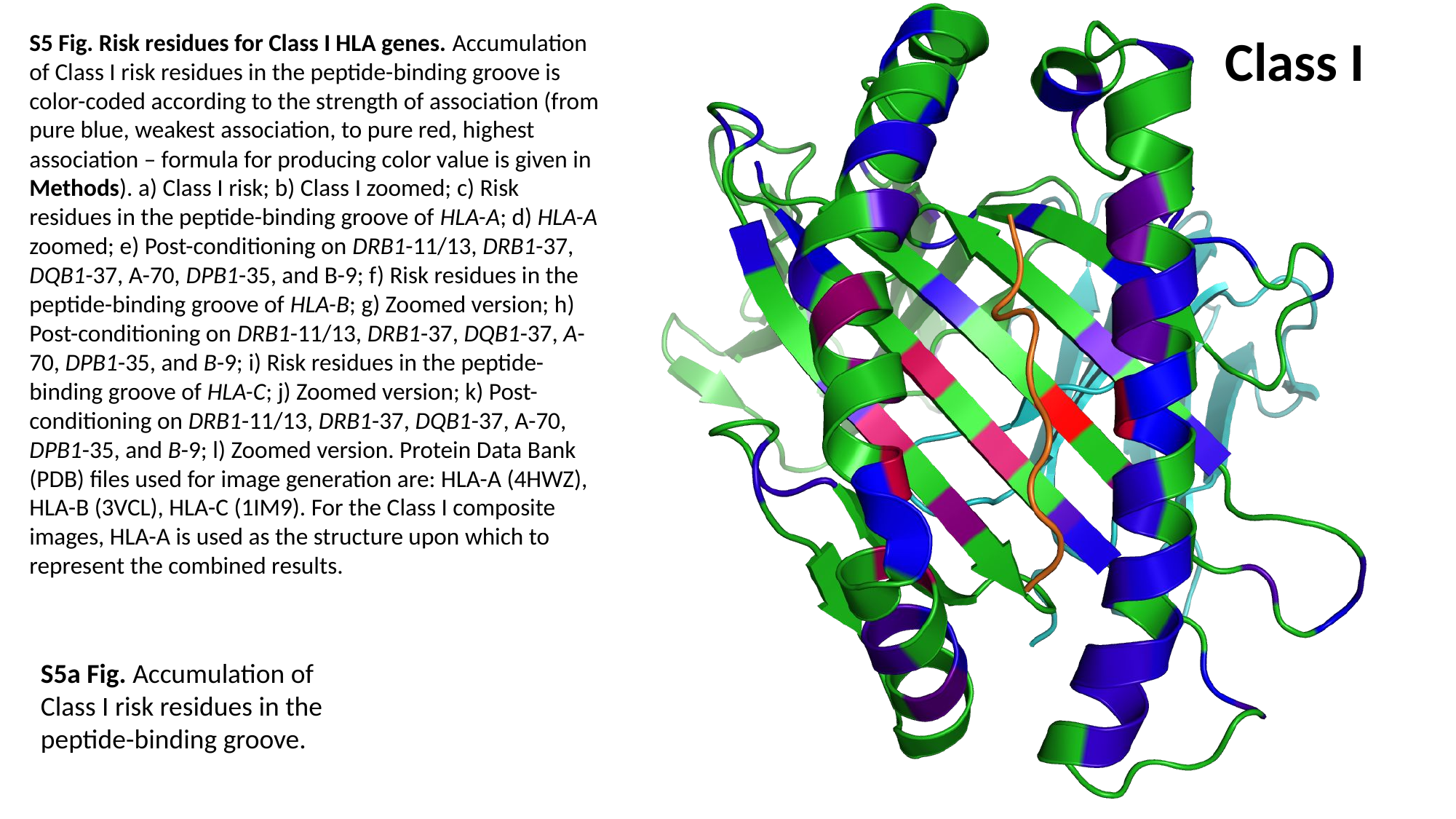

S5 Fig. Risk residues for Class I HLA genes. Accumulation of Class I risk residues in the peptide-binding groove is color-coded according to the strength of association (from pure blue, weakest association, to pure red, highest association – formula for producing color value is given in Methods). a) Class I risk; b) Class I zoomed; c) Risk residues in the peptide-binding groove of HLA-A; d) HLA-A zoomed; e) Post-conditioning on DRB1-11/13, DRB1-37, DQB1-37, A-70, DPB1-35, and B-9; f) Risk residues in the peptide-binding groove of HLA-B; g) Zoomed version; h) Post-conditioning on DRB1-11/13, DRB1-37, DQB1-37, A-70, DPB1-35, and B-9; i) Risk residues in the peptide-binding groove of HLA-C; j) Zoomed version; k) Post-conditioning on DRB1-11/13, DRB1-37, DQB1-37, A-70, DPB1-35, and B-9; l) Zoomed version. Protein Data Bank (PDB) files used for image generation are: HLA-A (4HWZ), HLA-B (3VCL), HLA-C (1IM9). For the Class I composite images, HLA-A is used as the structure upon which to represent the combined results.
Class I
S5a Fig. Accumulation of Class I risk residues in the peptide-binding groove.

## Slide 3
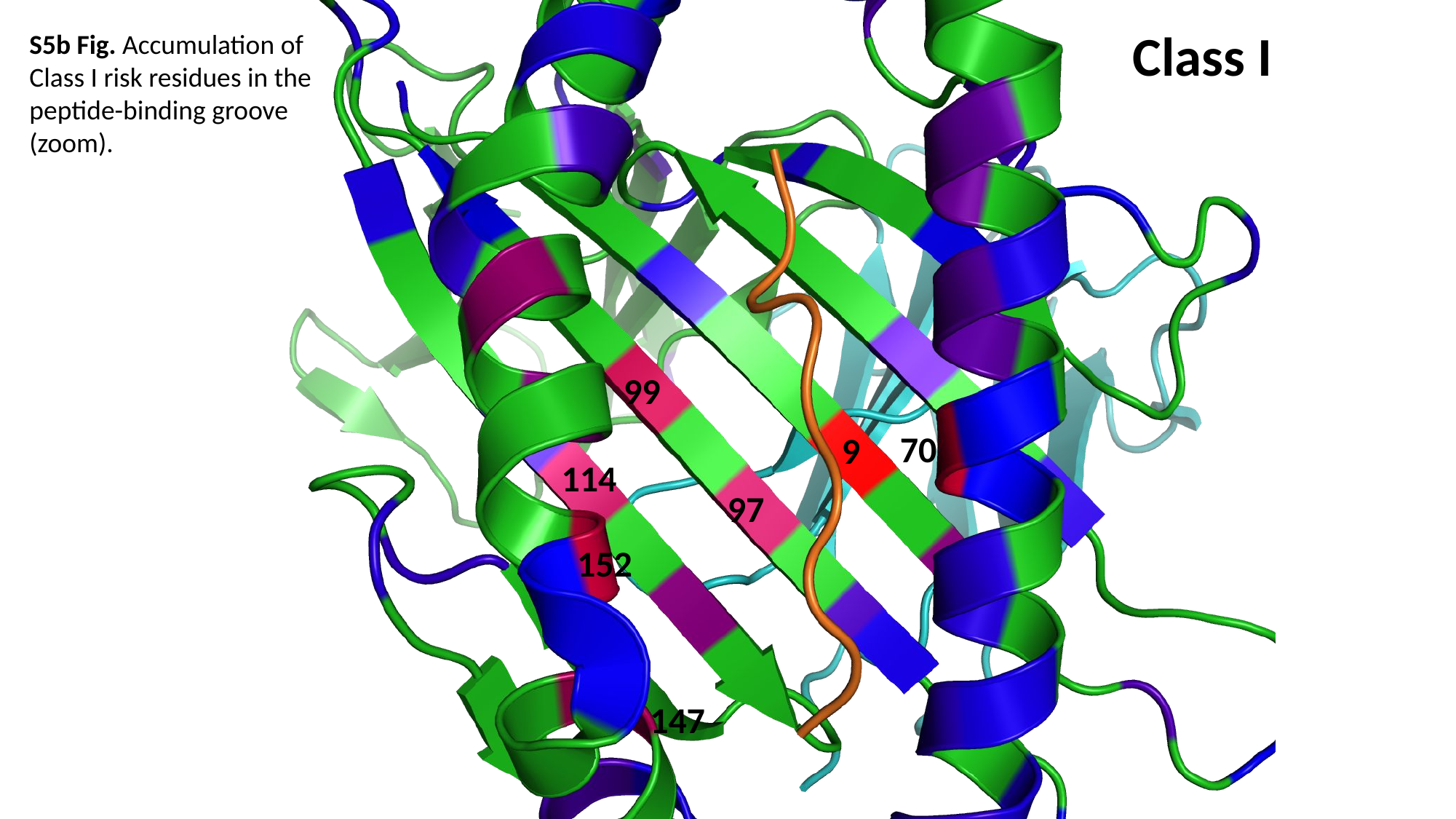

Class I
S5b Fig. Accumulation of Class I risk residues in the peptide-binding groove (zoom).
99
70
9
114
97
152
147

## Slide 4
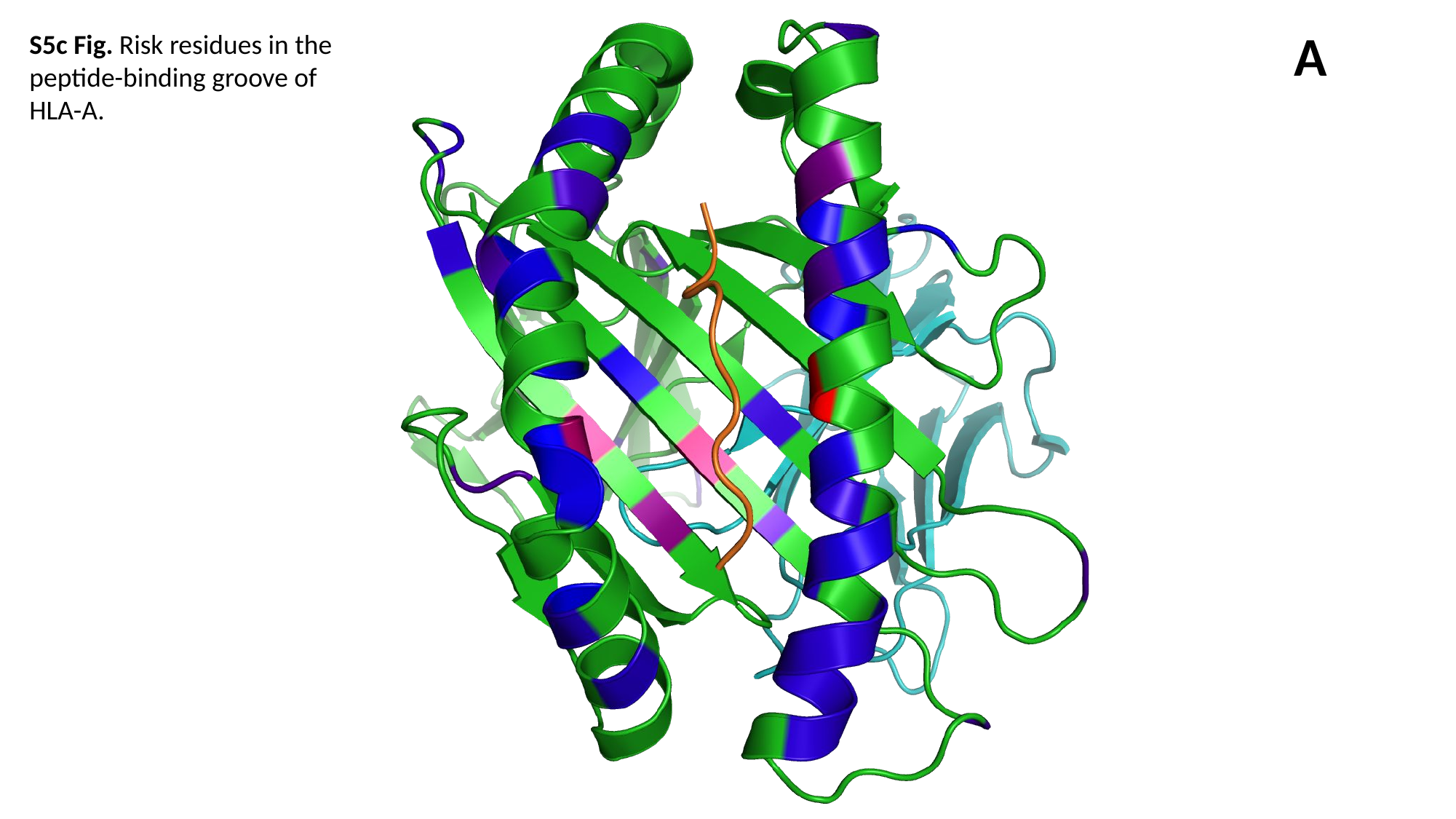

A
S5c Fig. Risk residues in the peptide-binding groove of HLA-A.

## Slide 5
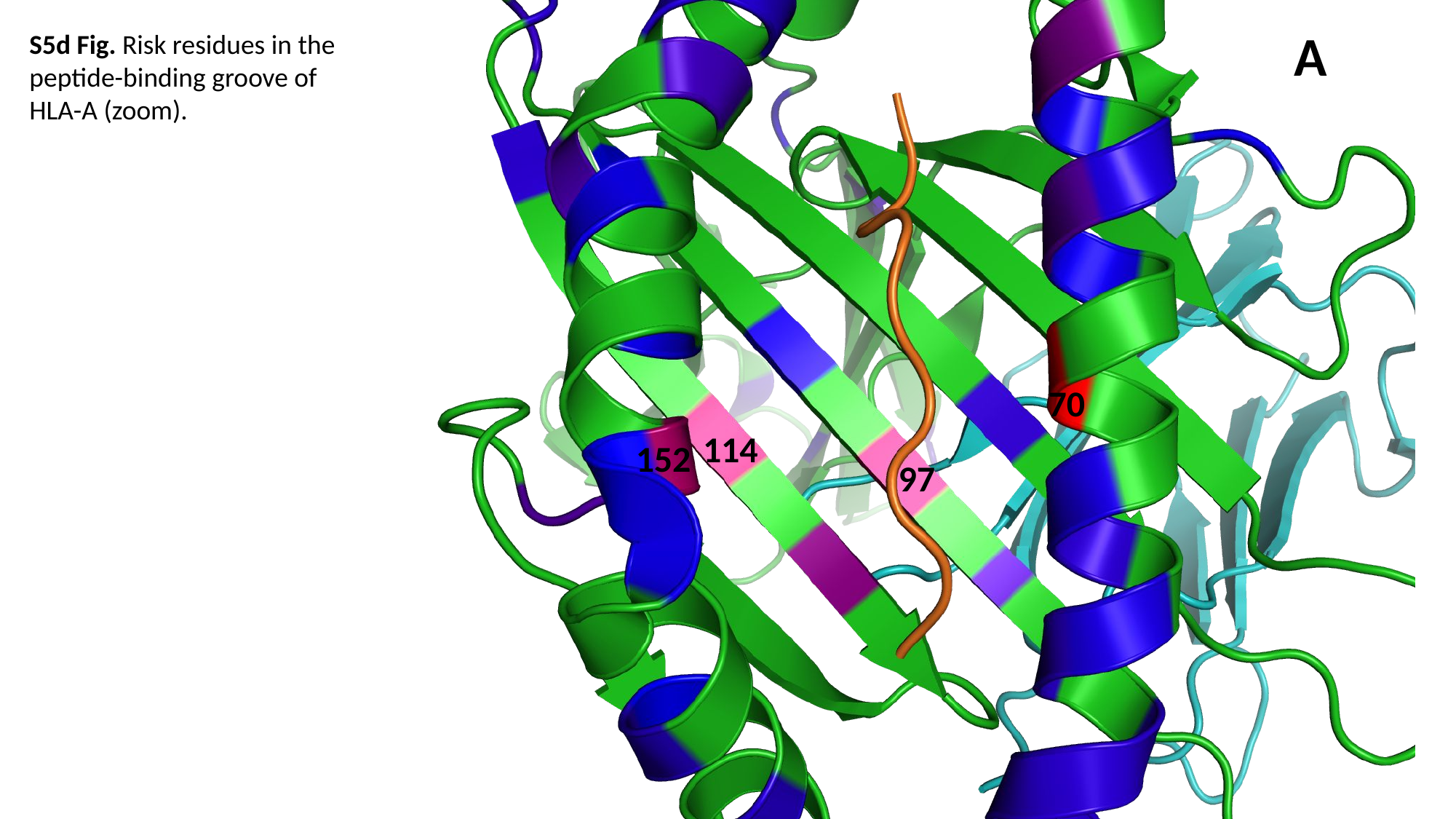

A
S5d Fig. Risk residues in the peptide-binding groove of HLA-A (zoom).
70
114
152
97

## Slide 6
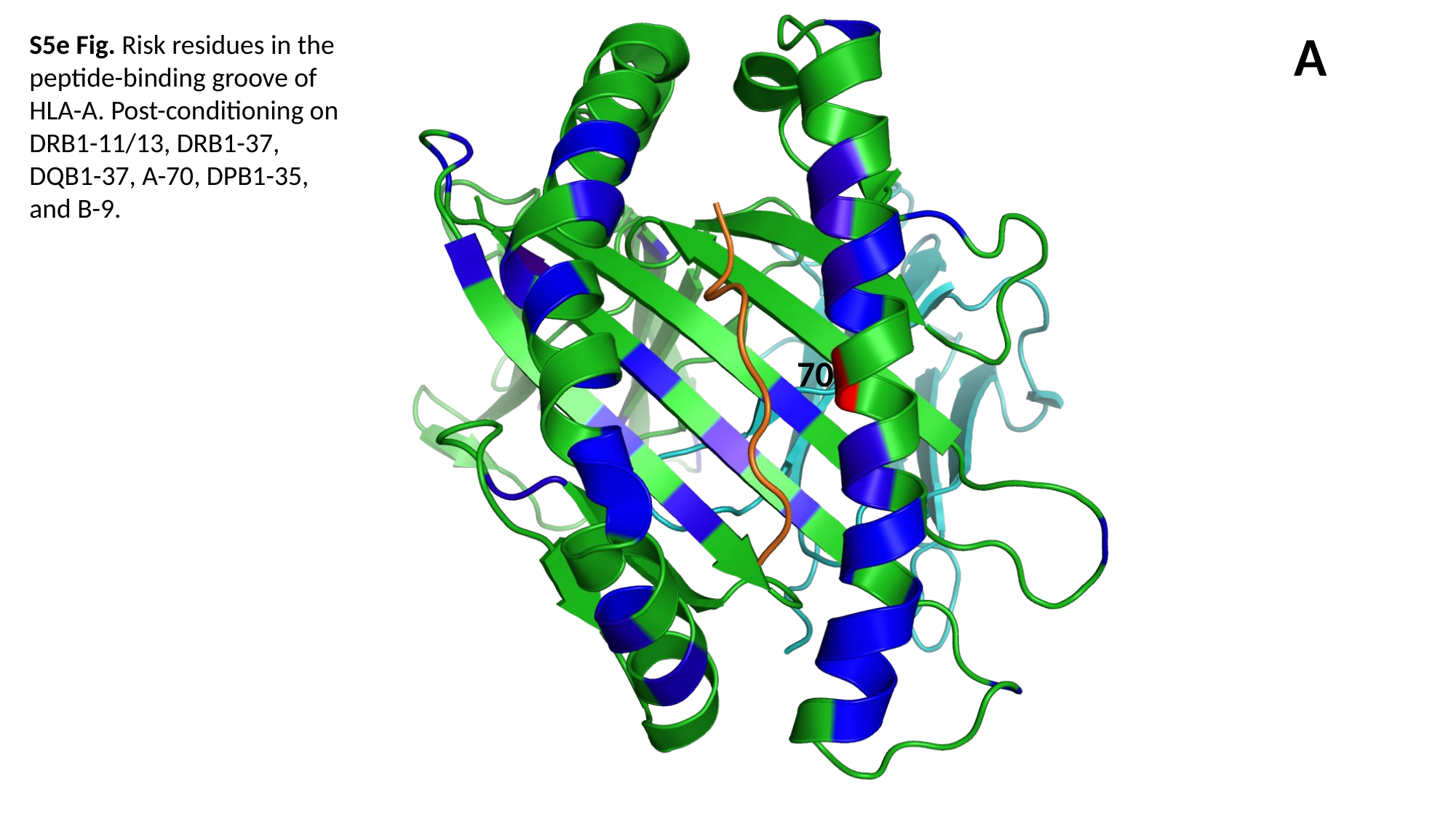

A
S5e Fig. Risk residues in the peptide-binding groove of HLA-A. Post-conditioning on DRB1-11/13, DRB1-37, DQB1-37, A-70, DPB1-35, and B-9.
70

## Slide 7
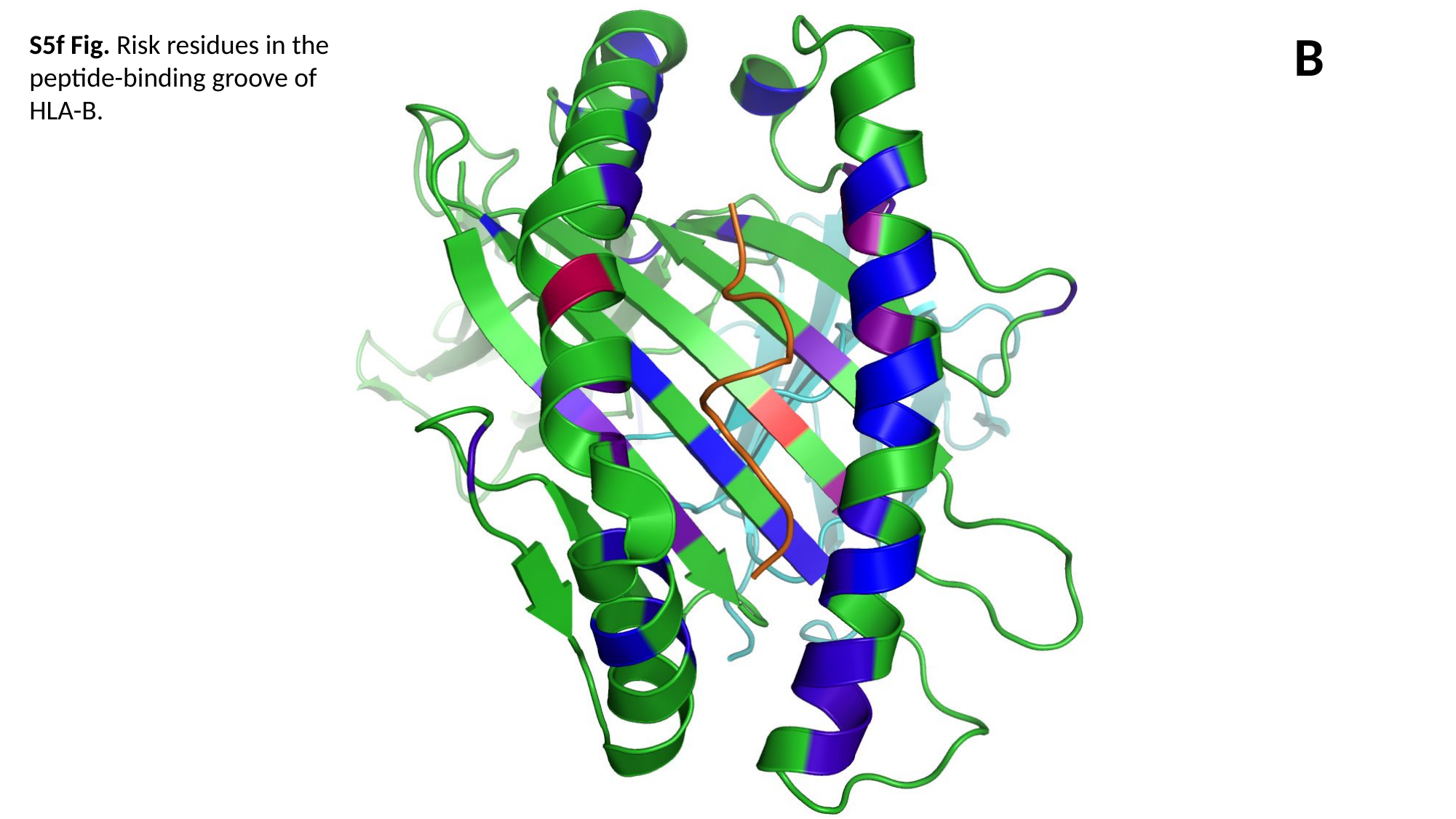

B
S5f Fig. Risk residues in the peptide-binding groove of HLA-B.

## Slide 8
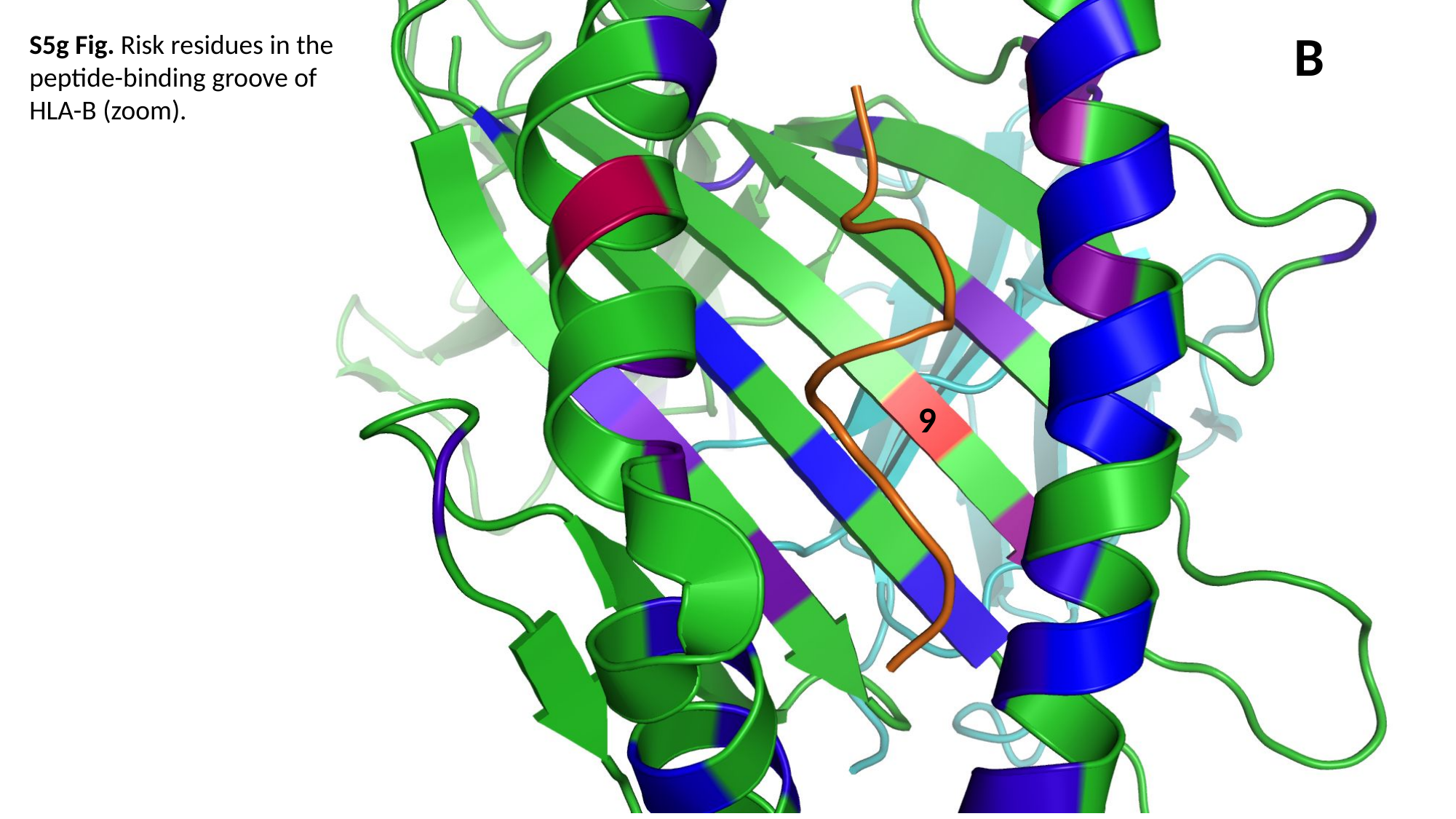

B
S5g Fig. Risk residues in the peptide-binding groove of HLA-B (zoom).
9

## Slide 9
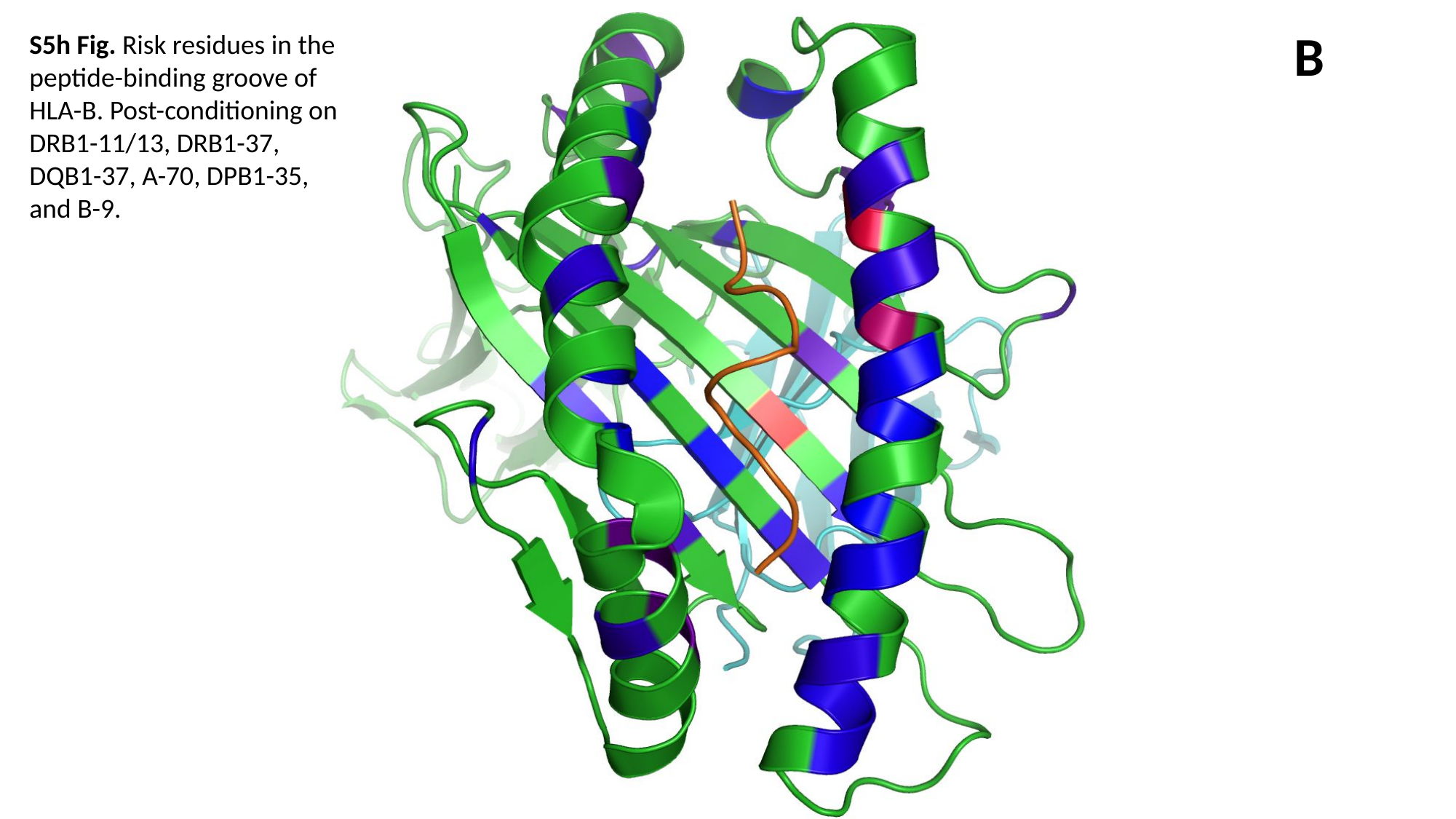

B
S5h Fig. Risk residues in the peptide-binding groove of HLA-B. Post-conditioning on DRB1-11/13, DRB1-37, DQB1-37, A-70, DPB1-35, and B-9.

## Slide 10
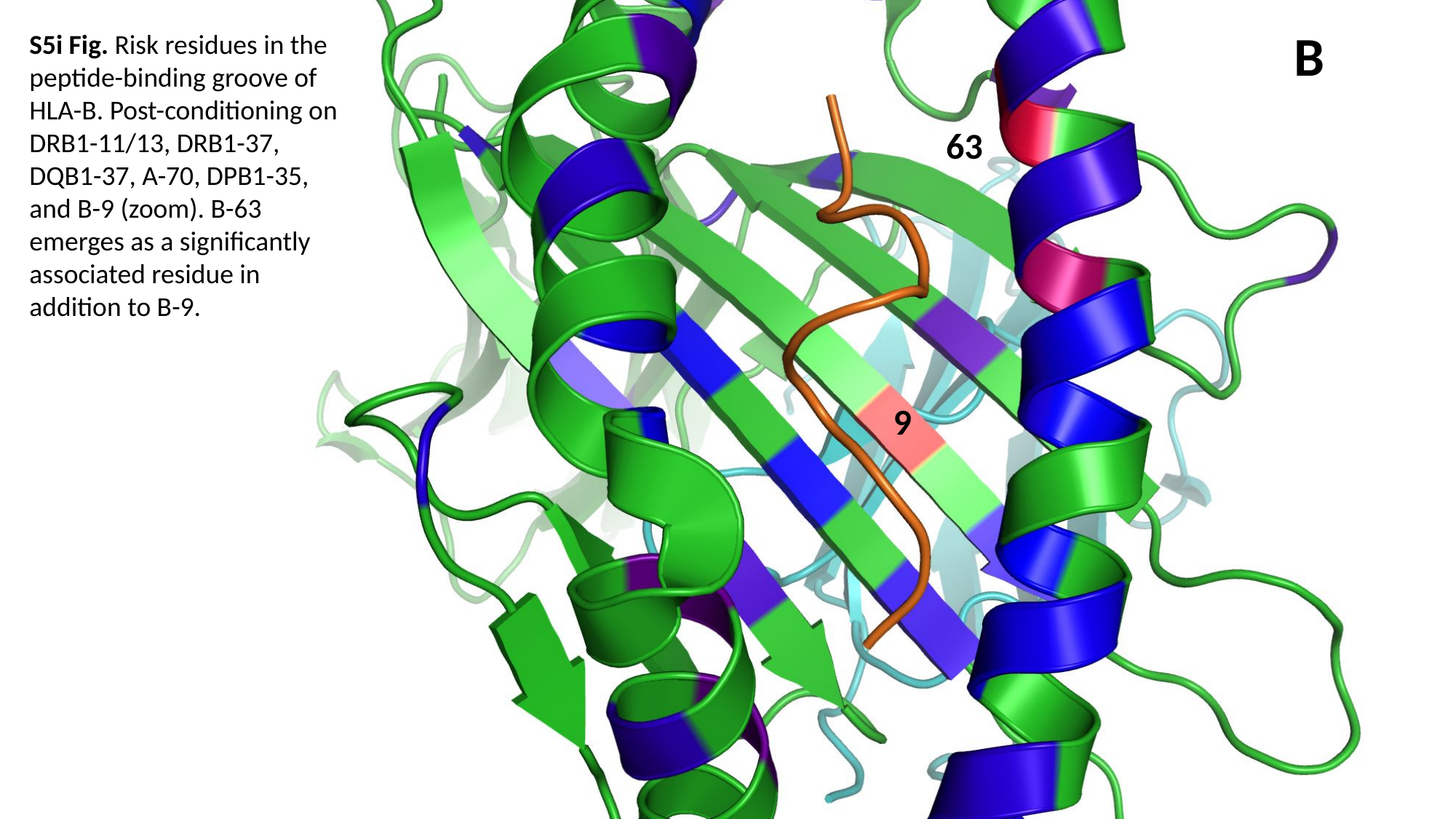

B
S5i Fig. Risk residues in the peptide-binding groove of HLA-B. Post-conditioning on DRB1-11/13, DRB1-37, DQB1-37, A-70, DPB1-35, and B-9 (zoom). B-63 emerges as a significantly associated residue in addition to B-9.
63
9

## Slide 11
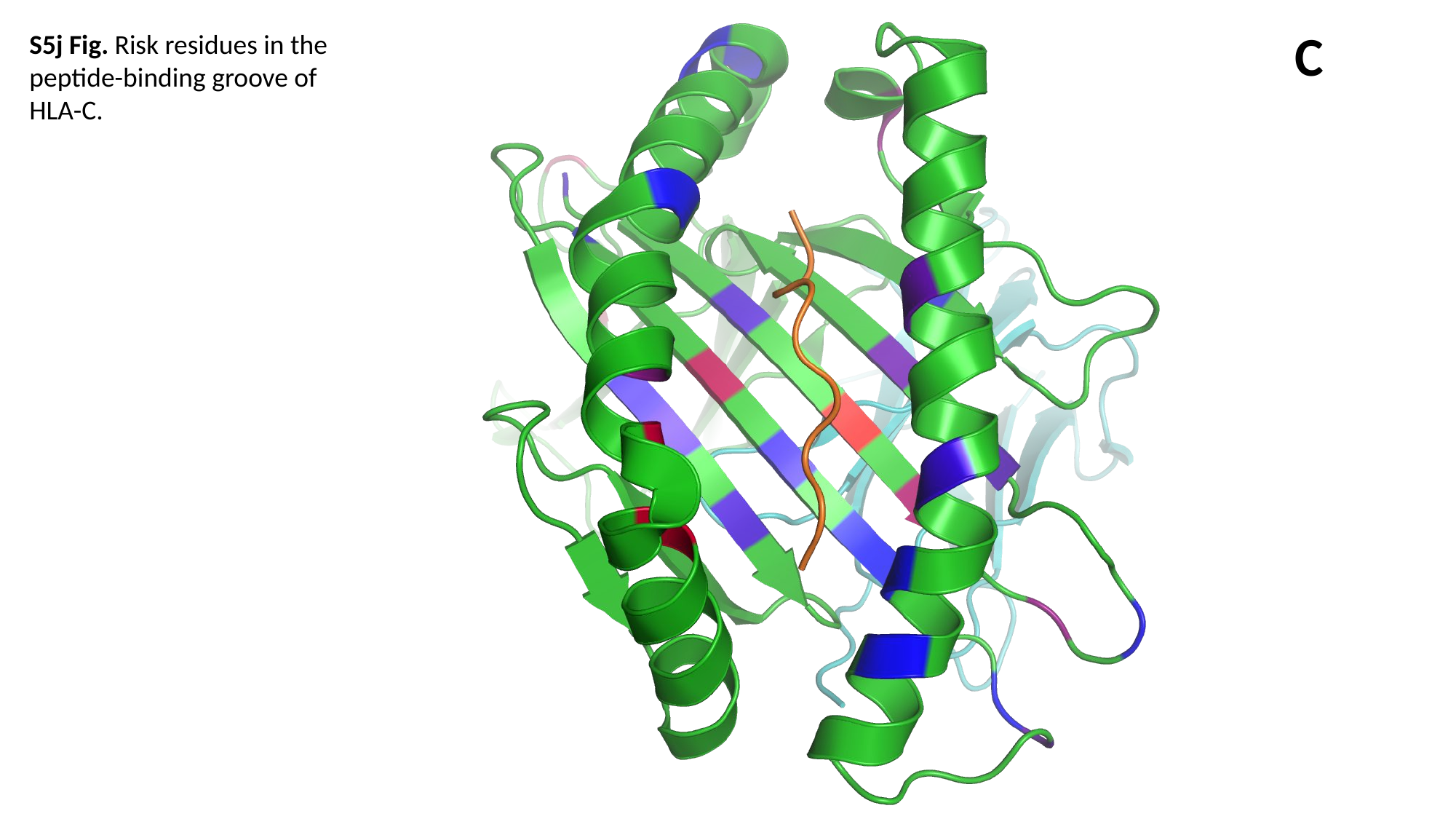

C
S5j Fig. Risk residues in the peptide-binding groove of HLA-C.

## Slide 12
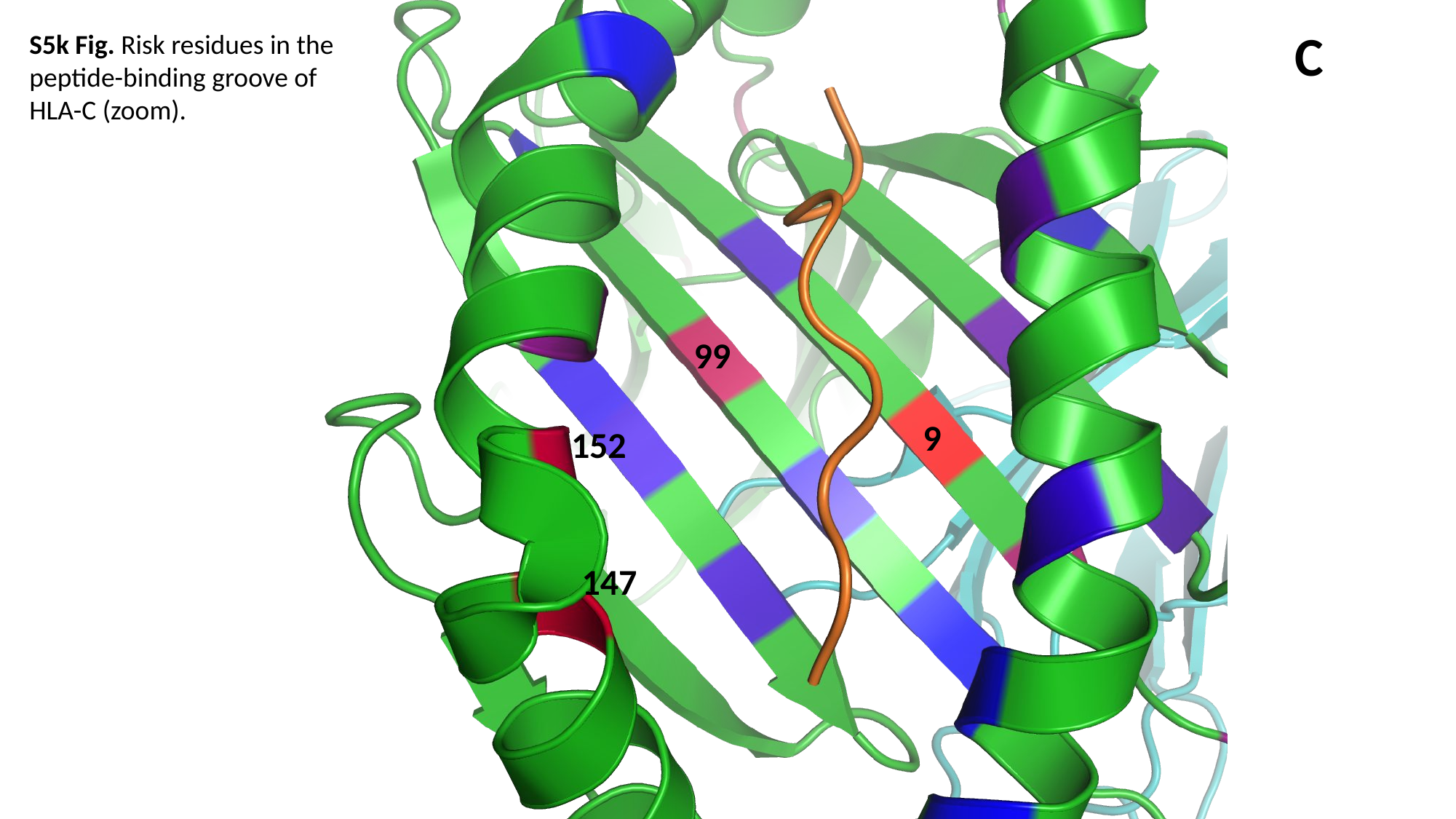

C
S5k Fig. Risk residues in the peptide-binding groove of HLA-C (zoom).
99
9
152
147

## Slide 13
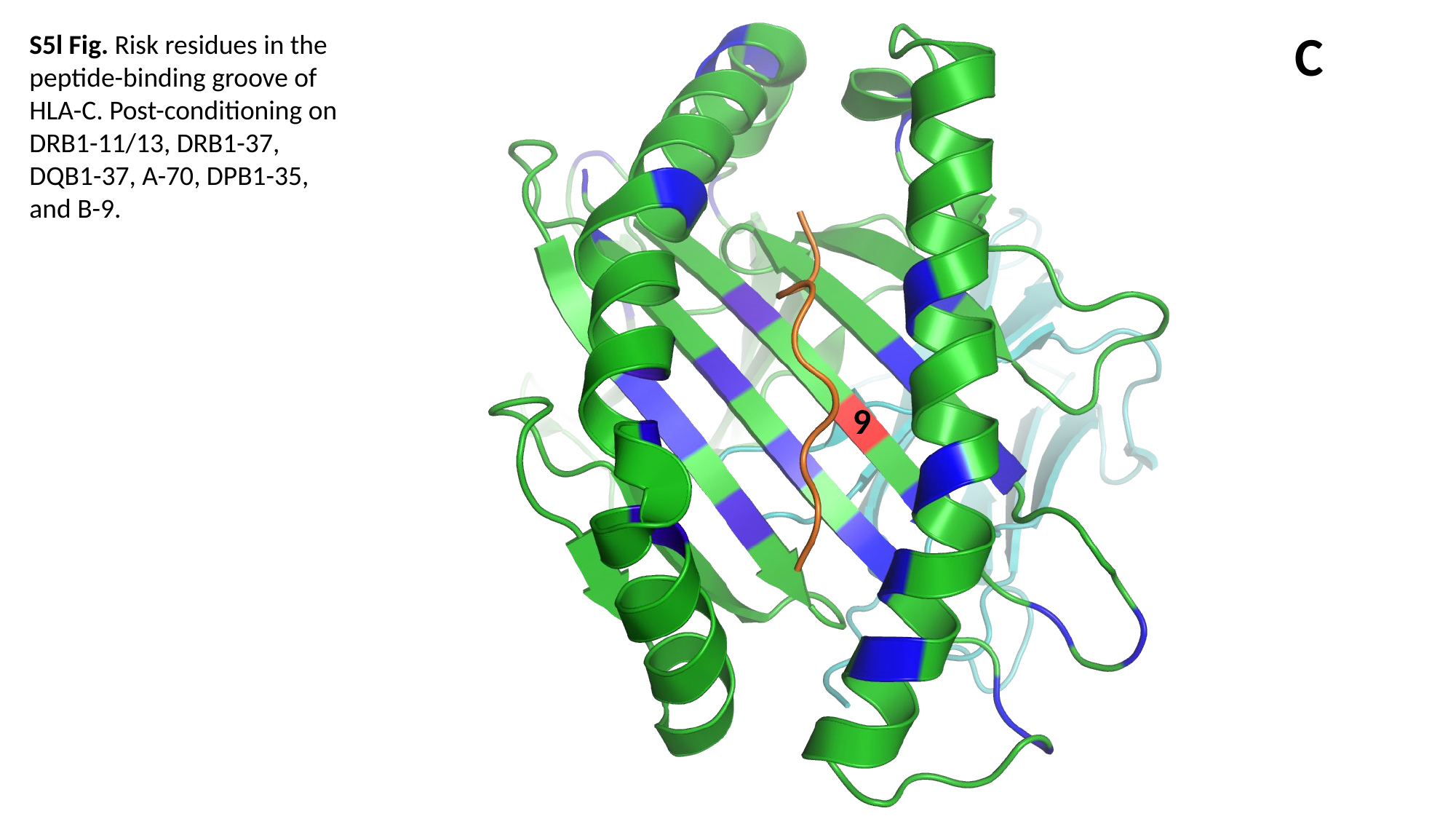

C
S5l Fig. Risk residues in the peptide-binding groove of HLA-C. Post-conditioning on DRB1-11/13, DRB1-37, DQB1-37, A-70, DPB1-35, and B-9.
9
