# Supplementary material for: Amino acid signatures of HLA Class-I and II molecules are strongly associated with SLE susceptibility and autoantibody production in Eastern Asians
Source: PLoS Genet. 2019 Apr 25;15(4):e1008092. doi: 10.1371/journal.pgen.1008092 (PMC6504188; doi:10.1371/journal.pgen.1008092)
Supplement: S6 Fig — A. Accumulation of Class II risk residues in the peptide-binding groove; B. Accumulation of Class II risk residues in the peptide-binding groove (zoom); C Risk residues in the peptide-binding groove of HLA-DPA1/DPB1. Post-conditioning on DRB1-11/13, DRB1-37 and DQB1-37; D. Risk residues in the peptide-binding groove of HLA-DPA1/DPB1. Post-conditioning on DRB1-11/13, DRB1-37 and DQB1-37 (zoom). DPB1-55 emerges as a significantly associated residue in addition to DPB1-35; E. Risk residues in the peptide-binding groove of HLA-DPA1/DPB1. Post-conditioning on DRB1-11/13, DRB1-37, DQB1-37, A-70, DPB1-35, and B-9; F. Risk residues in the peptide-binding groove of HLA-DPA1/DPB1. Post-conditioning on DRB1-11/13, DRB1-37, DQB1-37, A-70, DPB1-35, and B-9 (zoom). DPA1-11 emerges as a significantly associated residue in addition to DPB1-35; G. Risk residues in the peptide-binding groove of HLA-DQA1/DQB1. Post-conditioning on DRB1-11/13 and DRB1-37; H. Risk residues in the peptide-binding groove of HLA-DQA1/DQB1. Post-conditioning on DRB1-11/13 and DRB1-37 (zoom). DQB1-70 emerges as a significantly associated residue in addition to DQB1-37; I. Risk residues in the peptide-binding groove of HLA-DQA1/DQB1. Post-conditioning on DRB1-11/13, DRB1-37, DQB1-37, A-70, DPB1-35, and B-9; J. Risk residues in the peptide-binding groove of HLA-DQA1/DQB1. Post-conditioning on DRB1-11/13, DRB1-37, DQB1-37, A-70, DPB1-35, and B-9 (zoom). DQB1-57 emerges as a significantly associated residue in addition to DQB1-37; K. Risk residues in the peptide-binding groove of HLA-DRA1/DRB1; L. Risk residues in the peptide-binding groove of HLA-DRA1/DRB1 (zoom); M. Risk residues in the peptide-binding groove of HLA-DRA1/DRB1. Post-conditioning on DRB1-11/13; N. Risk residues in the peptide-binding groove of HLA-DRA1/DRB1. Post-conditioning on DRB1-11/13, DRB1-37, DQB1-37, A-70, DPB1-35, and B-9. DRB1-67 emerges as a significantly associated residue; O. Correspondence of risk positions across multiple Class I [file pgen.1008092.s006.pptx]

## Slide 1
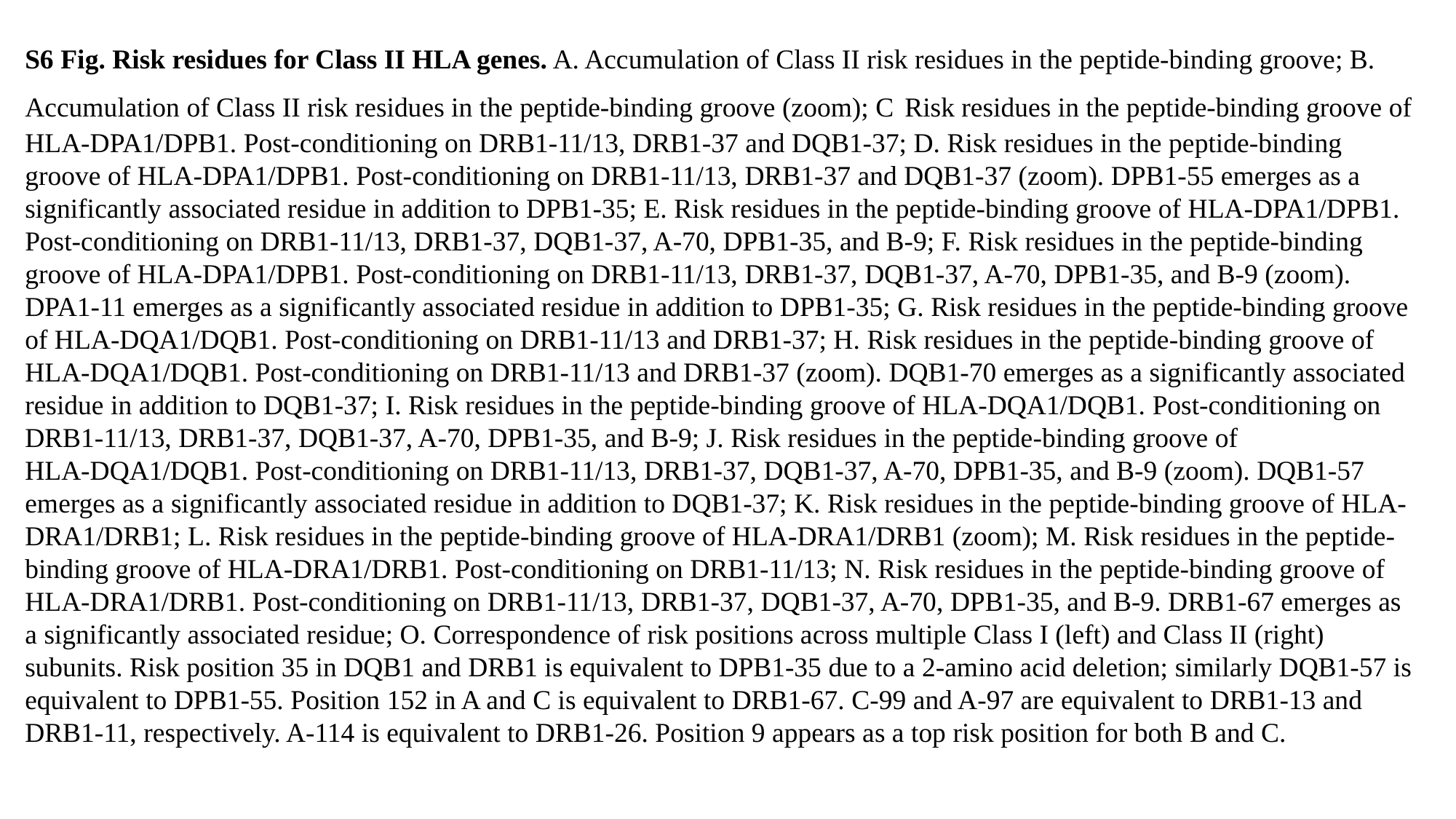

S6 Fig. Risk residues for Class II HLA genes. A. Accumulation of Class II risk residues in the peptide-binding groove; B. Accumulation of Class II risk residues in the peptide-binding groove (zoom); C Risk residues in the peptide-binding groove of HLA-DPA1/DPB1. Post-conditioning on DRB1-11/13, DRB1-37 and DQB1-37; D. Risk residues in the peptide-binding groove of HLA-DPA1/DPB1. Post-conditioning on DRB1-11/13, DRB1-37 and DQB1-37 (zoom). DPB1-55 emerges as a significantly associated residue in addition to DPB1-35; E. Risk residues in the peptide-binding groove of HLA-DPA1/DPB1. Post-conditioning on DRB1-11/13, DRB1-37, DQB1-37, A-70, DPB1-35, and B-9; F. Risk residues in the peptide-binding groove of HLA-DPA1/DPB1. Post-conditioning on DRB1-11/13, DRB1-37, DQB1-37, A-70, DPB1-35, and B-9 (zoom). DPA1-11 emerges as a significantly associated residue in addition to DPB1-35; G. Risk residues in the peptide-binding groove of HLA-DQA1/DQB1. Post-conditioning on DRB1-11/13 and DRB1-37; H. Risk residues in the peptide-binding groove of HLA-DQA1/DQB1. Post-conditioning on DRB1-11/13 and DRB1-37 (zoom). DQB1-70 emerges as a significantly associated residue in addition to DQB1-37; I. Risk residues in the peptide-binding groove of HLA-DQA1/DQB1. Post-conditioning on DRB1-11/13, DRB1-37, DQB1-37, A-70, DPB1-35, and B-9; J. Risk residues in the peptide-binding groove of HLA-DQA1/DQB1. Post-conditioning on DRB1-11/13, DRB1-37, DQB1-37, A-70, DPB1-35, and B-9 (zoom). DQB1-57 emerges as a significantly associated residue in addition to DQB1-37; K. Risk residues in the peptide-binding groove of HLA-DRA1/DRB1; L. Risk residues in the peptide-binding groove of HLA-DRA1/DRB1 (zoom); M. Risk residues in the peptide-binding groove of HLA-DRA1/DRB1. Post-conditioning on DRB1-11/13; N. Risk residues in the peptide-binding groove of HLA-DRA1/DRB1. Post-conditioning on DRB1-11/13, DRB1-37, DQB1-37, A-70, DPB1-35, and B-9. DRB1-67 emerges as a significantly associated residue; O. Correspondence of risk positions across multiple Class I (left) and Class II (right) subunits. Risk position 35 in DQB1 and DRB1 is equivalent to DPB1-35 due to a 2-amino acid deletion; similarly DQB1-57 is equivalent to DPB1-55. Position 152 in A and C is equivalent to DRB1-67. C-99 and A-97 are equivalent to DRB1-13 and DRB1-11, respectively. A-114 is equivalent to DRB1-26. Position 9 appears as a top risk position for both B and C.

## Slide 2
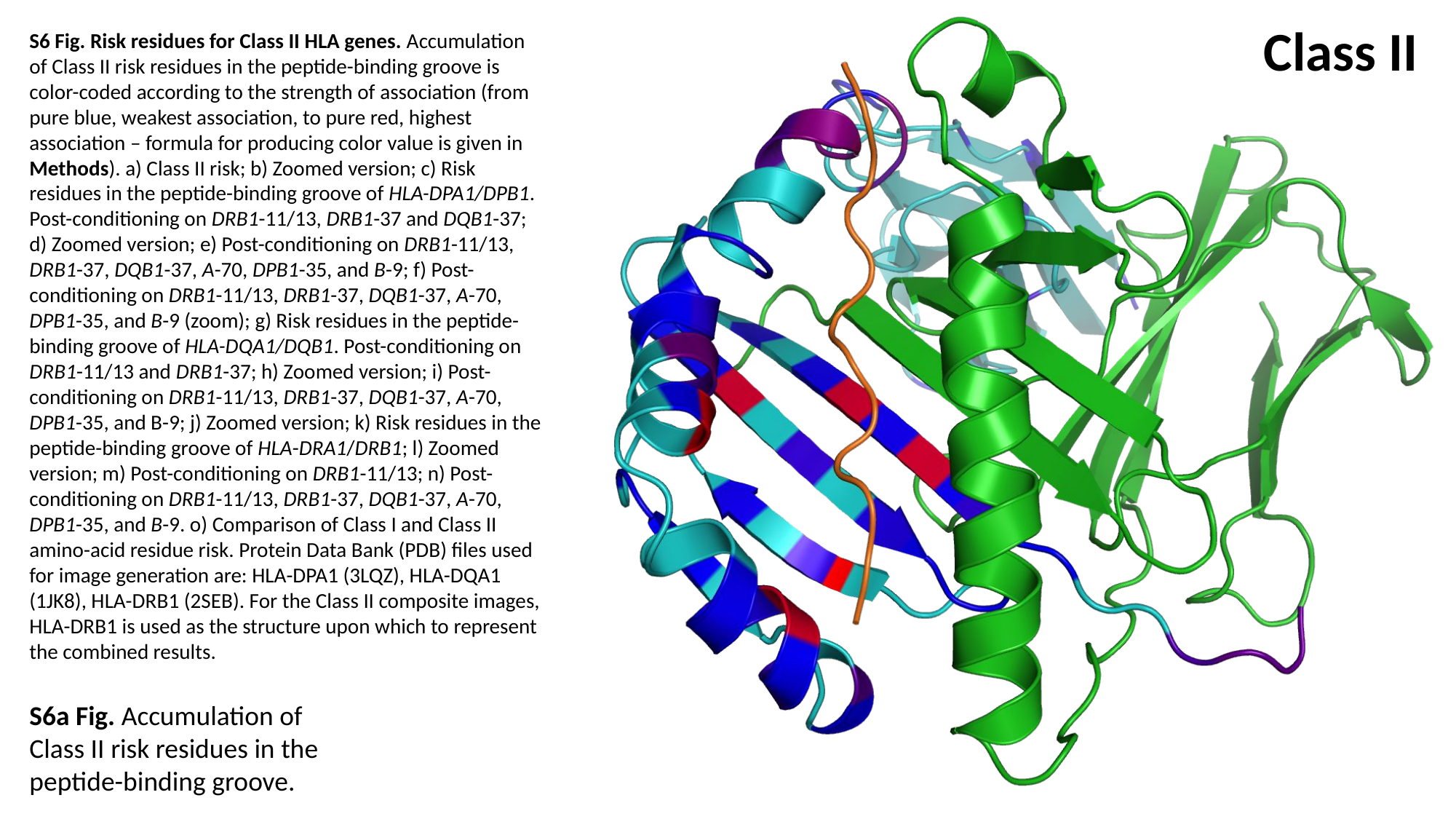

Class II
S6 Fig. Risk residues for Class II HLA genes. Accumulation of Class II risk residues in the peptide-binding groove is color-coded according to the strength of association (from pure blue, weakest association, to pure red, highest association – formula for producing color value is given in Methods). a) Class II risk; b) Zoomed version; c) Risk residues in the peptide-binding groove of HLA-DPA1/DPB1. Post-conditioning on DRB1-11/13, DRB1-37 and DQB1-37; d) Zoomed version; e) Post-conditioning on DRB1-11/13, DRB1-37, DQB1-37, A-70, DPB1-35, and B-9; f) Post-conditioning on DRB1-11/13, DRB1-37, DQB1-37, A-70, DPB1-35, and B-9 (zoom); g) Risk residues in the peptide-binding groove of HLA-DQA1/DQB1. Post-conditioning on DRB1-11/13 and DRB1-37; h) Zoomed version; i) Post-conditioning on DRB1-11/13, DRB1-37, DQB1-37, A-70, DPB1-35, and B-9; j) Zoomed version; k) Risk residues in the peptide-binding groove of HLA-DRA1/DRB1; l) Zoomed version; m) Post-conditioning on DRB1-11/13; n) Post-conditioning on DRB1-11/13, DRB1-37, DQB1-37, A-70, DPB1-35, and B-9. o) Comparison of Class I and Class II amino-acid residue risk. Protein Data Bank (PDB) files used for image generation are: HLA-DPA1 (3LQZ), HLA-DQA1 (1JK8), HLA-DRB1 (2SEB). For the Class II composite images, HLA-DRB1 is used as the structure upon which to represent the combined results.
S6a Fig. Accumulation of Class II risk residues in the peptide-binding groove.

## Slide 3
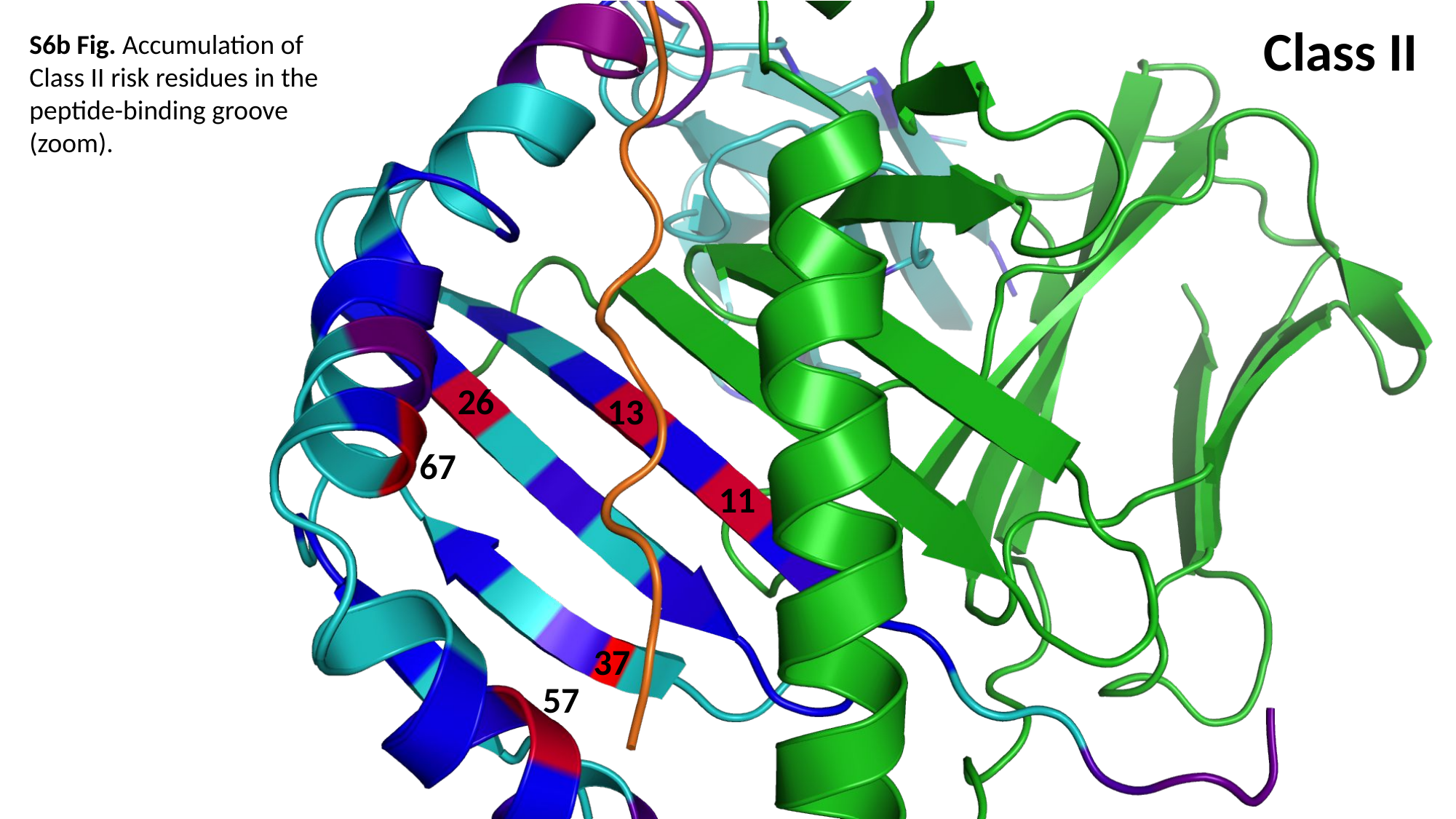

Class II
S6b Fig. Accumulation of Class II risk residues in the peptide-binding groove (zoom).
26
13
67
11
37
57

## Slide 4
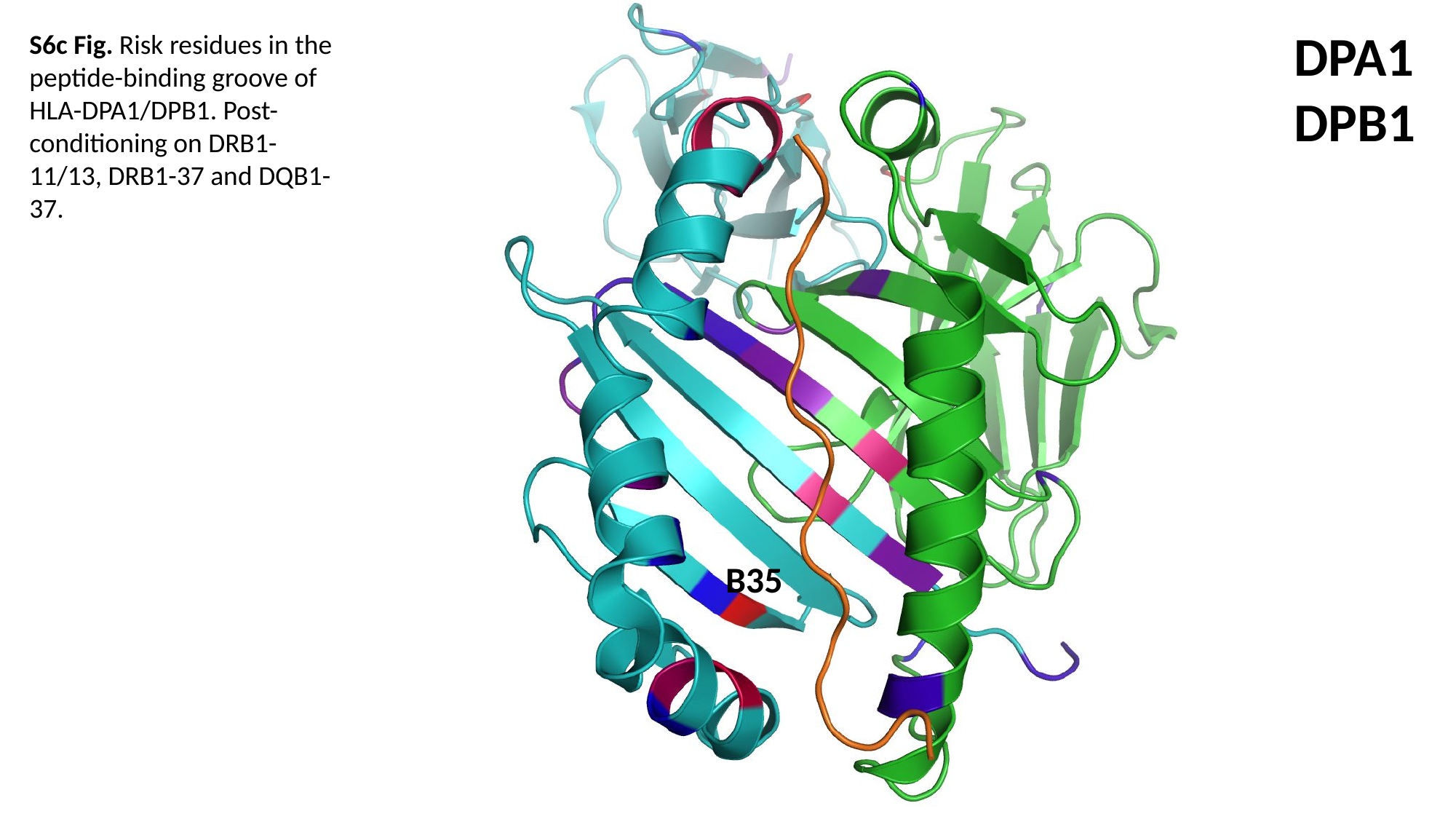

DPA1
DPB1
S6c Fig. Risk residues in the peptide-binding groove of HLA-DPA1/DPB1. Post-conditioning on DRB1-11/13, DRB1-37 and DQB1-37.
B35

## Slide 5
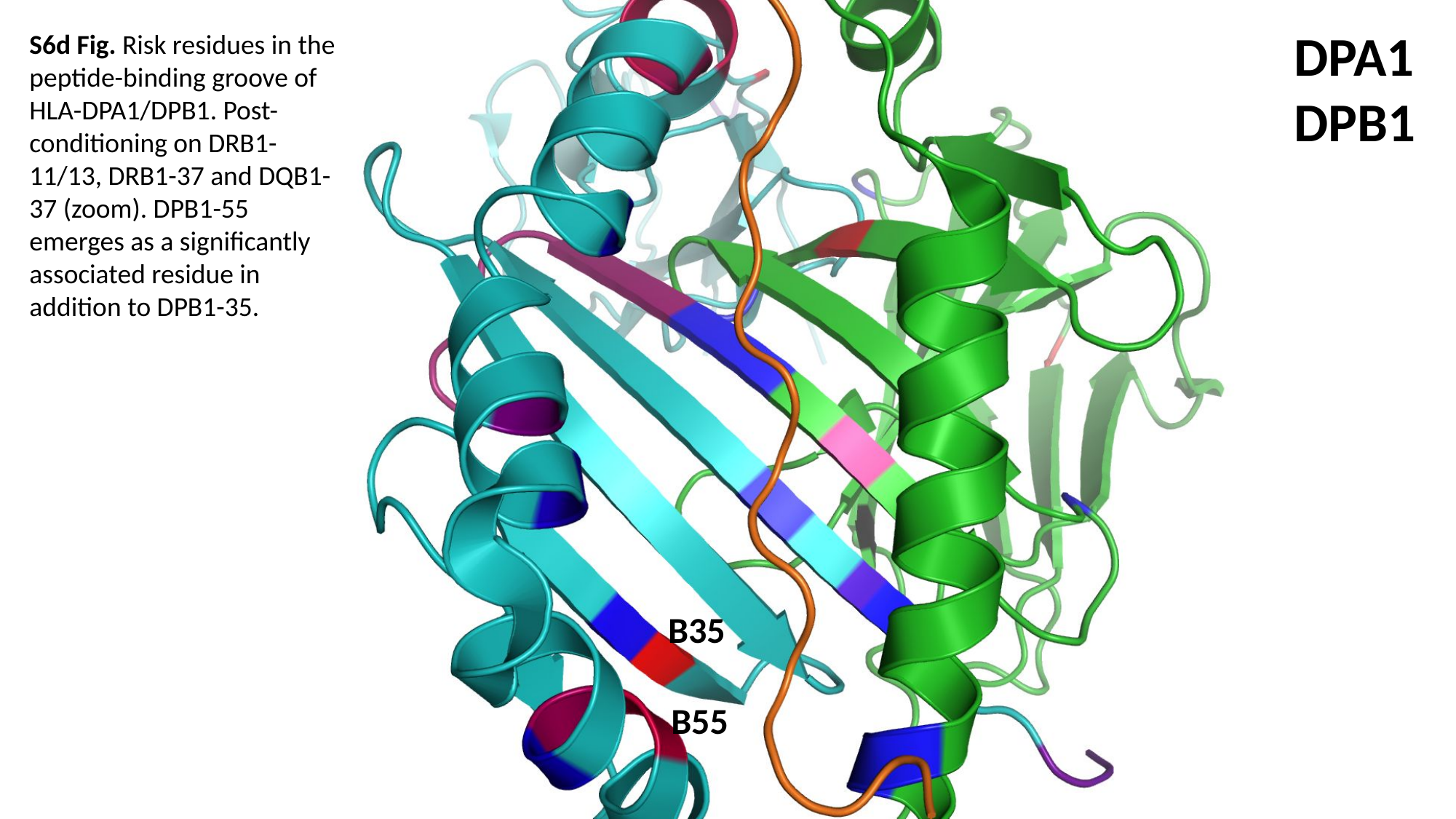

DPA1
DPB1
S6d Fig. Risk residues in the peptide-binding groove of HLA-DPA1/DPB1. Post-conditioning on DRB1-11/13, DRB1-37 and DQB1-37 (zoom). DPB1-55 emerges as a significantly associated residue in addition to DPB1-35.
B35
B55

## Slide 6
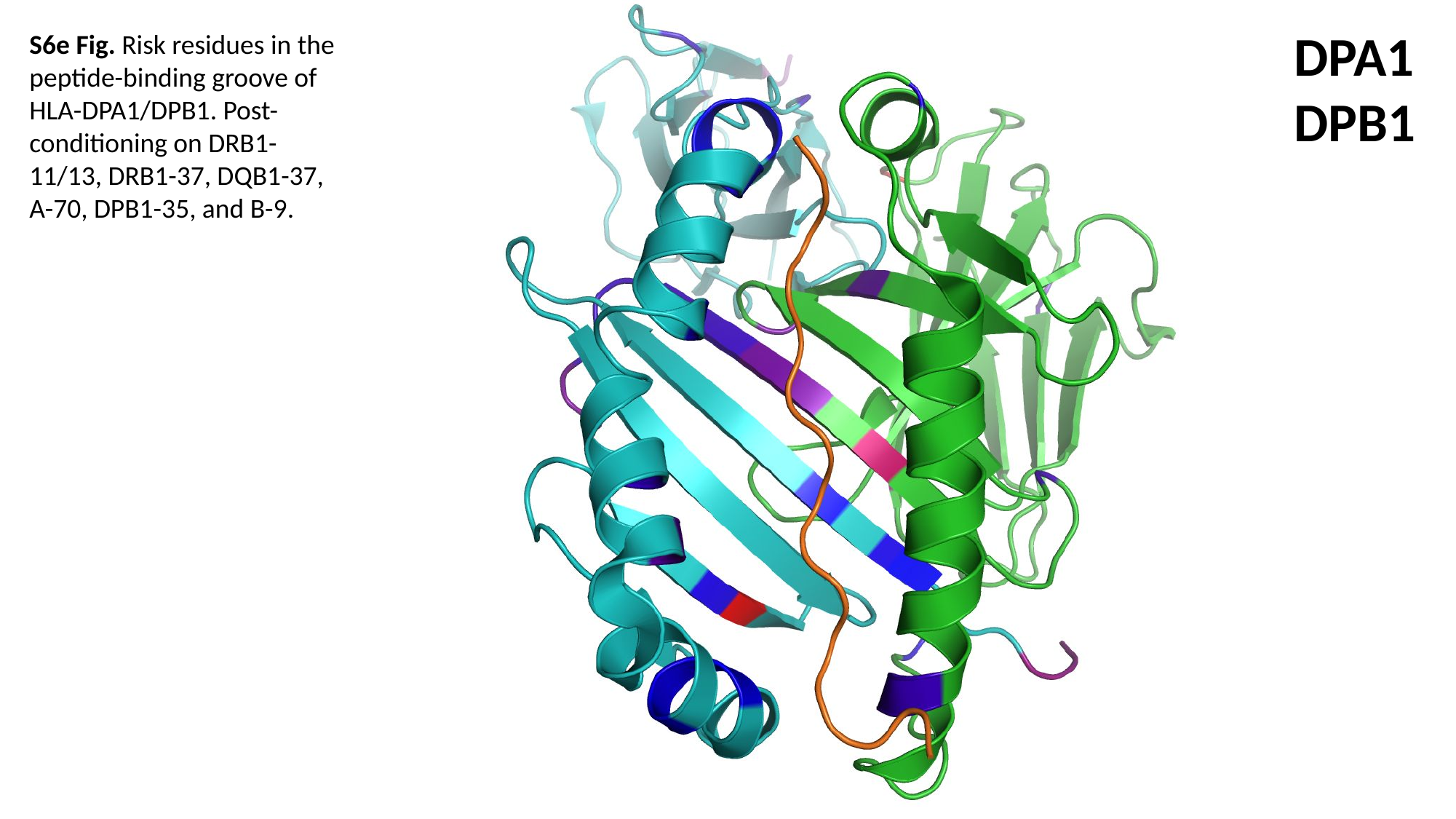

DPA1
DPB1
S6e Fig. Risk residues in the peptide-binding groove of HLA-DPA1/DPB1. Post-conditioning on DRB1-11/13, DRB1-37, DQB1-37, A-70, DPB1-35, and B-9.

## Slide 7
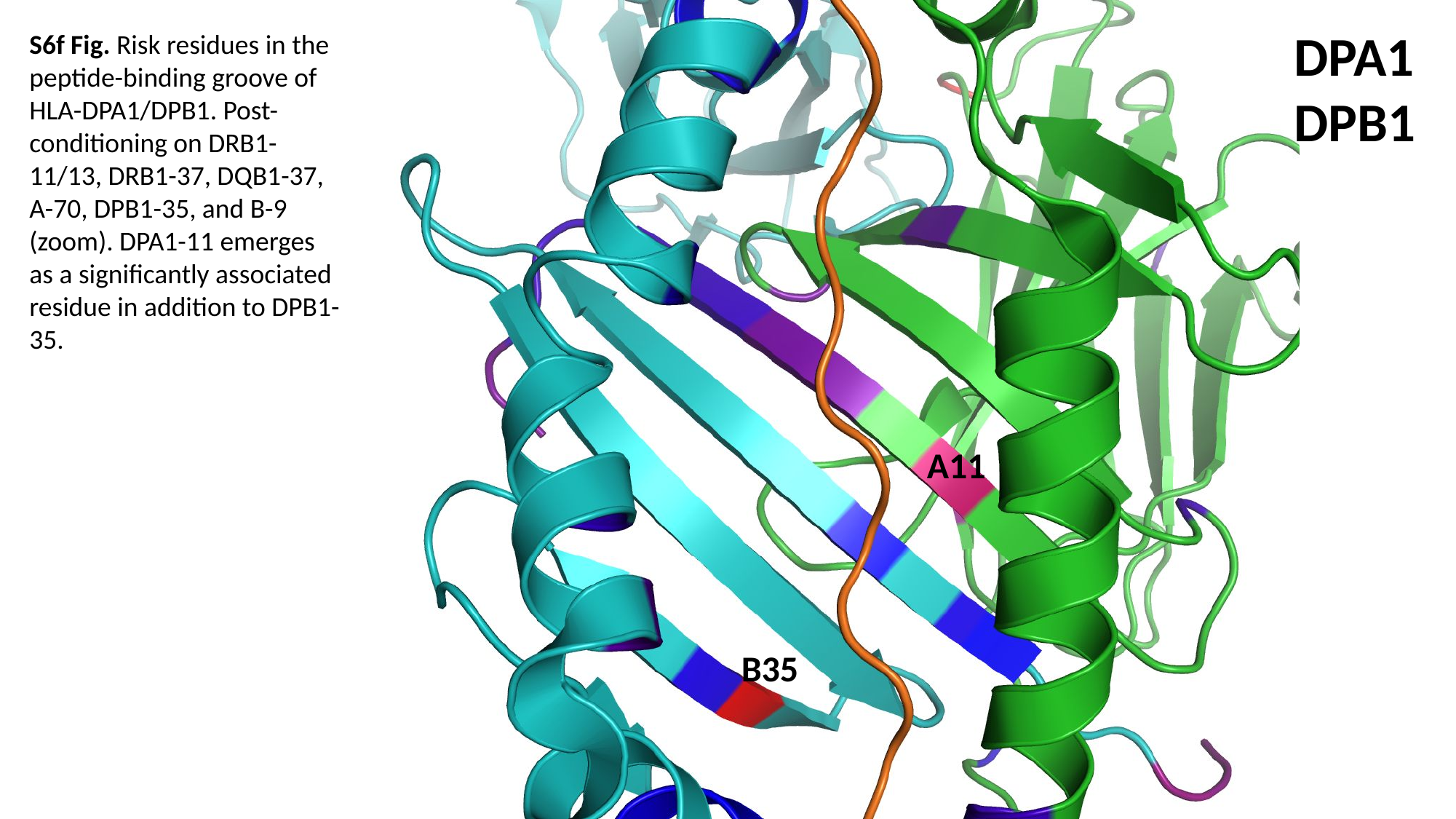

DPA1
DPB1
S6f Fig. Risk residues in the peptide-binding groove of HLA-DPA1/DPB1. Post-conditioning on DRB1-11/13, DRB1-37, DQB1-37, A-70, DPB1-35, and B-9 (zoom). DPA1-11 emerges as a significantly associated residue in addition to DPB1-35.
A11
B35

## Slide 8
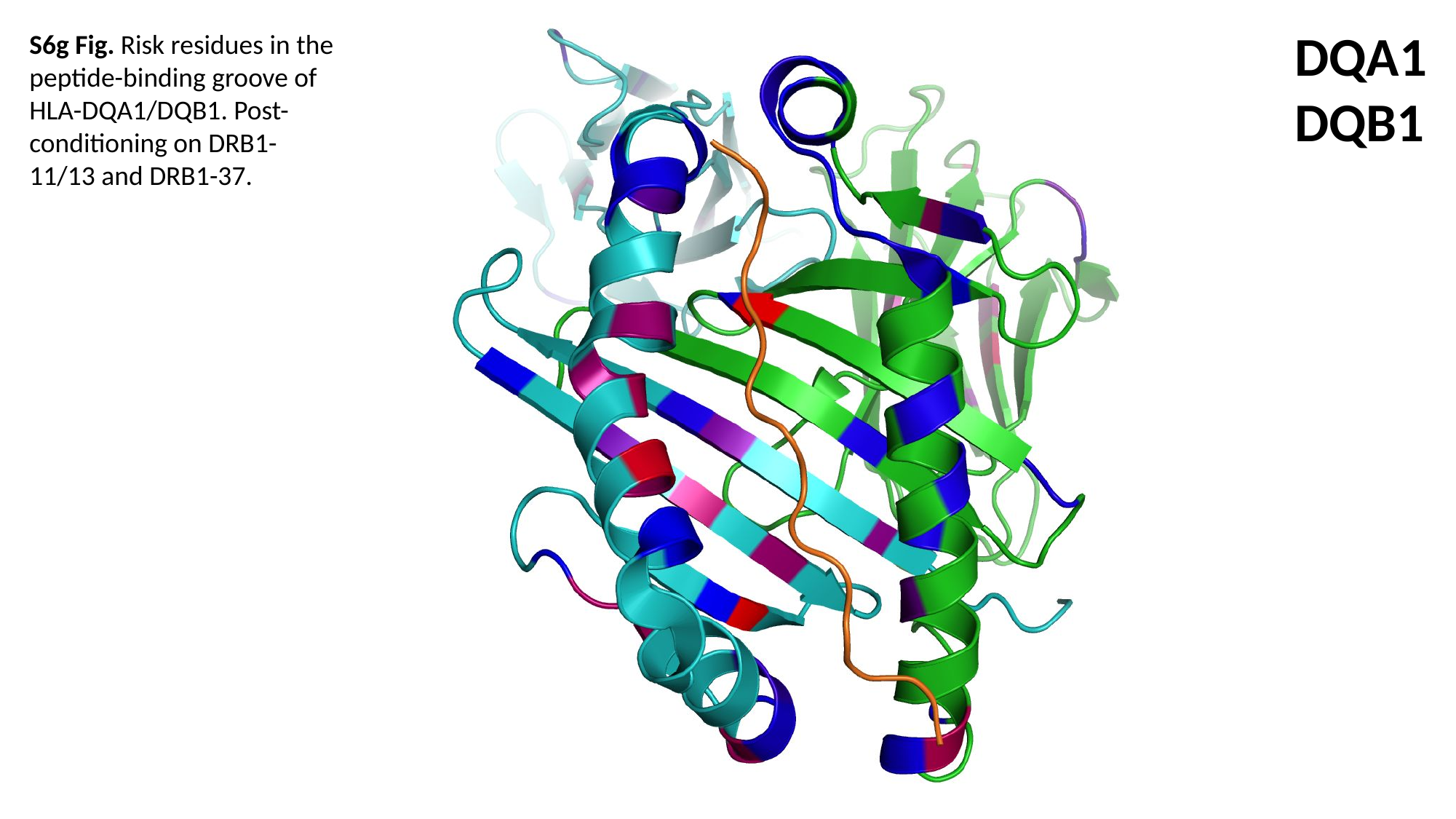

DQA1
DQB1
S6g Fig. Risk residues in the peptide-binding groove of HLA-DQA1/DQB1. Post-conditioning on DRB1-11/13 and DRB1-37.

## Slide 9
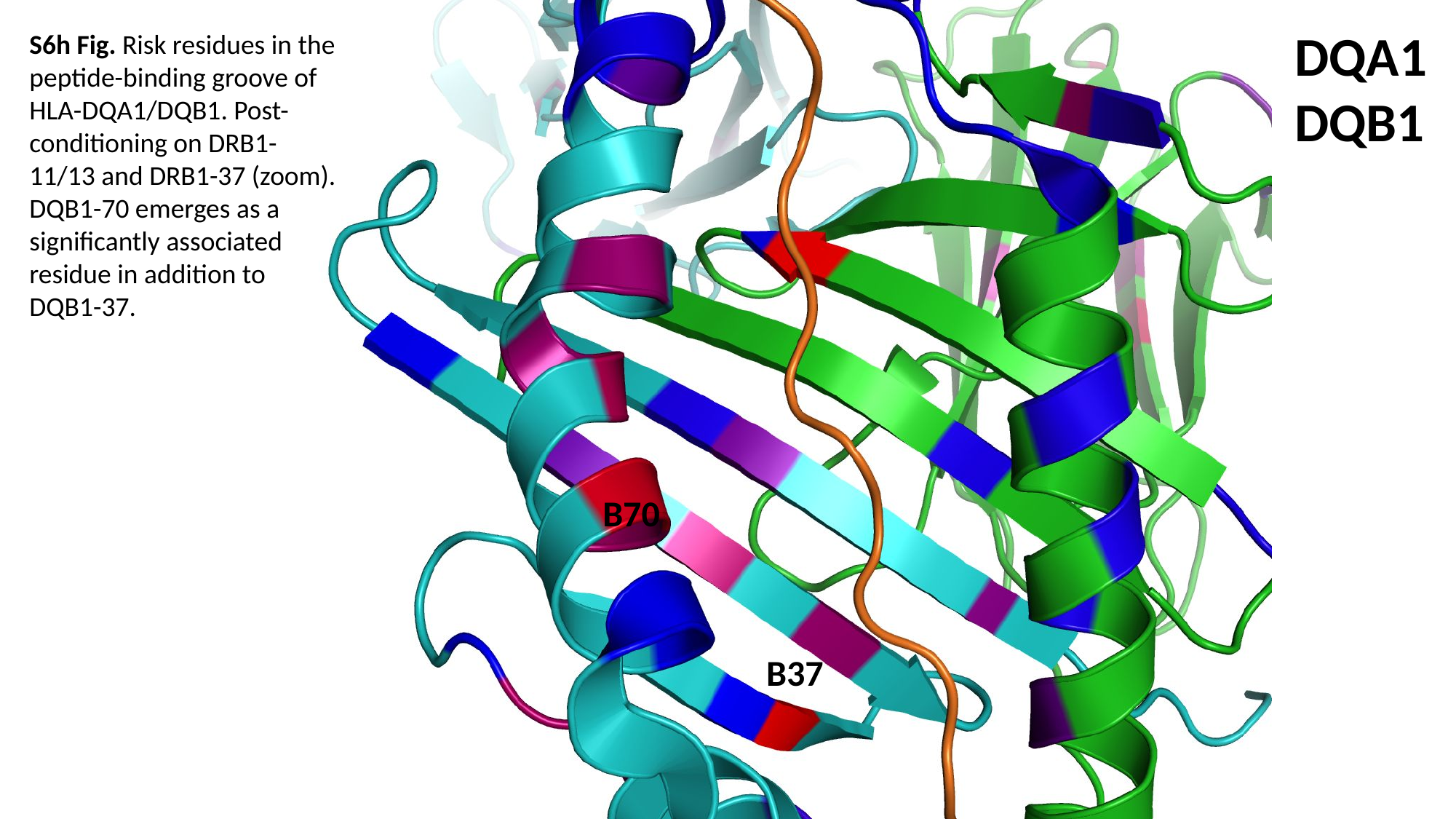

DQA1
DQB1
S6h Fig. Risk residues in the peptide-binding groove of HLA-DQA1/DQB1. Post-conditioning on DRB1-11/13 and DRB1-37 (zoom). DQB1-70 emerges as a significantly associated residue in addition to DQB1-37.
B70
B37

## Slide 10
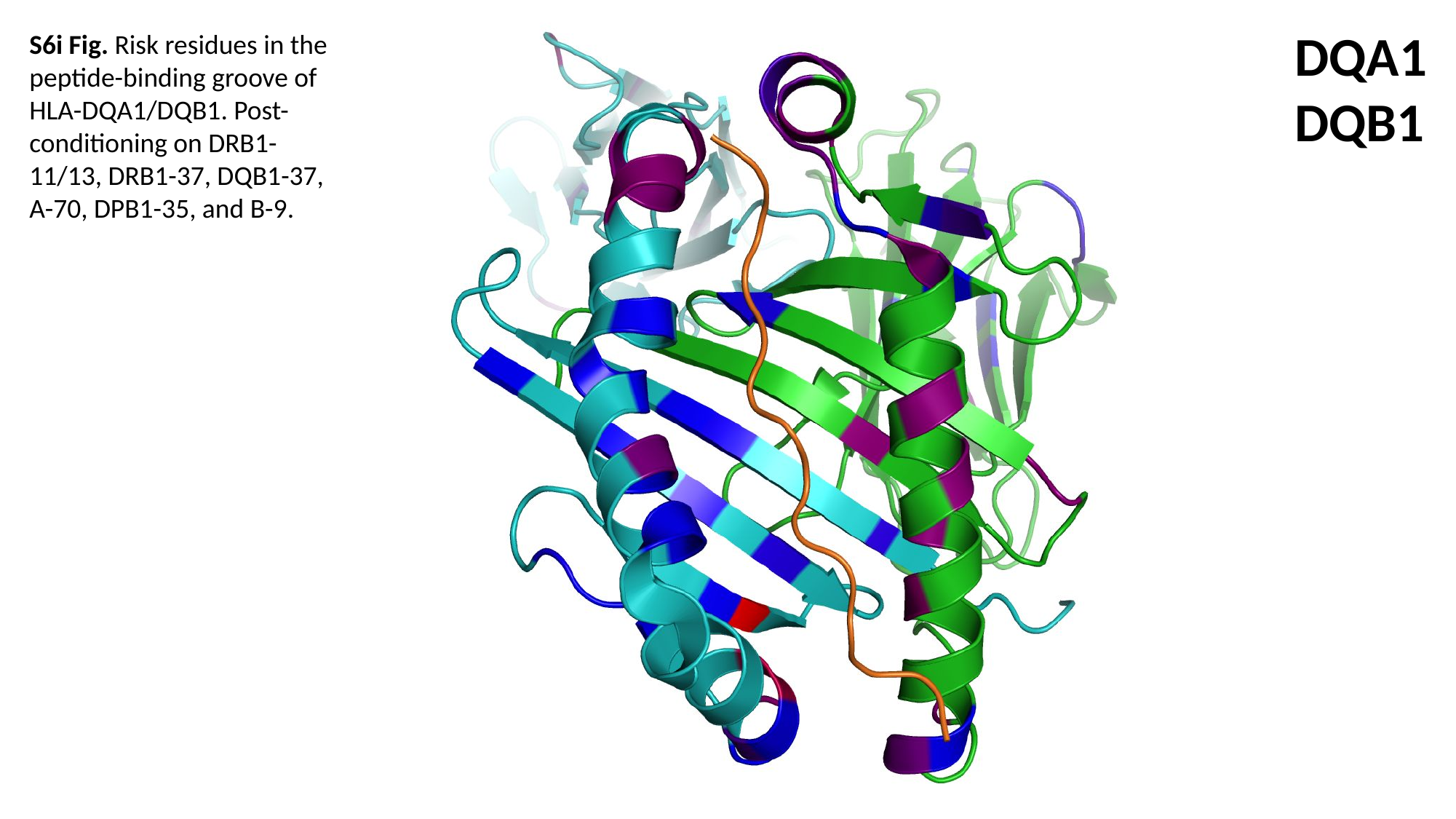

DQA1
DQB1
S6i Fig. Risk residues in the peptide-binding groove of HLA-DQA1/DQB1. Post-conditioning on DRB1-11/13, DRB1-37, DQB1-37, A-70, DPB1-35, and B-9.

## Slide 11
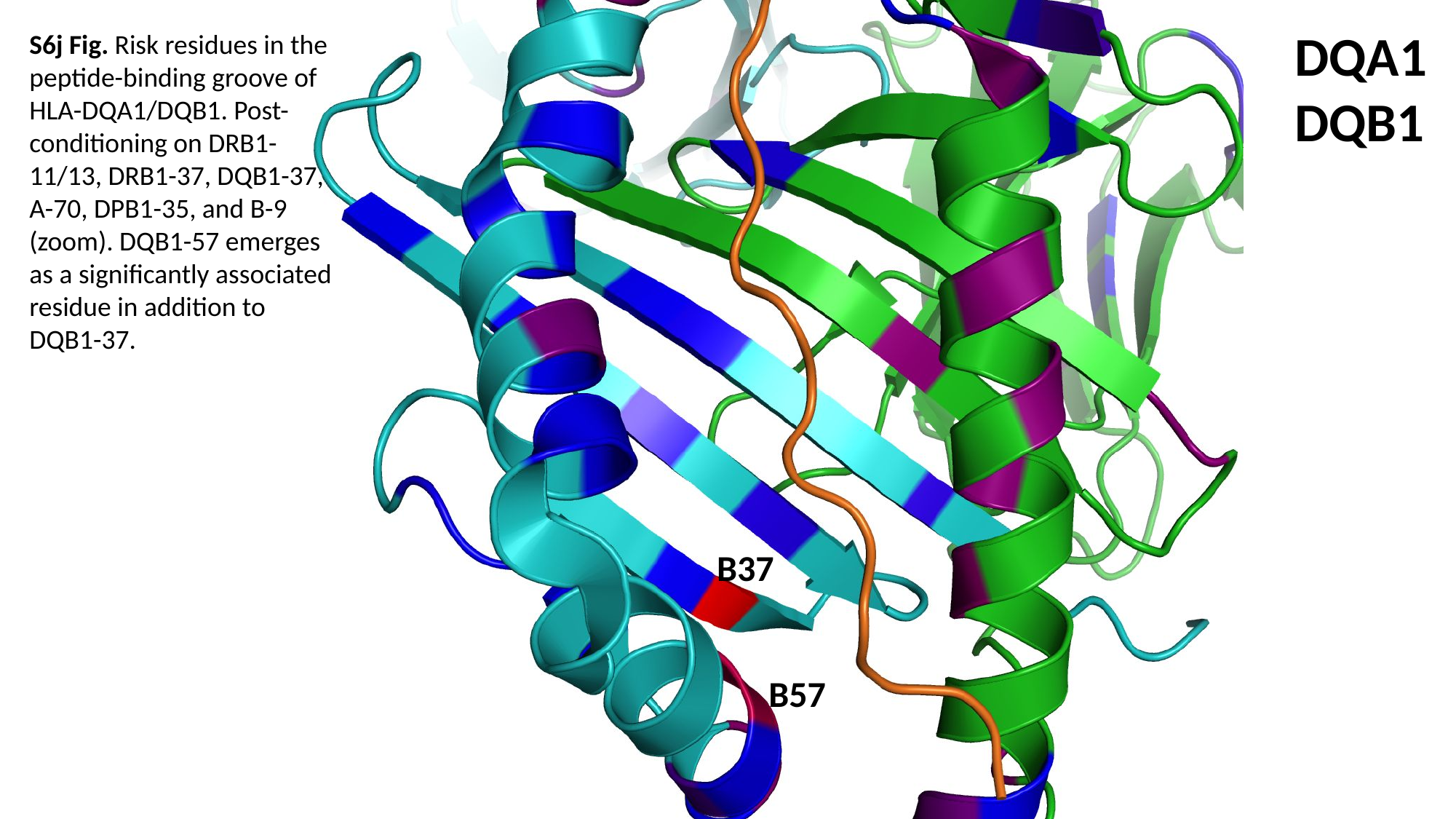

DQA1
DQB1
S6j Fig. Risk residues in the peptide-binding groove of HLA-DQA1/DQB1. Post-conditioning on DRB1-11/13, DRB1-37, DQB1-37, A-70, DPB1-35, and B-9 (zoom). DQB1-57 emerges as a significantly associated residue in addition to DQB1-37.
B37
B57

## Slide 12
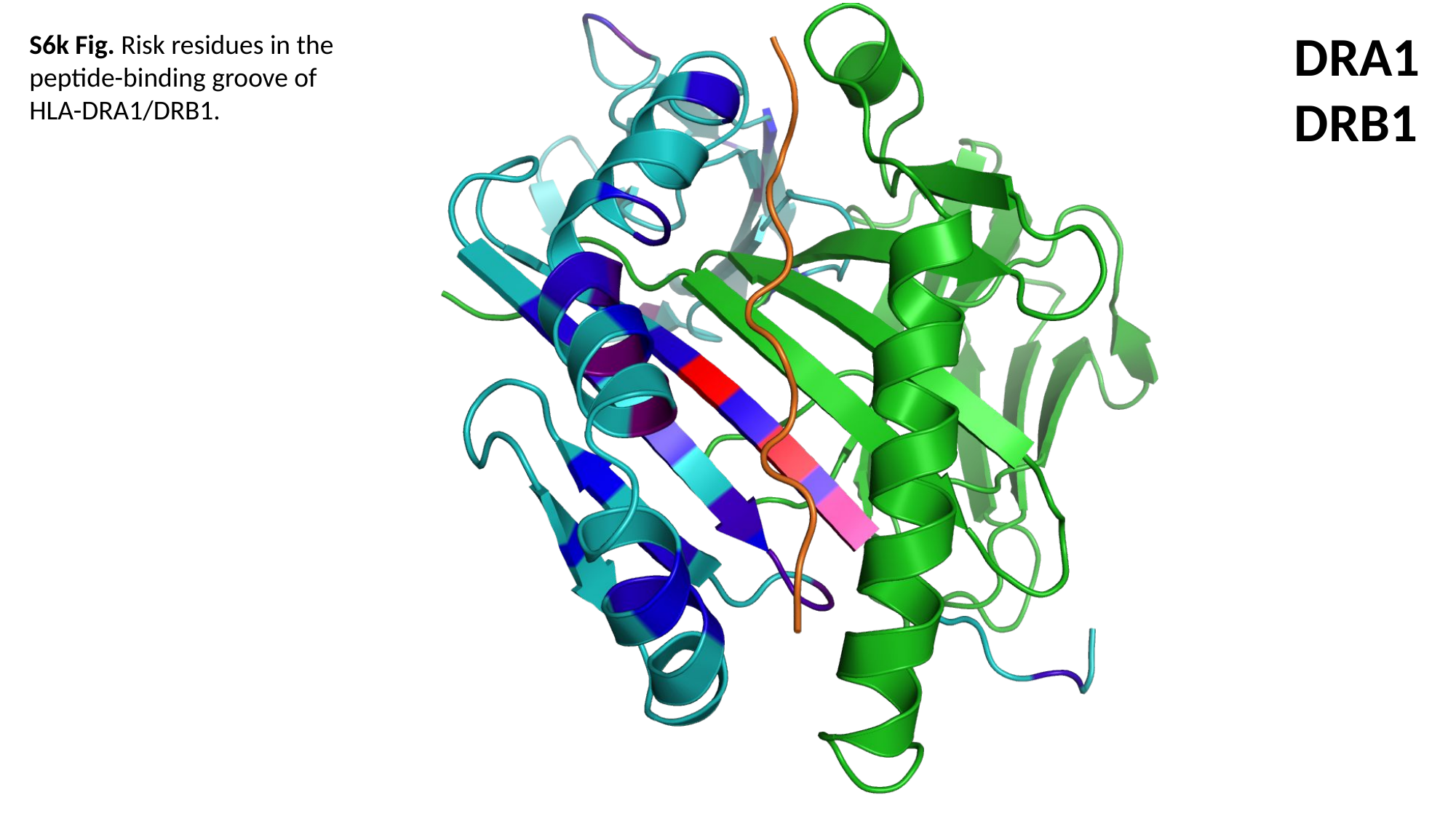

DRA1
DRB1
S6k Fig. Risk residues in the peptide-binding groove of HLA-DRA1/DRB1.

## Slide 13
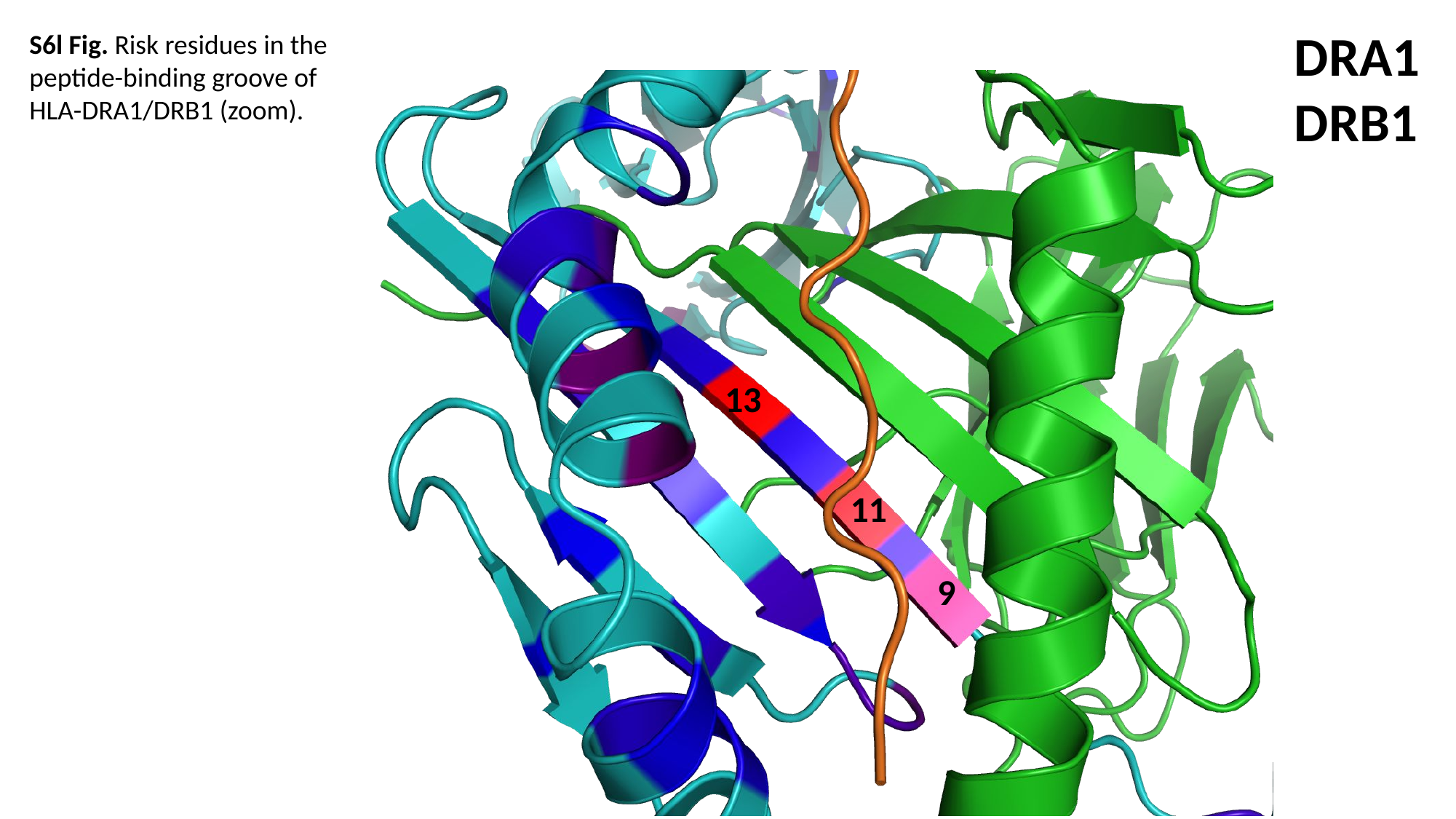

DRA1
DRB1
S6l Fig. Risk residues in the peptide-binding groove of HLA-DRA1/DRB1 (zoom).
13
11
9

## Slide 14
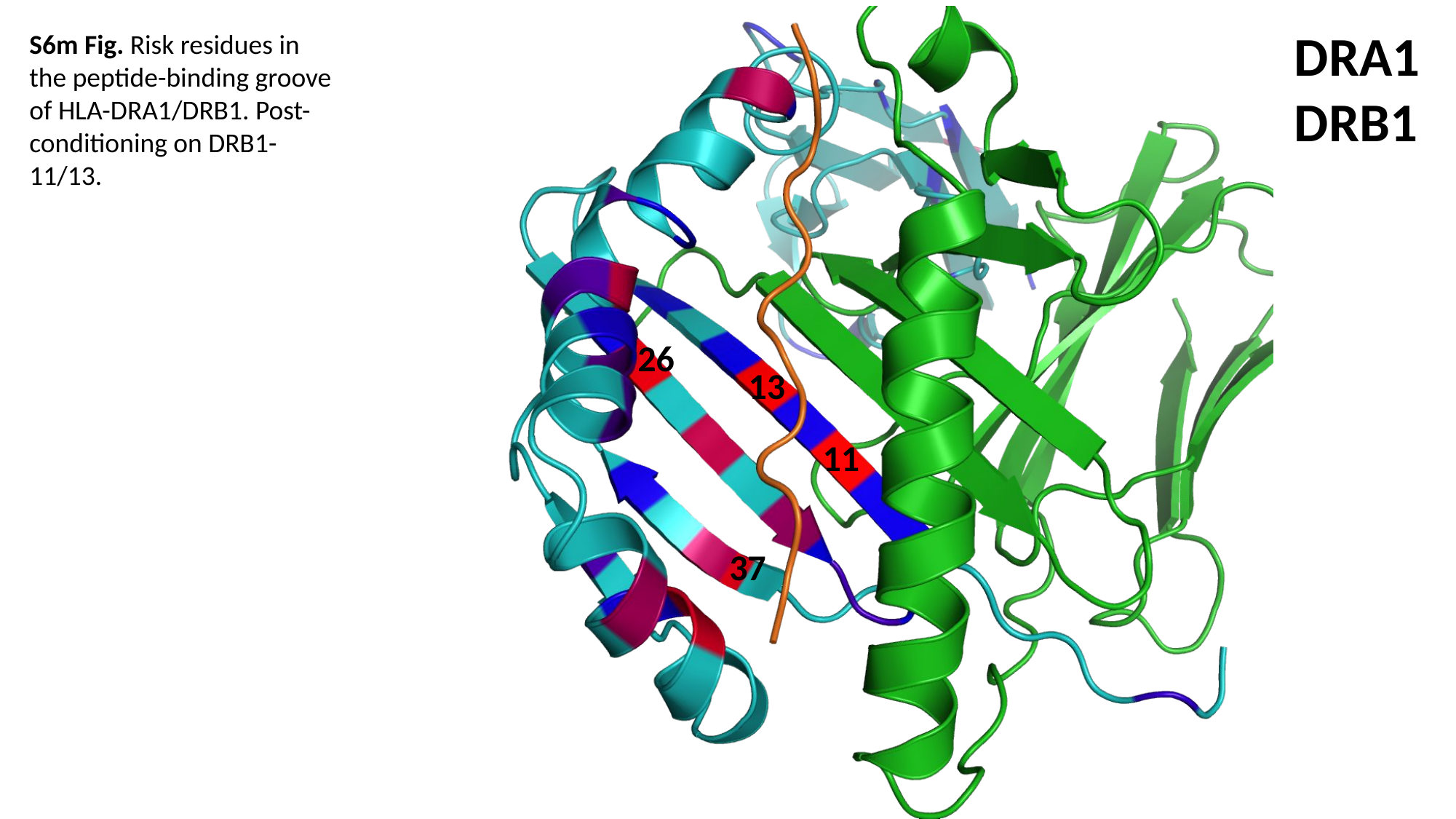

DRA1
DRB1
S6m Fig. Risk residues in the peptide-binding groove of HLA-DRA1/DRB1. Post-conditioning on DRB1-11/13.
26
13
11
37

## Slide 15
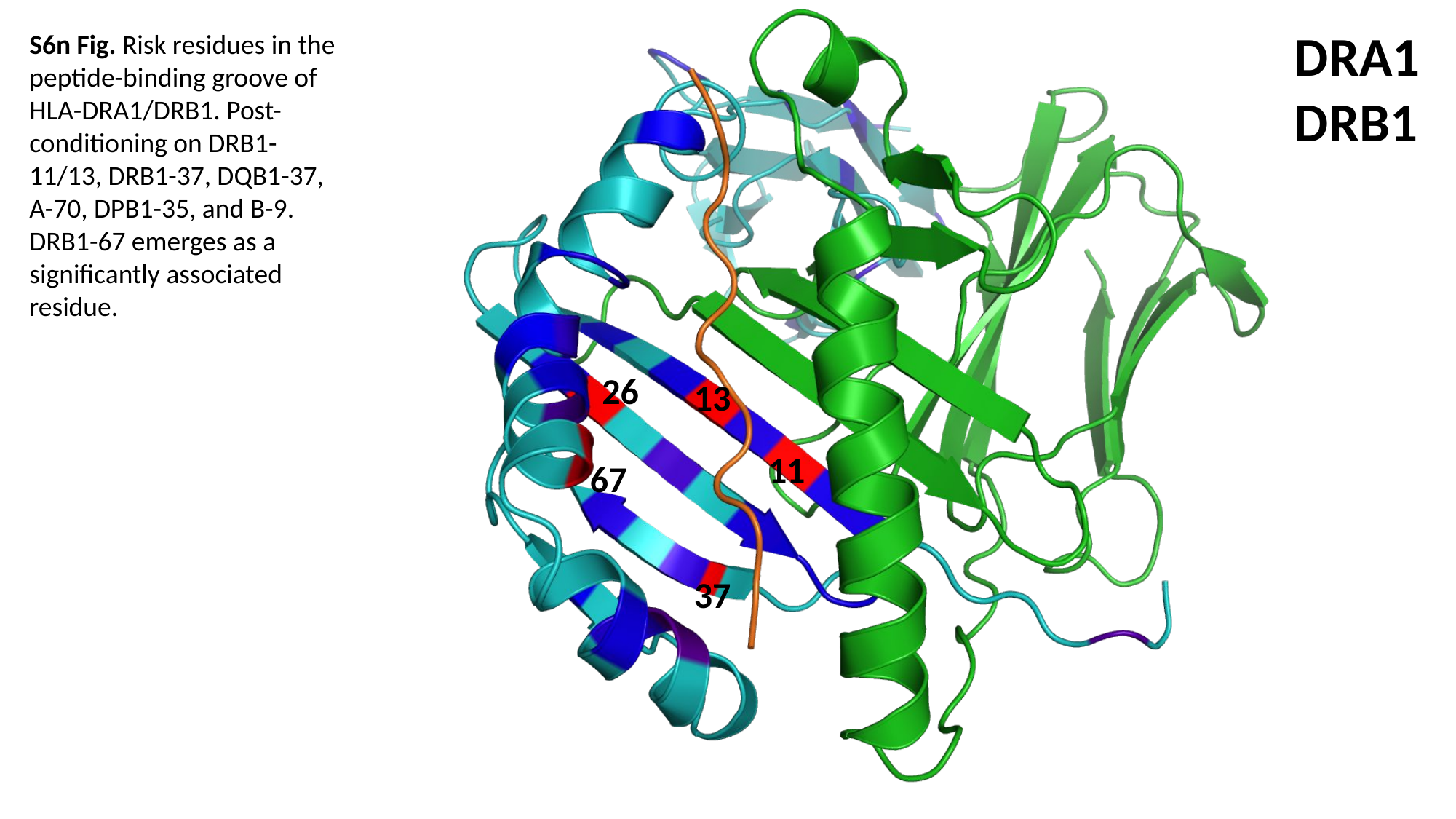

DRA1
DRB1
S6n Fig. Risk residues in the peptide-binding groove of HLA-DRA1/DRB1. Post-conditioning on DRB1-11/13, DRB1-37, DQB1-37, A-70, DPB1-35, and B-9. DRB1-67 emerges as a significantly associated residue.
26
13
11
67
37

## Slide 16
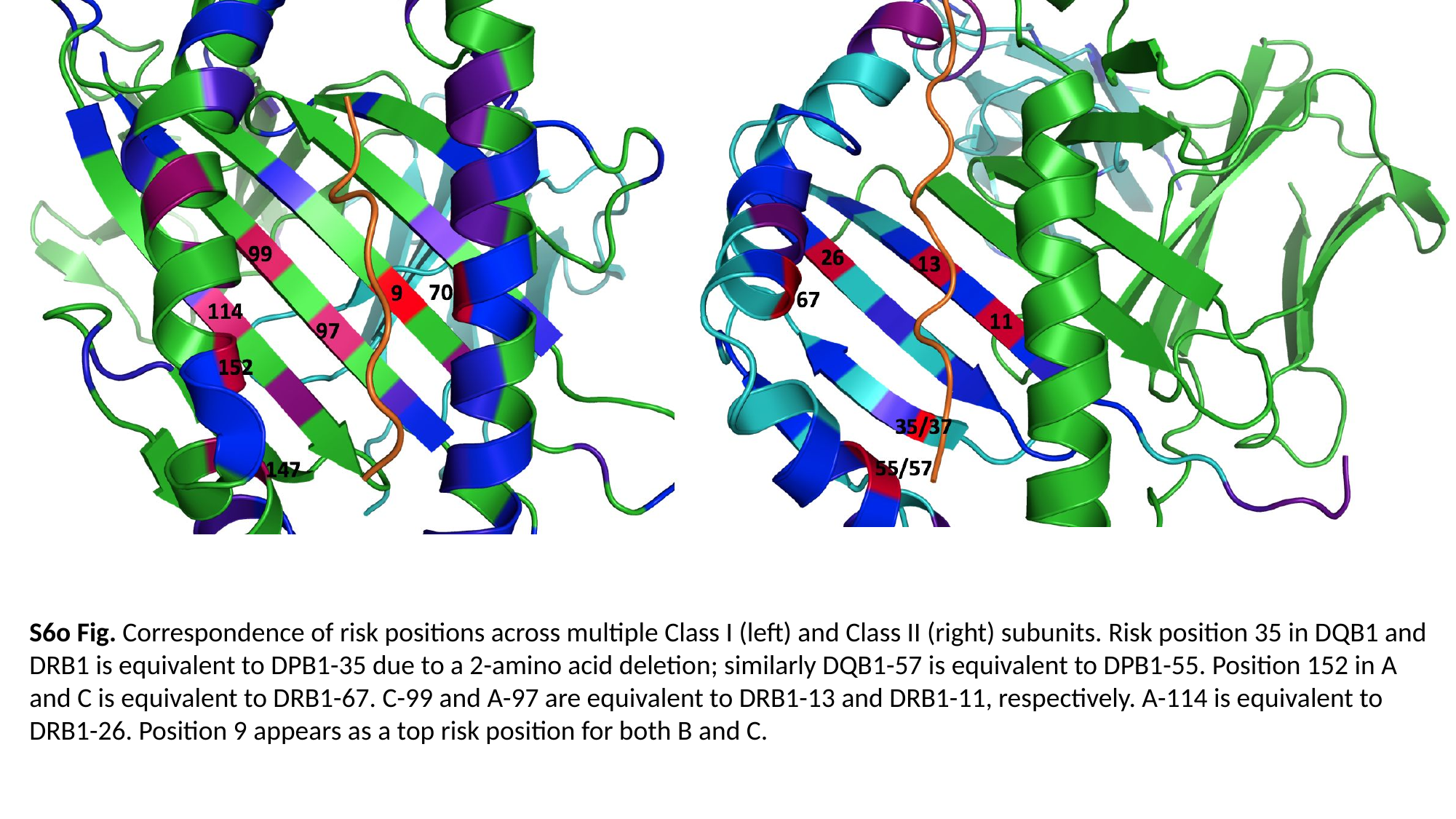

S6o Fig. Correspondence of risk positions across multiple Class I (left) and Class II (right) subunits. Risk position 35 in DQB1 and DRB1 is equivalent to DPB1-35 due to a 2-amino acid deletion; similarly DQB1-57 is equivalent to DPB1-55. Position 152 in A and C is equivalent to DRB1-67. C-99 and A-97 are equivalent to DRB1-13 and DRB1-11, respectively. A-114 is equivalent to DRB1-26. Position 9 appears as a top risk position for both B and C.
