# Supplementary material for: Amino acid signatures of HLA Class-I and II molecules are strongly associated with SLE susceptibility and autoantibody production in Eastern Asians
Source: PLoS Genet. 2019 Apr 25;15(4):e1008092. doi: 10.1371/journal.pgen.1008092 (PMC6504188; doi:10.1371/journal.pgen.1008092)
Supplement: S1 Text — (DOCX) [file pgen.1008092.s019.docx]

**S1 TEXT**

*Modeling MHC-peptide-T-cell receptor interactions*

Most immunogenic peptides are enriched in large, aromatic and charged residues; specifically, Trp, Phe, Ile and Glu are over-represented, and Gln, Ser, Met and Lys under-represented, in MHC antigens^24^. Given the enormous diversity (>180 known) of autoantibodies produced in SLE^26^, we modelled interactions between HLA alleles (and the T-cell receptor, TCR) to arbitrary peptides. We predicted MHC allele binding to peptides by computing statistical potential between the peptide-binding side-chains of the MHC and the MHC-binding side-chains of the peptide. Specifically, the process consisted of: 1) Identifying amino acid prevalence in peptide antigens for specific autoimmune diseases (both from experimental literature^24^ and from calculations in this study), 2) Tabulating *peptide* amino acids that favor immunogenic interactions with the MHC and TCR, and those that disfavor those interactions; 3) For each of the 20 possible naturally occurring amino acids (considered at an *MHC* binding-groove position), calculate its average interaction (using a common residue-residue interaction score ^27^) with the *peptide* “pro-immunogenic” residues as well as the “anti-immunogenic” residues; 4) Identify those *MHC* side-chains that would interact much better with the *peptide* “pro-immunogenic” residues than the “anti-immunogenic” ones – these are then considered as risk *MHC* binding-groove residues; 5) Identify those *MHC* side-chains that would interact much better with the *peptide* “anti-immunogenic” residues than the “pro-immunogenic” ones – these are then considered as protective *MHC* binding-groove residues. Note that this is a calculation of the average interaction of a potential MHC binding-groove side-chain with amino acids presented from the peptide being displayed to the MHC; it does not consider interactions of specific full *MHC* allele sequences with specific full *peptide* sequences. Such 3-dimensional modeling of MHC-peptide interactions can be performed, but we felt that the statistical treatment was preferable in this situation, given the extraordinary diversity of SLE autoantibodies; the difficulty in identifying specific displayed peptides from antigenic proteins; the huge diversity of TCR sequences and docking conformations on the MHC-peptide complex; and the incomplete understanding of how MHC-peptide-TCR interactions lead to success or failure of TCR activation.

SLE autoantigens are overwhelmingly positively charged (isoelectric point, pI range = [6.96, 11.66]; charge/residue: [0.00, 0.26]; **S10a Table**), whereas RA autoantigens are more negatively charged (pI: [4.98, 9.28]; charge/residue: [-0.04, 0.02]; **S10b Table**). A hallmark of RA is the presence of anti-citrullinated protein antibodies (ACPA)^28^. Citrullination deiminates arginine residues (removing their positive charge), leaving the side-chain neutral; poly-citrullinated proteins become very negatively-charged (pI: [3.87, 5.23]; charge/residue: [-0.13, -0.04]; **S9b Table**).

Based on these very large differences in autoantigen charge, we developed two variations of the immunogenicity model^24^: for RA, the model was used as is, and for SLE, positively charged amino acids were deemed immunogenic. Amino acids that interact particularly well with SLE-immunogenic side-chains (“SLE-MHC risk”) include: Trp/Phe/Tyr (Trp/Phe), Ile (Ile) and Asp/Glu (Arg) (**S10a Table**). These six amino acids (Trp/Phe/Tyr/Ile/Asp/Glu) together comprise 30% of human proteins. However, 13/20 most-risk side-chains fell in Trp/Phe/Tyr/Ile/Asp/Glu (**Table 2**); these six amino acids are enriched more than 2-fold over expectation. Conversely, of the amino acids predicted to be SLE-protective (Arg, Asn, Cys, Lys, Ser, Thr; comprising 33% of the human proteome), only one (*DRB1*-13Arg; in complete LD with *DRB1*-11Pro) fell on the list of the 20 most-risk positions (7-fold less than the expectation). Thus, the experimentally observed risk positions are over-enriched in amino acids predicted to be risk, and under-enriched in those predicted to be protective.

MHC risk residues have been mapped for several other autoimmune diseases, with the best known being rheumatoid arthritis (RA). Autoantigens in RA differ from those in lupus in several important aspects that give rise to different MHC risk alleles and residues. A marker for most (but not all) RA cases is the presence of anti-citrullinated protein antibodies (ACPA)^28^. Citrullination deiminates arginine residues (removing their positive charge), leaving the side-chain neutral and more hydrophobic. A number of proteins are naturally citrullinated as part of their normal function; however, the enzymes responsible (peptidylarginine deiminases) have not been detected in the thymus^29^, potentially preventing T-cell education about citrullinated peptides. Despite this, ACPA are not widely detected in healthy people. In RA, however, ACPA are frequently detected, most commonly against fibrinogen, vinculin, collagen, filaggrin, vimentin, and keratin, all of which are helical fibril-forming, structural components of the synovial membrane.

A slightly different version of the model was created for RA as well, differing only in the treatment of positively charged residues. Reassuringly, the RA-specific model also performed extremely well (6/7 risk residues captured, with the sole exception being associated solely with citrullination-negative RA). The small change to the treatment of basic residues led to large differences in the prediction of risk residues between SLE and RA. The four charged residues arginine, lysine, glutamate, and aspartate were reversed between SLE and RA: Arg, Lys SLE-protective and RA-risk; Glu, Asp SLE-risk and RA-protective. Large, hydrophobic residues (isoleucine, leucine, methionine, and phenylalanine) were risk-predicted in both diseases. Small, hydrophilic residues (asparagine, serine, and threonine) were protective-predicted in both diseases.

**REFERENCES**

1. Sun, C. *et al.* High-density genotyping of immune-related loci identifies new SLE risk variants in individuals with Asian ancestry. *Nat Genet* (2016).

2. Gharavi, A.G. *et al.* Genome-wide association study identifies susceptibility loci for IgA nephropathy. *Nat Genet* **43**, 321-7 (2011).

3. Okada, Y. *et al.* A genome-wide association study identified AFF1 as a susceptibility locus for systemic lupus eyrthematosus in Japanese. *PLoS Genet* **8**, e1002455 (2012).

4. Zhao, J. *et al.* A missense variant in NCF1 is associated with susceptibility to multiple autoimmune diseases. *Nat Genet* **49**, 433-437 (2017).

5. Tan, E.M. *et al.* The 1982 revised criteria for the classification of systemic lupus erythematosus. *Arthritis Rheum* **25**, 1271-7 (1982).

6. Hochberg, M.C. Updating the American College of Rheumatology revised criteria for the classification of systemic lupus erythematosus. *Arthritis Rheum* **40**, 1725 (1997).

7. Zhou, F. *et al.* Deep sequencing of the MHC region in the Chinese population contributes to studies of complex disease. *Nat Genet* **48**, 740-6 (2016).

8. Jia, X. *et al.* Imputing amino acid polymorphisms in human leukocyte antigens. *PLoS One* **8**, e64683 (2013).

9. Cook, S. & Han, B. MergeReference: A Tool for Merging Reference Panels for HLA Imputation. *Genomics Inform* **15**, 108-111 (2017).

10. Kim, K., Bang, S.Y., Lee, H.S. & Bae, S.C. Construction and application of a Korean reference panel for imputing classical alleles and amino acids of human leukocyte antigen genes. *PLoS One* **9**, e112546 (2014).

11. Okada, Y. *et al.* Risk for ACPA-positive rheumatoid arthritis is driven by shared HLA amino acid polymorphisms in Asian and European populations. *Human Molecular Genetics* **23**, 6916-6926 (2014).

12. Okada, Y. *et al.* Construction of a population-specific HLA imputation reference panel and its application to Graves' disease risk in Japanese. *Nat Genet* **47**, 798-802 (2015).

13. Raychaudhuri, S. *et al.* Five amino acids in three HLA proteins explain most of the association between MHC and seropositive rheumatoid arthritis. *Nat Genet* **44**, 291-6 (2012).

14. Willer, C.J., Li, Y. & Abecasis, G.R. METAL: fast and efficient meta-analysis of genomewide association scans. *Bioinformatics* **26**, 2190-1 (2010).

15. Won, S., Morris, N., Lu, Q. & Elston, R.C. Choosing an optimal method to combine P-values. *Stat Med* **28**, 1537-53 (2009).

16. Chen, Z. *et al.* A new statistical approach to combining p-values using gamma distribution and its application to genome-wide association study. *BMC Bioinformatics* **15 Suppl 17**, S3 (2014).

17. Robinson, J. *et al.* The IPD and IMGT/HLA database: allele variant databases. *Nucleic Acids Res* **43**, D423-31 (2015).

18. Thompson, J.D., Gibson, T.J. & Higgins, D.G. Multiple sequence alignment using ClustalW and ClustalX. *Curr Protoc Bioinformatics* **Chapter 2**, Unit 2 3 (2002).

19. Kumar, S., Stecher, G. & Tamura, K. MEGA7: Molecular Evolutionary Genetics Analysis Version 7.0 for Bigger Datasets. *Mol Biol Evol* **33**, 1870-4 (2016).

20. So, H.C., Gui, A.H., Cherny, S.S. & Sham, P.C. Evaluating the heritability explained by known susceptibility variants: a survey of ten complex diseases. *Genet Epidemiol* **35**, 310-7 (2011).

21. Lewontin, R.C. The Interaction of Selection and Linkage. I. General Considerations; Heterotic Models. *Genetics* **49**, 49-67 (1964).

22. Hardin, J.A. & Thomas, J.O. Antibodies to histones in systemic lupus erythematosus: localization of prominent autoantigens on histones H1 and H2B. *Proc Natl Acad Sci U S A* **80**, 7410-4 (1983).

23. Mitchell, J.B.O., Laskowski, R.A., Alex, A. & Thornton, J.M. BLEEP - Potential of mean force describing protein-ligand interactions: I. Generating potential. *Journal of Computational Chemistry* **20**, 1165-1176 (1999).

24. Calis, J.J. *et al.* Properties of MHC class I presented peptides that enhance immunogenicity. *PLoS Comput Biol* **9**, e1003266 (2013).

25. Lafer, E.M. *et al.* Polyspecific monoclonal lupus autoantibodies reactive with both polynucleotides and phospholipids. *J Exp Med* **153**, 897-909 (1981).

26. Yaniv, G. *et al.* A volcanic explosion of autoantibodies in systemic lupus erythematosus: a diversity of 180 different antibodies found in SLE patients. *Autoimmun Rev* **14**, 75-9 (2015).

27. Simons, K.T. *et al.* Improved recognition of native-like protein structures using a combination of sequence-dependent and sequence-independent features of proteins. *Proteins* **34**, 82-95 (1999).

28. Schellekens, G.A. *et al.* The diagnostic properties of rheumatoid arthritis antibodies recognizing a cyclic citrullinated peptide. *Arthritis Rheum* **43**, 155-63 (2000).

29. Vossenaar, E.R., Zendman, A.J., van Venrooij, W.J. & Pruijn, G.J. PAD, a growing family of citrullinating enzymes: genes, features and involvement in disease. *Bioessays* **25**, 1106-18 (2003).
